# Nitrene-Transfer Chemistry to Olefins Mediated by Co(III) Catalysts Supported by a Tetraamido Macrocycle

**DOI:** 10.1021/acs.inorgchem.6c02795

**Published:** 2026-09-13

**Authors:** Himanshu Bhatia, Jacob Green, Mia Ehrhart, Rohit G. Jadhav, Amitava Choudhury, Pericles Stavropoulos

**Affiliations:** Department of Chemistry, 14717 Missouri University of Science and Technology, Rolla, Missouri 65409, United States

## Abstract

A tetraamido macrocyclic ligand scaffold (L^1^H_4_) has been synthesized and metalated with Co­(III) to afford anionic metal complexes [Co^III^(L^1^)]^−^ (cations: K^+^, PPh_4_^+^) that are paramagnetic (*S* = 1) in solution and display semi-reversible reduction waves (Co^II/III^) and irreversible anodic events in cyclic voltammograms. These macrocyclic Co­(III) compounds have been examined as nitrene-transfer catalysts, involving iminoiodinane precursors (PhI=NR) and olefins, to reveal efficient aziridinations and/or allylic aminations, especially with sterically unencumbered styrenes. Mechanistic studies indicate that stepwise formation of two N–C bonds is operative in aziridinations, in which both polar and radical contributions are evident, in contrast to analogous nitrene-transfer chemistry displayed by [Co^III^(TAML^red^)]^−^, the latter relying on single-electron transfer between ligand and substrate. The generation of both mono- and bis-nitrene cobalt complexes or derivatives is indicated by ESI-MS, but their precise structural identity, electronic configuration, and relevance in nitrene-transfer chemistry remains an area for further exploration.

## Graphical abstract

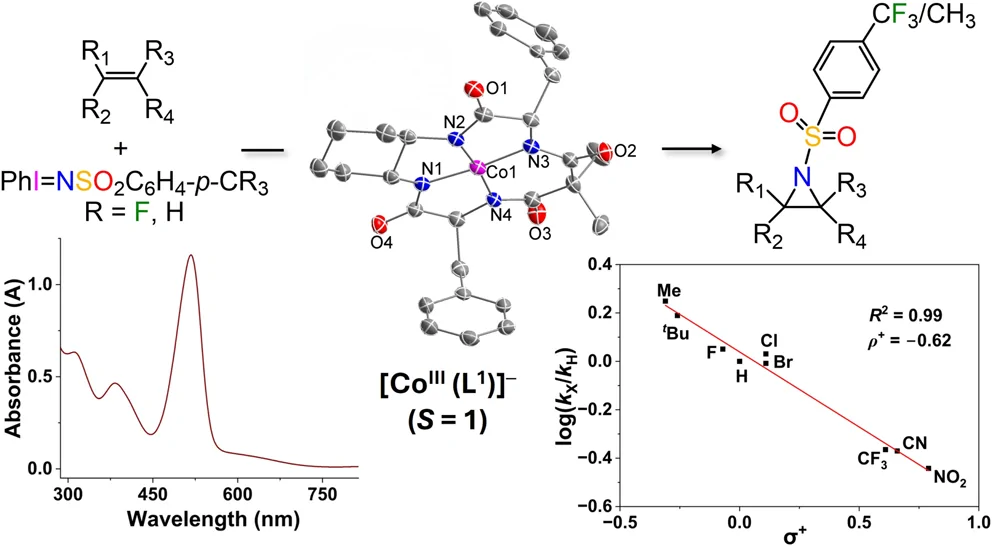

## Introduction

Nitrene-transfer to alkenes constitutes an atom- and energy-economic process that gives rise to aziridines and/or allylic amines, as well as derivatives thereof.^1^ Both classes of compounds are highly valued components of synthetic and natural products, and essential intermediates for the development of a wide range of bioactive or industrially important chemicals^2–4^ since they can guide the synthesis of structurally complex molecules via their multiprong reactivity.^5^ Whereas many methodologies have been advanced to synthesize aziridines and allylic amines,^2a,6^ the advent of nitrene-transfer to alkenes has provided operationally simple protocols that rely on an abundance of commercially available starting materials.^7^

Although some intriguing stoichiometric reactions between alkenes and nitrene precursors have been advanced,^8^ facilitated by light-driven or electrochemical induction,^9^ the vast majority of nitrene-transfer methodologies relies on catalytic approaches to guide reactivity and selectivity outcomes. Several organocatalytic^10^ and metal-mediated^11^ protocols have been introduced, the latter employing platinum-group^12–14^ or heavier coinage elements,^15,16^ but also increasingly engaging first-row transition metal catalytic sites.^17^ Hence, complexes containing middle/late first-row transition elements (Mn, Fe, Co, Ni, Cu)^18–23^ supported by a variety of ligand scaffolds (e.g., porphyrinoids, salens, bis-oxazolines, trispyrazolyl-borates/methanes, polypyridines) have given rise to powerful de novo or biologically inspired metal sites,^24^ to enable efficient and selective nitrene-transfer reactions vis-à-vis alkenes. A significant difference between nitrene/nitrenoid- and oxo-transfer technologies is the direction offered by the nitrene (NR) substituent, whose electrophilic and steric characteristics can influence reactivity and selectivity outcomes, as for instance in the case of activated (R = SO_2_R, CO_2_R, COR, carbamoyl, sulfamoyl)^25–29^ or nonactivated aziridines (R = H, alkyl, aryl, silyl).^30^

While middle/late transition elements such as Mn and Fe are well established to operate via high-valent M^*n*+^=NR oxidants (*n* ≥ 3) in their nitrene-transfer catalytic cycles, cobalt catalysts tend to be more intriguing with respect to the metal-bound oxidizing equivalents. In fact, many isolable Co=NR units have been assigned a variety of oxidation states (from II to V),^17^ although their nitrene-transfer capabilities vis-à-vis alkene feedstock proved to be modest at best.^31–41^

A significant breakthrough in catalytic reactivity was achieved with the introduction of a library of Co^II^(Por) catalysts that generate metalloradical Co^III^–nitrenoids [(Por)­Co^III^–^•^NR] or [(Por^•^)­Co^III^–(^•^NR)_2_], effecting stereo-, chemo- and site-selective aziridination of alkenes and amination of C–H bonds.^21^ The low-spin (*S* = 1/2) [(Por)­Co^III^–^•^NR] moieties exhibit significant spin density localized on the N atom,^21d,42^ and operate via a stepwise mechanism, initially forming a new N–C bond with alkenes and relocating the spin density on the distal carbon atom (Co^III^–N­(R)–C–^•^C−), followed by a barrierless recombination of the carbon-centered radical with the N atom to generate the product of aziridination.

Most relevant to the present work are Co­(III) compounds supported by the tetraamido macrocyclic ligand TAML, whose most commonly used scaffold is depicted in Figure 1. Compounds [Co^III^(TAML^red^)]^−^ and [Co^III^(TAML^sq^)], in which TAML (X = Cl) is either fully reduced or one-electron oxidized, have been shown to react with PhINNs to afford [Co^III^(TAML^q^)­(^•^NR)_2_]^−^ (*S* = 1) and [Co^III^(TAML^q^)­(^•^NR)] (*S* = 1/2), respectively (TAML^q^ = two-electron oxidized, diamagnetic ligand; Co^III^ site is low-spin, *S* = 0).^43^ These cobalt compounds can mediate nitrene-transfer from iminoiodinanes (PhINNs, PhINTs, PhINTces) to olefinic substrates (styrenes, 1-hexene, cyclohexene), affording products of aziridination and allylic amination.^21a^ Their mode of operation is considered to be unusual, since the initial N–C bond formation with styrene is accomplished in an asynchronous manner, involving initial single-electron transfer from styrene to the metal-nitrene, followed by a *nucleophilic* attack by the nitrene radical lone pair on the styrene radical cation.

**Figure 1:**
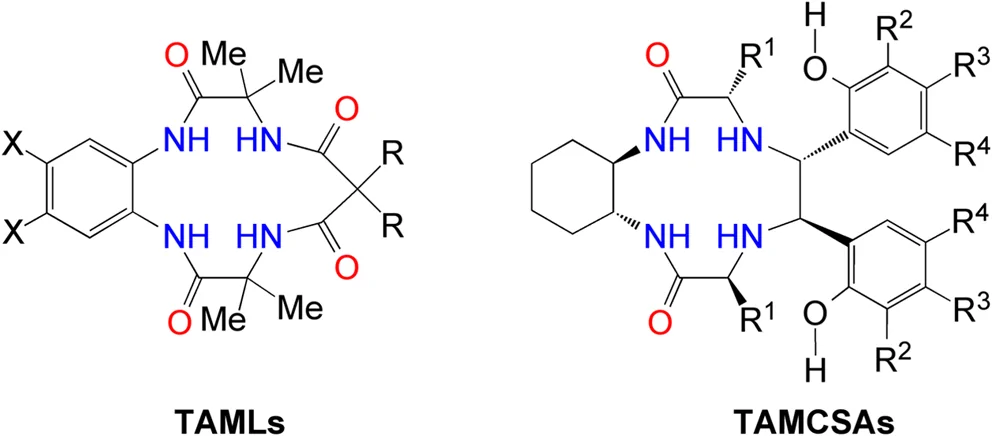
TAML and TAMCSA ligand families.

In the present work, we initiate an examination of a family of tetraaza macrocyclic ligands that had been originally constructed with an eye toward applications as chiral solvating agents (CSAs) for determination of enantiomeric excess by ^1^H NMR spectroscopy (TAMCSAs, Figure 1).^44^ This ligand family presents obvious analogies to the TAML scaffolds, but also incorporates new structural elements, chief among which are (i) the replacement of the redox non-innocent 1,2-diamidophenylene head spacer with the more robust 1,2-diamidocyclohexane group; and (ii) the integration of several stereocenters. Other head/tail spacer modifications have been previously reported in support of Fe­(III),^45^ Fe­(V),^46^ Co­(III)^47^ and Cu­(III)^48^ sites with nonchiral/open and (non)­chiral/cyclic ligand frameworks, in the context of oxo-transfer chemistry. In the present publication, a simplified tetraamidato version of the macrocyclic TAMCSA family is examined as a Co­(III)-supporting ligand scaffold, to investigate its comparative effectiveness and mode of action in mediating nitrene-transfer chemistry vis-à-vis alkene feedstock. Other attributes, for instance in support of C–H bond aminations and in enantioselective synthesis, will be explored with a wider range of metal compounds and TAMCSA scaffolds in future work.

## Results and Discussion

### Synthesis of Ligand and Co­(III) Compounds

The macrocyclic tetraamido ligand L^1^H_4_ was prepared in a three-step synthetic procedure from commercially available (1*R*,2*R*)-(−)-1,2-diaminocyclohexane and (2*S*)-2-amino-3-phenylpropanoic acid (l-phenylalanine), as shown in Scheme 1. The synthetic protocol for the first two steps (coupling of the Boc-protected l-phenylalanine with the chiral diamine, followed by deprotection) has been previously reported as part of the synthesis of various TAMSCAs,^44^ albeit of the opposite chirality. Closing of the macrocyclic ring has been readily accomplished with the assistance of dimethylmalonyl chloride, a precursor of a common spacer used in TAML chemistry.^49^ The resulting chiral ligand is fairly insoluble in many polar solvents, but can be crystallized from DMSO (vide infra).

**Scheme 1:**
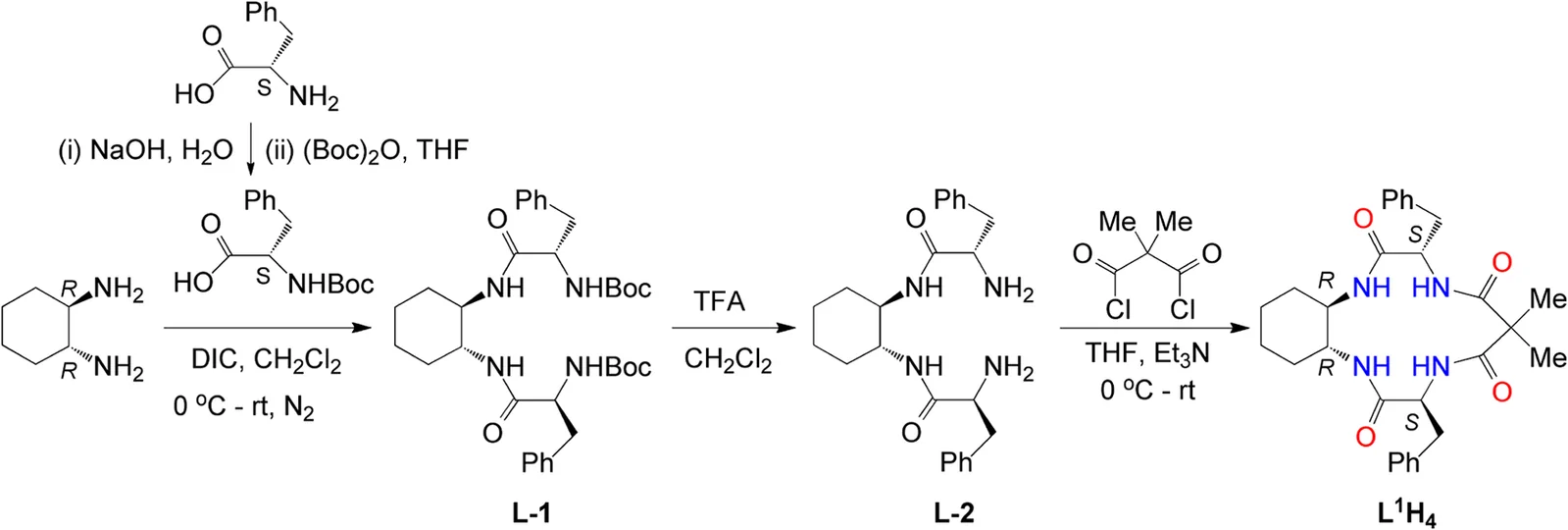
Synthesis of the Macrocyclic Tetraamido Ligand L^1^H_4_ Used in This Study

Two Co­(III) compounds were generated with ligand L^1^H_4_, featuring K^+^ and PPh_4_^+^ as counter-cations (Scheme 2). First, the ligand was deprotonated with KH (4 equiv) in *N*,*N*-dimethylacetamide (DMA),^50^ followed by metalation with CoCl_2_ (1 equiv) under inert atmosphere. The resulting brown-green solution was then exposed to air to readily afford purple solutions of the corresponding Co­(III) complex. A crystalline sample suitable for SCXRD analysis can be obtained upon slow diffusion of diethyl ether into a DMA solution of the Co­(III) compound at −35 °C, to afford polymeric [K­(DMA)_2_Co^III^(L^1^)] (**1**). The potassium cation can be replaced by PPh_4_^+^, by adding excess PPh_4_Cl to an aqueous solution of **1**, and further dissolving the precipitate in MeCN or DCM, following a previously pioneered protocol.^43,51^ Crystalline (PPh_4_)­[Co^III^(L^1^)] (**2**) can be obtained from slow diffusion of Et_2_O layered over MeCN (or from DCM/pentane).

**Scheme 2:**
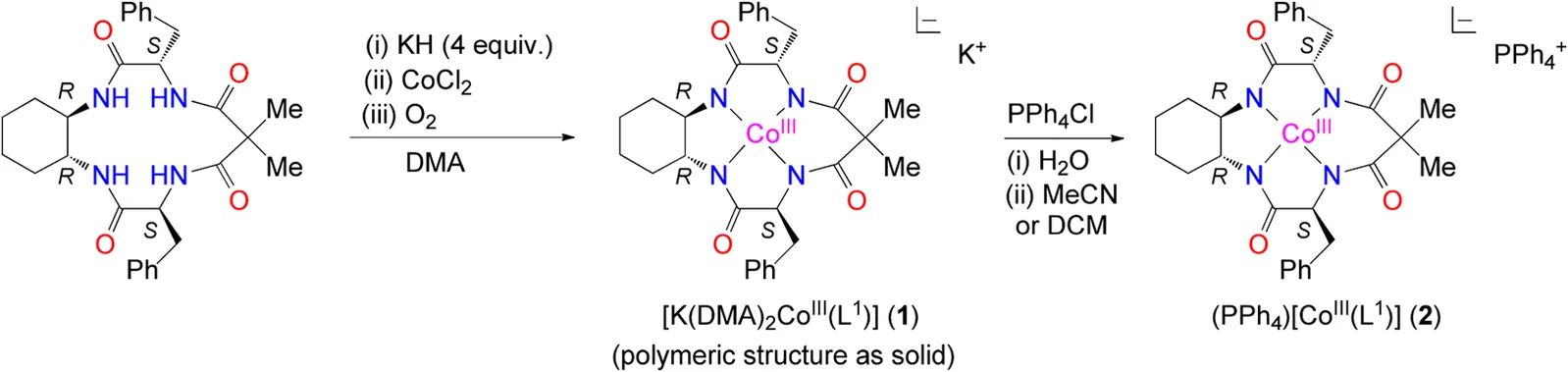
Synthesis of Anionic Co­(III) Compounds

### Solid-State Structures

The structure of ligand L^1^H_4_, crystallized from DMSO solutions (L^1^H_4_·3DMSO), consists of columnar stacks of monomers, arranged in an alternating head-to-tail orientation (Figure 2). Between monomers, a self-complementary network of four intermolecular hydrogen-bonding interactions^52^ exist between donor (N–H) and acceptor (O=C) moieties, as a result of a DADA array of sites (D = donor, A = acceptor) protruding from each side of the monomer. Hence, all eight heteroatoms (N, O) are engaged in individual hydrogen-bonding interactions, with the NH···O distance ranging from 2.141 to 2.364 Å, and the corresponding angle from 144.54 to 160.19°. In addition, potential C–H-π contacts (T-shaped) between monomers are detected, involving C(12)–H(12) and aromatic ring C(39)–C(44), as well as C(56)–H(56) and aromatic ring C(23)–C(28), at a distance of 2.69 and 2.77 Å vs the aromatic centroid, respectively.

**Figure 2:**
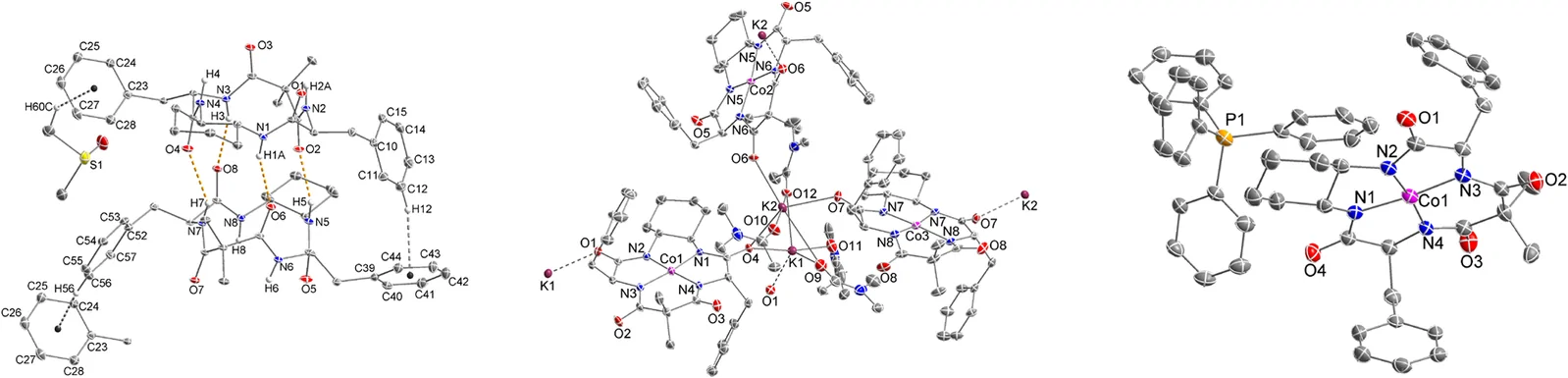
ORTEP diagrams of L^1^H_4_·3DMSO, [K­(DMA)_2_Co^III^(L^1^)] (**1**) and (PPh_4_)­[Co^III^(L^1^)] (**2**) drawn with 40% thermal ellipsoids. Selective interatomic distances (Å) and angles (°) for L^1^H_4_·3DMSO: N(1)···O(6) = 3.013(8), N(2)···O(5) = 3.135(8), N(3)···O(8) = 3.007(8), N(4)···O(7) = 2.952(8), N(5)···O(2) = 2.913(8), N(6)···O(1) = 2.993(8), N(7)···O(4) = 3.123(8), N(8)···O(3) = 2.973(8). For **1**: Co(1)–N(1) = 1.818(4), Co(1)–N(2) = 1.836(4), Co(1)–N(3) = 1.824(4), Co(1)–N(4) = 1.827(4), Co(2)–N(5) = 1.819(3), Co(2)–N(6) = 1.832(3), Co(3)–N(7) = 1.816(4), Co(3)–N(8) = 1.836(4), N(1)–Co(1)–N(2) = 86.21(17), N(3)–Co(1)–N(4) = 98.54(17), N(5)–Co(2)–N(5) = 86.7(2), N(6)–Co(2)–N(6) = 99.1(2), N(7)–Co(3)–N(7) = 87.0(2), N(8)–Co(3)–N(8) = 99.0(2). For **2**: Co(1)–N(1) = 1.816(3), Co(1)–N(2) = 1.815(3), Co(1)–N(3) = 1.830(3), Co(1)–N(4) = 1.825(3), N(1)–Co(1)–N(2) = 86.33(14), N(3)–Co(1)–N(4) = 98.69(13).

The molecular unit of [K­(DMA)_2_Co^III^(L^1^)] (**1**) assembles into extended chains in the lattice, featuring three distinct Co­(III) sites (Figure 2). The central Co(2) complex possesses a two-fold axis bisecting the cyclohexane ring and passing through the quaternary carbon of the dimethylmalonyl spacer. Two Co(1) and two Co(3) sites are arranged around the central Co(2) site in a propeller fashion, and are related by means of the central *C*_2_ axis noted above. Whereas the Co(1) sites do not possess any strict local symmetry, Co(3) centers exhibit a two-fold axis, similar to the one observed for Co(2) sites. The three Co sites interconnect via a dinuclear K_2_(DMA)_4_ assembly, featuring two terminal and two bridging DMA moieties, and also providing contacts to ligand-based carbonyl moieties. In particular, K(1) is associated with Co(1) sites via O(1) and O(4) carbonyl residues, whereas K(2) is connecting to Co(2) and Co(3) sites via O(6) and O(7) carbonyls, respectively. The chirality is preserved throughout the lattice by means of two mutually perpendicular screw axes (2_1_). Otherwise, the Co­(III) sites exhibit distorted square planar geometries with Co–N bond distances in the range of 1.816 to 1.837 Å, featuring shorter (1.816–1.819 Å) and slightly longer (1.824–1.836 Å) bonds for the five- and six-membered rings, respectively. These two ranges are not as distinct for the most distorted Co(1) site. As expected, the corresponding N–Co–N angles for the five- and six-membered rings (86.7(2)° and 99.1(2)°, respectively, for Co(2)) are substantially different.

The corresponding (PPh_4_)­[Co^III^(L^1^)] (**2**) is an uncomplicated ionic complex, devoid of any solvent in the lattice, featuring a discrete [Co^III^(L^1^)]^−^ anion (Figure 2). The distorted square planar Co­(III) site does not possess a strict *C*_2_ local axis, hence, both the five- and six-membered rings are slightly asymmetric (Co(1)–N(1) = 1.816(3), Co(1)–N(2) = 1.815(3) Å, N(1)–Co(1)–N(2) = 86.33(14)°; Co(1)–N(3) = 1.830(3), Co(1)–N(4) = 1.825(3) Å, N(3)–Co(1)–N(4) = 98.69(13)°). By comparison to the analogous (PPh_4_)­[Co^III^(TAML^red^)] compound reported by de Bruin and co-workers,^43^ the ligand field exerted in **2** is slightly stronger, presumably due to the presence of the 1,2-cyclohexanediamido spacer in **2** in lieu of the *o*-phenylenediamido spacer in the TAML analog.

A summary of crystallographic data for all compounds is listed in Table S1.

### Solution Behavior

Compound **2** displays a characteristic UV–vis absorption band at λ_max_ = 517 nm (ε = 10979) in MeCN, consistent with its red-purple color, although well-formed crystals have an orange-red appearance. ESI-MS data in the negative mode confirm the presence of the anion in MeCN (*m*/*z* = 559), accompanied by a small peak cluster at *m*/*z* = 575, consistent with a [Co­(L^1^) + O]^−^ mass, that becomes more prominent with extra residual water (Figure 3, left). A recent publication^53^ highlights the rich electrocatalytic chemistry of [Co^III^(TAML^red^)]^−^ (X = H, Figure 1) in the presence of water, revealing the presence of several mono- and bis-oxyl or hydroxyl species (albeit not the corresponding +16 mass) detected in voltammetry electrospray ionization mass spectrometry (VESI-MS) experiments. We consider that the *m*/*z* = 575 peak is of similar provenance, under the influence of the ESI-MS applied voltage. In the presence of D_2_O, the peak shifts to *m*/*z* = 576, thus suggesting a mass consistent with [Co­(L^1^)–H + OD]^−^, most likely due to oxo atom insertion into a C–H bond of the ligand (Figure 3, right), as suggested in Co­(III)-TAML chemistry.^53^

**Figure 3:**
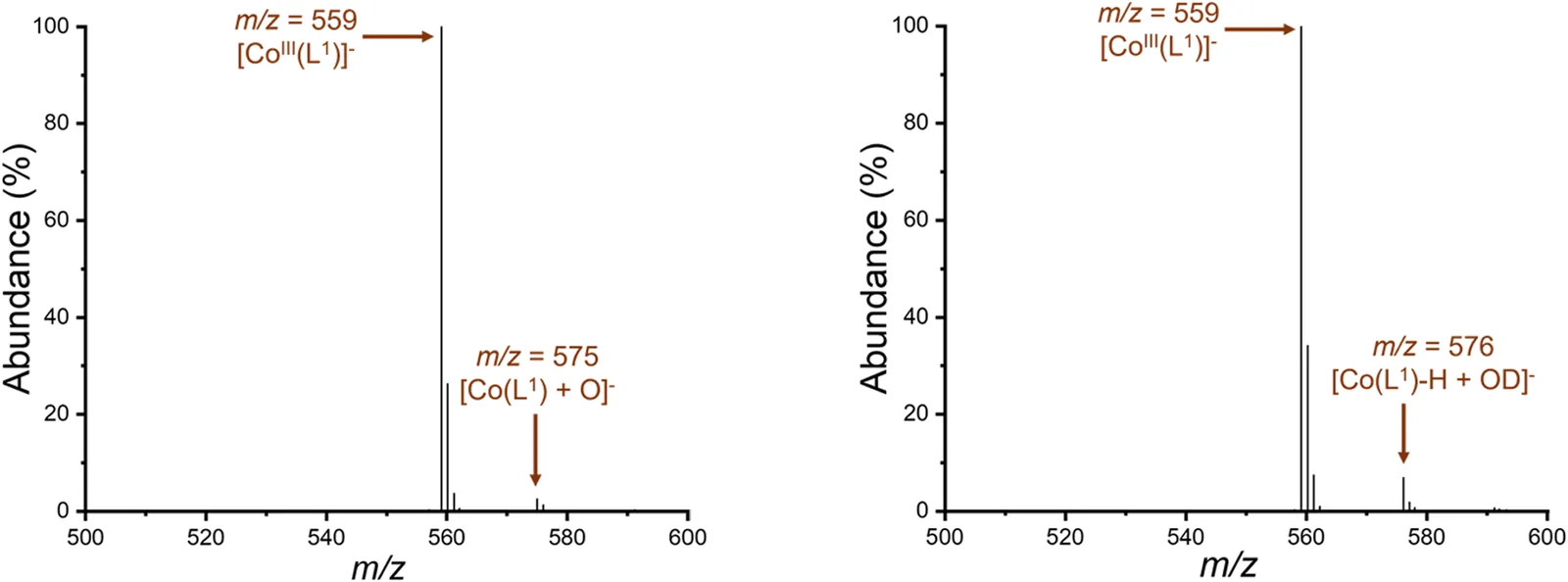
ESI-MS spectra (negative mode) of (PPh_4_)­[Co^III^(L^1^)] (**2**) in MeCN before (left) and after (right) addition of D_2_O.

Evaluation of the effective magnetic moment of **2** in MeCN by means of the Evans’ method^54^ (μ_eff_ = 2.89 μ_B_) indicates a triplet ground state (*S* = 1), in agreement with that reported for analogous [Co^III^(TAML)]^−^ compounds.^43,51^ On the other hand, the electrochemical behavior of compound **2**, as revealed by cyclic voltammetry in MeCN (or CH_2_Cl_2_), differs significantly to that observed for PPh_4_[Co^III^(TAML^red^)].^43^ A semi-reversible wave at *E*_1/2_ = −1.33 V vs Fc^+^/Fc is noted for **2** in MeCN (for scan rates ranging from 5–300 mV s^–1^: Δ*E*_p_ = 67–99 mV, *i*_pa_/*i*_pc_ = 0.628–0.904, with anodic and cathodic currents being proportional to the square root of the scan rate; Figure 4 (left) and S2) and assigned to the Co­(III)/Co­(II) couple (*E*_1/2_ = −1.48 V vs Fc^+^/Fc in DCM), at more negative potentials than those observed for the TAML analog, given the ligand field strength of **2**. Oxidative events for **2** are characterized by an irreversible anodic wave at *E*_p,a_ = 0.85 V vs Fc^+^/Fc (Figure 4, right), in sharp contrast to the reversible oxidation peaks (*E*_1/2_ = +0.53, +1.13 V vs Fc^+^/Fc) observed for the TAML compound.^43^ The latter is the result of successive one-electron oxidations of the 1,2-diamidophenylene fragment of the TAML ligand scaffold, which is absent in **2**. Attempts to oxidize compound **2** either chemically (NO^+^, NO_2_^+^) or electrochemically have resulted in the formation of intractable species.

**Figure 4:**
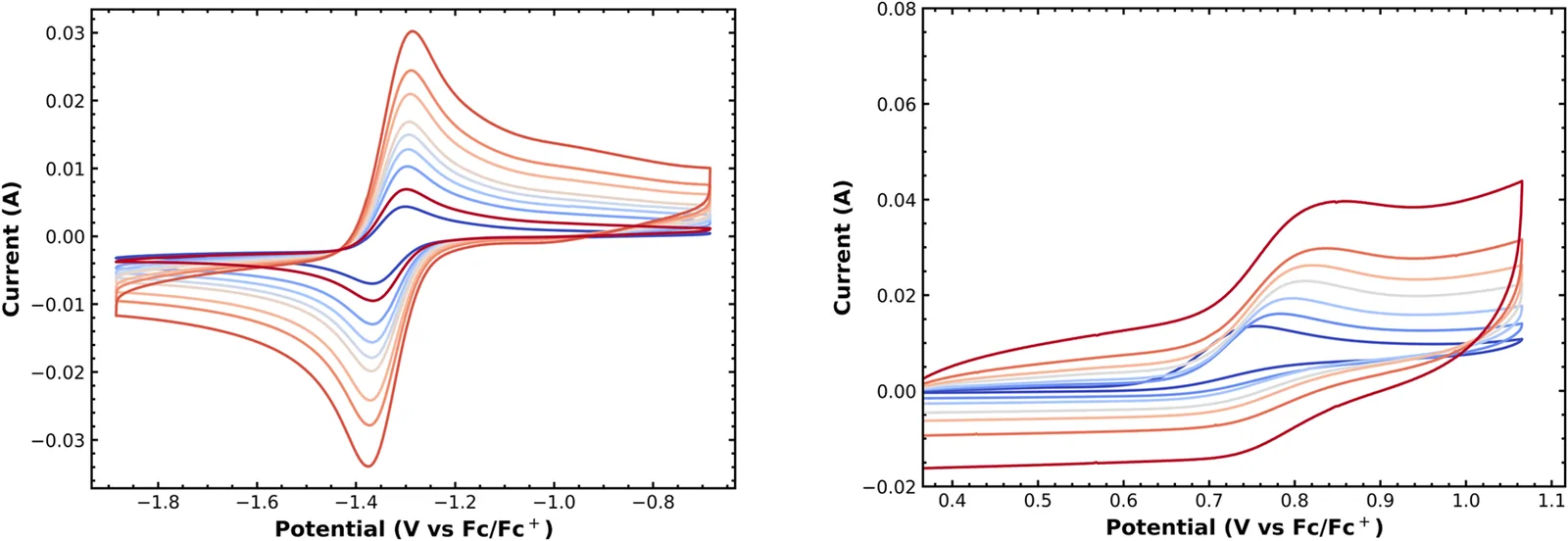
Cyclic voltammograms of (PPh_4_)­[Co^III^(L^1^)] (**2**) in acetonitrile (cathodic and anodic events), containing 0.1 M (^*n*^Bu_4_N)­(PF_6_) at scan rates from 5 to 300 mV s^–1^.

### Catalytic Studies

#### Aziridination of Styrene

The catalytic activity of Co­(III) compounds **1** and **2** was initially evaluated in nitrene-transfer reactions versus styrene. Two nitrene precursors were employed, the common PhI=NTs (Ts = tosyl) and the more electron-deficient PhI=NSO_2_–C_6_H_4_-*p*-CF_3_. The latter has been recently used in aziridinations of unactivated alkenes, either as an iminoiodinane under metal-free conditions in HFIP,^8b^ or as an azide engaged in photoredox catalysis.^55^ Notably, the known PhI=NSO_2_–C_6_H_4_-*p*-CF_3_ is significantly more soluble in organic solvents than polymeric PhI=NTs, and can be crystallized from MeOH/H_2_O (ORTEP in Figure S1).

The catalytic reaction was first examined by employing PhI=NTs (1.0 equiv) as a nitrene source along with excess styrene (8 equiv) in the presence of catalyst **1** (2.5 mol %) at 25 °C. Owing to the low solubility of **1** in the solvents used for catalytic reactions, the requisite amount of catalyst **1** was first dissolved in MeCN, and then evacuated to dryness prior to use. Both halogenated (1,2-dichloroethane, dichloromethane, chlorobenzene, α,α,α-trifluorotoluene) and non-halogenated (acetonitrile) solvents were explored, in the presence of 5 Å molecular sieves (entries 1–5, Table S2). The yields obtained vary in a narrow range (55–62 %), with the highest yield recorded in PhCF_3_. Increasing the catalyst loading to 5 mol % in PhCF_3_ does not offer any significant gains (entry 6). The much more soluble catalyst **2** (2.5 mol %) mediates the reaction of styrene with PhI=NTs in an even narrower range of yields (60–64 %, entries 7–11). Once again, working with 5 mol % of catalyst **2** in PhCF_3_ does not offer any yield improvements (entry 12). On the other hand, switching to the more electron-deficient PhI=NSO_2_–C_6_H_4_-*p*-CF_3_ elevates the reaction yield in all solvents examined (67–74 %, entries 13–18). Higher catalyst loading (5 mol %) provides slightly higher yields in PhCF_3_ (79%), but lower loading (2.0 mol %) suppresses the yield (entries 19, 20). Styrene aziridination was only observed in the presence of catalyst. Overall, the nitrene-transfer capabilities of **2** with respect to PhI=NSO_2_–C_6_H_4_-*p*-CF_3_ are similar to those reported for (PPh_4_)­[Co^III^(TAML^red^)] and PhI=NNs (Ns = −SO_2_–C_6_H_4_-*p*-NO_2_) in the aziridination of styrene at slightly higher temperature (35 °C),^21a^ but **2** seems to be more insensitive to the solvent used. Moreover, the TAML-supported Co­(III) reagent reportedly provides moderate styrene aziridination yields with PhI=NTs (33% in DCM).^21a^ Given the expense associated with the ligand synthesis, catalyst loadings of 2.5 mol % were employed for further studies.

#### Aziridination of *p*-Substituted Styrenes

Further evaluation of the catalytic activity of reagents **1** and **2** was undertaken with a panel of *p*-substituted styrenes, featuring both electron-donating and electron-withdrawing substituents (Table 1). The solvent PhCF_3_ was selected for further experimentation, given the slightly higher yields obtained with styrene and the known stability offered to various iminoiodinanes in this solvent.^56^ Both catalysts **1** and **2** (2.5 mol %) were explored with PhINTs under the conditions noted for the aziridination of styrene, affording similar yields with respect to electron-rich styrenes (entries 1–5). Alkoxy-substituted aziridines tend to be unstable under catalytic conditions (or during purification), hence, the observed yields are suppressed. With electron-deficient styrenes (entries 6–9), catalyst **2** provides superior yields, potentially reflecting the high solubility of **2** in PhCF_3_ (**1** is sparingly soluble). Further assessment of catalytic activity was thus explored with catalyst **2**. With most para-substituted styrenes, the more electron-deficient and soluble PhI=NSO_2_–C_6_H_4_-*p*-CF_3_ afforded better yields than those obtained with PhI=NTs (entries 1–7). Marginally lower yields were observed with the strongly deactivating CF_3_ and NO_2_ substituents (entries 8, 9). With respect to (PPh_4_)­[Co^III^(TAML^red^)] (2.5 or 5.0 mol % vs PhI=NNs in DCM),^21a^ catalyst **2** (2.5 mol % vs PhI=NSO_2_–C_6_H_4_-*p*-CF_3_ in PhCF_3_) provides slightly better yields with most *p*-substituted styrenes. Of note, although all catalytic reactions are conducted over 12 h, most of them reach completion in 2–4 h, especially with electron-rich substrates.

**Table 1:**
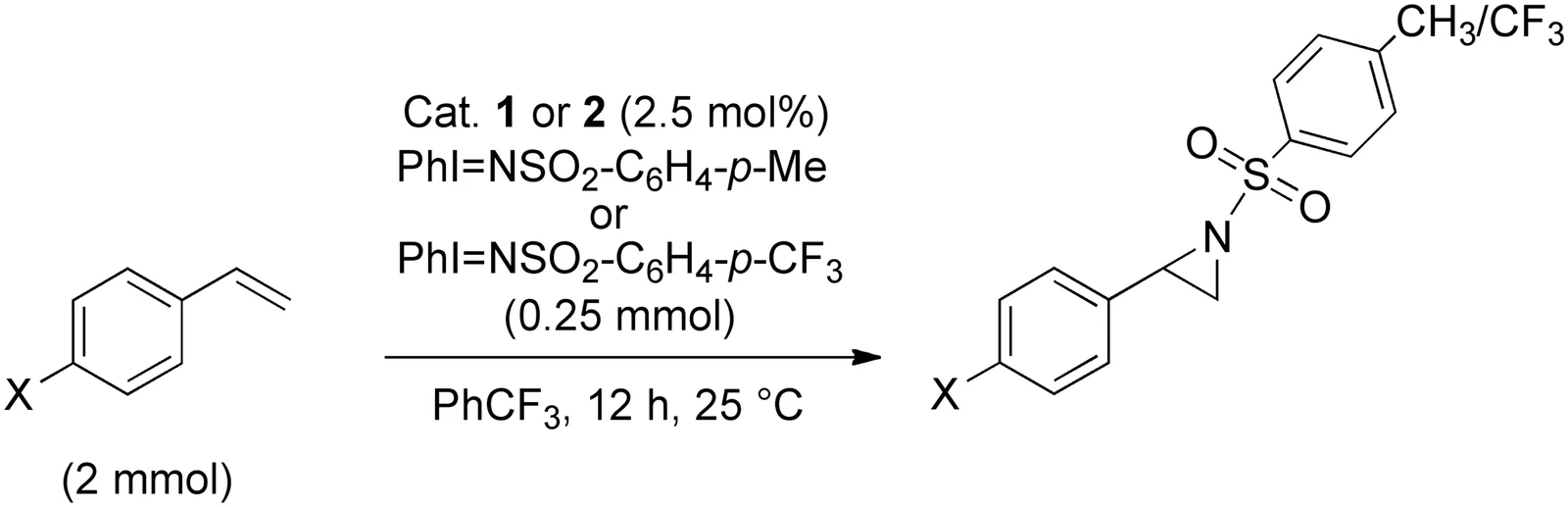
Yields of Aziridination of Para-Substituted Styrenes by PhI=NTs and PhI=NSO_2_–C_6_H_4_-*p*-CF_3_ Mediated by Catalysts **1** and **2** ^a^

Entry No.	Product	PhI=NTs Yield (%) Catalyst 1/2	PhI=NSO_2_-C_6_H_4_-*p*-CF_3_ Yield (%) Catalyst 2
1.	X = H	62/64	74
2.	X = Me	59/63	73
3.	X = ^t^Bu	60/60	69
4.	X=OMe	40/44	40
5.	X=O^t^Bu	41/39	40
6.	X = Cl	58/65	74
7.	X = F	58/67	72
8.	X = CF_3_	30/54	46
9.	X = NO_2_	35/52	50

a Catalyst **1** or **2**, 0.00625 mmol (2.5 mol %); PhI=NR, 0.25 mmol; olefin, 2.0 mmol; MS 5 Å, 20 mg; PhCF_3_, 0.30 mL; 25 °C; 12 h; NMR yields

#### Aziridination of Other Aromatic and Aliphatic Alkenes

Catalyst **2** was further explored with a panel of aromatic olefins and PhI=NTs or PhI=NSO_2_–C_6_H_4_-*p*-CF_3_ in PhCF_3_ (Table 2), under the conditions noted above. Among all substrates examined, β-substituted styrenes and derivatives thereof tend to be most productive (entries 1–6), especially if the β-substituent is not sterically demanding. Thus, the aziridination of *cis*-β-Me-styrene (entry 1) with PhI=NSO_2_–C_6_H_4_-*p*-CF_3_ affords both *cis* and *trans-*aziridines (≈2:1 ratio) in a combined yield of 62%, whereas the *trans* analog (entry 2) provides exclusively the corresponding *trans*-aziridine in marginally lower yields. The same reactions with PhI=NTs are less productive (44% with *cis*-β-Me-styrene; 40 % with *trans*-β-Me-styrene), reflecting the trend noticed with electron-rich styrenes. In addition, the aziridination of *cis*-β-Me-styrene is associated with poorer retention of stereochemistry (*cis*/*trans*-aziridine ≈ 1:1), as anticipated for the closing of the aziridine ring with a less electrophilic nitrene. Similar trends are observed for the aziridination of *cis*- and *trans*-stilbene, but with much suppressed yields (<20%, entries 3, 4), presumably due to the steric bulk of the phenyl group. For these substrates, low yields of epoxides are also detected with PhI=NSO_2_–C_6_H_4_-*p*-CF_3_, as previously noted with [Co^III^(TAML^red^)]^−^/PhI=NSO_2_–C_6_H_4_-*p*-NO_2_ in the presence of residual water.^57^ Nitrene-transfer to *trans*-β-benzyl-styrene (entry 5) affords both aziridination and allylic/benzylic amination products in an approximate ratio of 1:1, featuring higher combined yields for the more electron-deficient nitrene. On the other hand, both nitrenes provide the same moderate yields for the aziridination of the more sterically demanding 1,2-dihydronaphthalene, with only trace amounts of allylic amination being observed (entry 6). Surprisingly, nitrene-transfer to α-substituted (Me, Ph) styrenes (entries 7, 8) is associated with low yields, indicating that significant steric interactions with the ligand framework are operative. The reaction of α-Me-styrene (entry 7) and PhI=NTs affords the aziridine (main) and the allylic amine (minor), albeit at combined yields that do not exceed 20%. Notably, the same substrate with NSO_2_–C_6_H_4_-*p*-CF_3_ provides exclusively allylic amine and β-hydroxy amine, most likely due to facile aziridine-ring opening. On the other hand, the reaction of α-Ph-styrene with PhI=NTs (entry 8) provides enamine (major) and β-hydroxy amine (minor), whereas the aziridine is present in low yields (combined yield: 29%). The presence of enamine and β-hydroxy amine suggests that a common origin of these products may be a precursor benzylic carbocation generated upon initial nitrene addition to the β-carbon of the styrene; β-hydrogen elimination and nucleophilic attack by residual water can furnish the two products, respectively. Aziridine-ring opening and rearrangement may provide an alternative pathway for the enamine, as previously proposed for nosyl aziridine.^58^ Indeed, the corresponding reaction with PhI=NSO_2_–C_6_H_4_-*p*-CF_3_ affords only the enamine in 21% yield. The reaction of 2,4,6-trimethylstyrene with PhI=NTs (entry 9) affords the aziridine as a single product in moderate yields, reflecting the usual steric and electronic challenges associated with this substrate (orthogonal arene/alkene orientation).^59^ Interestingly, the reaction with PhI=NSO_2_–C_6_H_4_-*p*-CF_3_ affords very low yields of aziridine, but unidentified byproducts of aziridine-ring opening are also present.

**Table 2:**
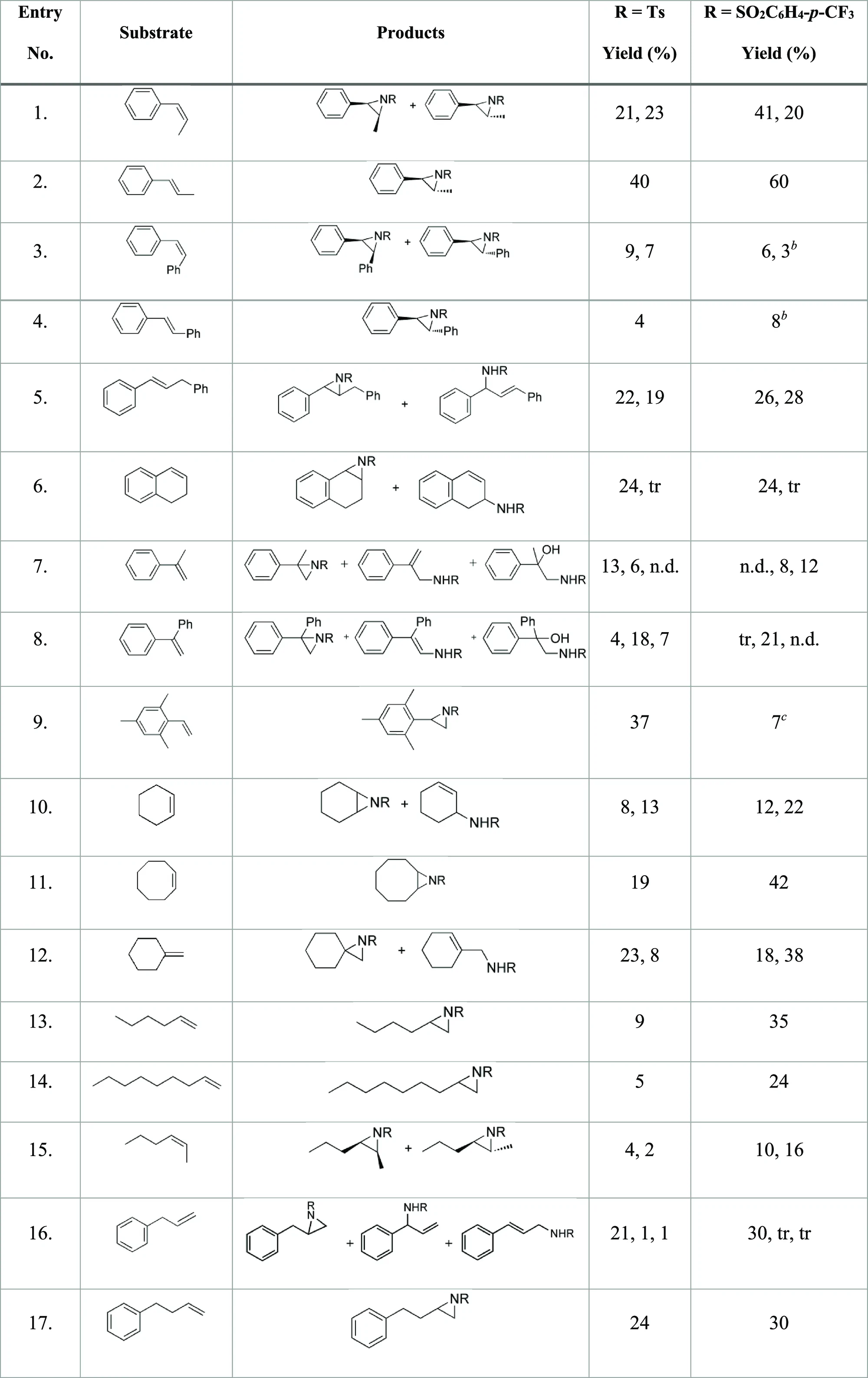
Yields of Aziridination and Amination Products of Various Olefins by PhI=NTs and PhI=NSO_2_–C_6_H_4_-*p*-CF_3_ Mediated by Co­(III) Catalyst **2** ^a^

a Co­(III) catalyst **2**, 0.00625 mmol (2.5 mol %); PhINTs or PhI=NSO_2_–C_6_H_4_-*p*-CF_3_, 0.25 mmol; olefin, 2.0 mmol; MS 5 Å, 20 mg; solvent, 0.30 mL; 25 °C; 12 h. NMR yields.

b Low yields of epoxides are also detected.

c Additional unidentified products are present

As expected, more modest yields are obtained for aliphatic alkenes (entries 10–17), with PhI=NSO_2_–C_6_H_4_-*p*-CF_3_ delivering better yields than those observed with PhI=NTs, as noted with aromatic alkenes. Cycloalkenes, such as cyclohexene (entry 10) and *cis*-cyclooctene (entry 11), afford aziridine and allylic amine, the latter being the major product for cyclohexene and absent for *cis*-cyclooctene. Notably, computational work has shown that the mechanistically relevant epoxidation of cyclooctene, which is known to afford only the epoxide, precludes formation of the allylic hydroxylation product due to an unfavorable σ_C–H_/π_C=C_ orbital overlap.^60^ Similarly, methylenecyclohexane (entry 13) affords aziridine and allylic amine (single isomer) in moderate yields, with aziridine being the major product with PhI=NTs but minor with PhI=NSO_2_–C_6_H_4_-*p*-CF_3_, potentially due to more efficient aziridine-ring opening in the latter case. On the other hand, linear alkenes, such as 1-hexene (entry 13) and 1-nonene (entry 14), afford almost exclusively aziridine in modest but considerably better yields with PhI=NSO_2_–C_6_H_4_-*p*-CF_3_. The mechanistically diagnostic *cis*-2-hexene (entry 15) provides very low yields of *cis*- and *trans*-aziridine in a ratio of 2:1 with PhI=NTs, and higher yields (combined *cis*/*trans* yield: 26%) with PhI=NSO_2_–C_6_H_4_-*p*-CF_3_, although the *cis*/*trans*-aziridine ratio (∼0.6) favors the *trans* product in the latter case. Finally, allylbenzene (entry 16) and 4-phenyl-1-butene (entry 17) afford largely the aziridination product with the former substrate (minor amounts of allylic amines are observed) and exclusively with the latter substrate, given the distance of the alkene from the aromatic ring. Product yields reported for all entries in Table 2 are associated with satisfactory mass balances once the presence of free amine (TsNH_2_ or *p*-CF_3_–C_6_H_4_–SO_2_NH_2_) is taken into account.

Competitive aziridinations of equimolar amounts (8 equiv each) of styrene versus 1-hexene, allylbenzene, and 4-phenyl-1-butene (Table 3) by PhI=NTs or PhI=NSO_2_–C_6_H_4_-*p*-CF_3_ (1 equiv), mediated by catalyst **2** (2.5 mol %) in MeCN, favor the aromatic over the aliphatic olefins by a significant margin. Interestingly, the more reactive nitrene (NSO_2_–C_6_H_4_-*p*-CF_3_) is also the more chemoselective, but differences in the solubility of the nitrene precursors may not permit a meaningful comparison.

**Table 3:**
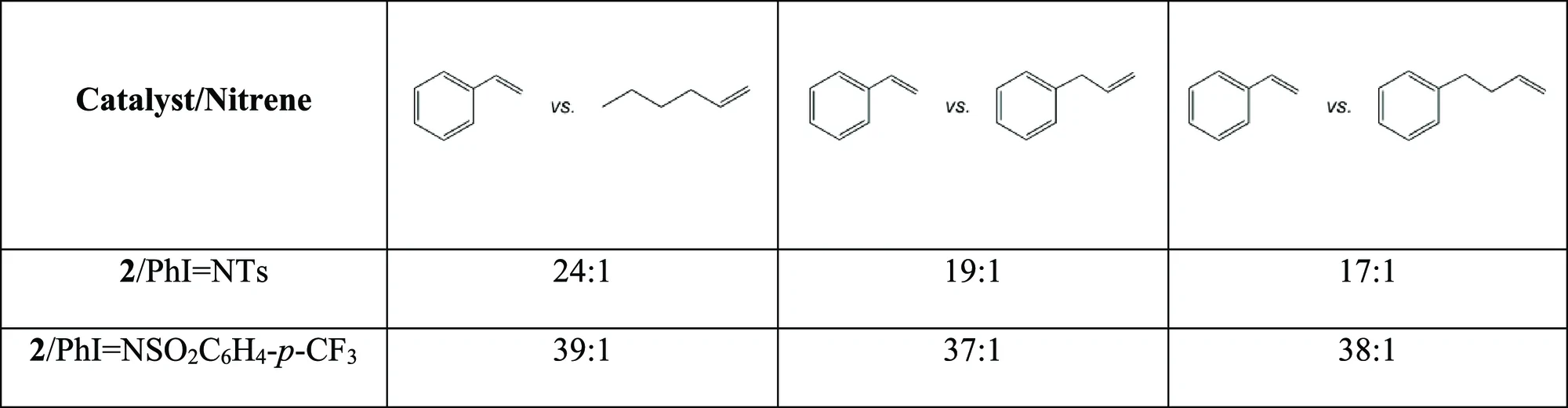
Competitive Aziridination of Styrene vs 1-Hexene, Allylbenzene and 4-Phenyl-1-Butene

### Mechanistic Studies

#### Hammett Plots

To start evaluating substrate-centered events upon addition of the putative metal-nitrene to olefins, competitive aziridination reactions were conducted between styrene (8 equiv) and a 4-X-styrene (8 equiv, X = ^*t*^Bu, Me, F, Cl, Br, CN, CF_3_, NO_2_) with PhI=NTs or PhI=NSO_2_–C_6_H_4_-*p*-CF_3_ (1 equiv) in the presence of catalyst **2** (2.5 mol %) in PhCF_3_. The reaction was quenched after 2.5 h, and the ratio of aziridination products was quantified by ^1^H NMR (CD_3_CN) to provide *k*_X_/*k*_H_ data (Table S3). Surprisingly, the reaction with PhI=NTs (1 equiv), featuring a combined aziridination yield of ∼60% and only TsNH_2_ as a byproduct, did not offer any satisfactory linear free energy correlation of log­(*k*_X_/*k*_H_) as a function of σ_p_, σ^+^ or the dual polar/radical σ_mb_/σ_JJ_^•^ parameters (Figure S3). As a matter of fact, the aziridination of several 4-X styrenes (X = ^*t*^Bu, Me, F, Cl, Br) is significantly faster that the aziridination of styrene and within a narrow margin (*k*_X_/*k*_H_ = 1.7–1.9). Only strong electron-withdrawing substituents (X = CN, CF_3_, NO_2_) provide values of *k*_X_/*k*_H_ < 1. A somewhat better but still unsatisfactory linear correlation is obtained by applying the Yukawa-Tsuno equation,^61^ which emphasizes resonance contributions. It is possible that for the fast reacting substrates, the turnover-limiting step shifts toward the generation of metal-nitrene(s), given the heterogeneous nature of the reaction between metal sites and PhI=NTs. On the other hand, competitive aziridinations of the same panel of olefins with PhI=NSO_2_–C_6_H_4_-*p*-CF_3_ afford an acceptable free energy correlation of log­(*k*_X_/*k*_H_) as a function of the polar parameter σ_p_ (ρ_p_ = −0.69, *R* ^2^ = 0.94, Figure S4), which is further improved by employing the resonance-sensitive parameter σ^+^ (ρ^+^ = −0.62, *R* ^2^ = 0.99, Figure 5, left). Application of Jiang’s dual parameter approach, relying on both polar (σ_mb_) and spin-delocalization (σ_JJ_^•^) parameters,^62^ does not provide any further improvements (ρ_mb_ = −0.64, ρ_JJ_^•^ = 0.49, *R* ^2^ = 0.98, Figure 5, right), given the preponderance of polar over spin-delocalization effects (|ρ_mb_/ρ_JJ_^•^| = 1.31). Therefore, a small positive charge develops en route to the transition state upon metal-nitrene attack to the olefin, but radical contributions are also not insignificant. Notably, the corresponding ρ values reported for the competitive aziridination of styrene vs various 4-X-styrenes by PhI=NNs in the presence of (PPh_4_)­[Co^III^(TAML^red^)] in DCM (ρ^+^ = −0.78, ρ^+^/ρ_JJ_^•^ = −0.80/0.14)^21a^ are similar to those obtained here with respect to the polar parameter, but significantly suppressed with regards to the radical component (|ρ^+^/ρ_JJ_^•^| = 5.71), indicating that the TAML-supported catalyst is operating heavily via polar effects.

**Figure 5:**
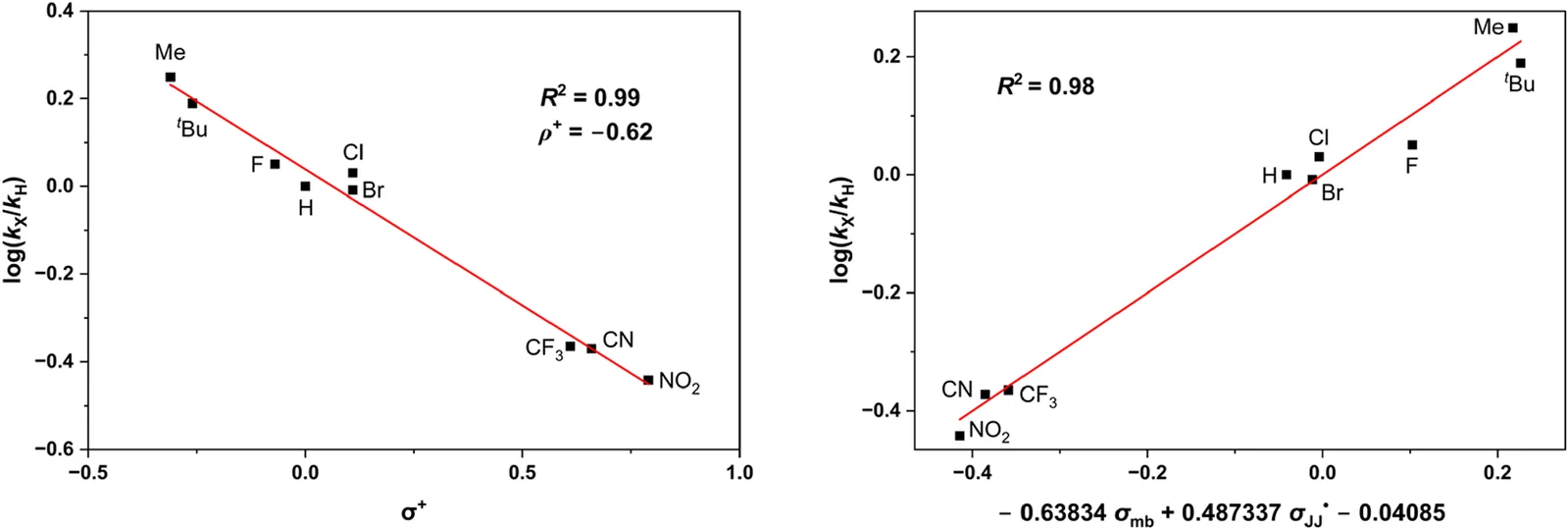
Linear free energy correlation of log­(*k*_X_/*k*_H_) vs σ^+^ and σ_mb_/σ_JJ_^•^ for the aziridination of a panel of 4-X-styrenes (X = ^*t*^Bu, Me, H, F, Cl, Br, CN, CF_3_, NO_2_) by PhI=NSO_2_–C_6_H_4_-*p*-CF_3_ mediated by catalyst **2** in PhCF_3_.

### Kinetic Isotope Effects

To evaluate the olefinic carbon that might be the site of the initial nitrene attack, we performed competitive aziridination of styrene vs *cis*-β-*d*_1_-styrene or α-*d*_1_-styrene (8 equiv each) by PhI=NTs or PhI=NSO_2_–C_6_H_4_-*p*-CF_3_ (1 equiv each) mediated by catalyst **2** (2.5 mol %) in PhCF_3_. The ratio of protio/deuterio-containing aziridines (*k*_H_/*k*_D_) was evaluated by ^1^H NMR to afford a KIE value of 0.88 (±0.02) with PhI=NTs and 0.92 (±0.02) with PhI=NSO_2_–C_6_H_4_-*p*-CF_3_ at 25 °C (Scheme 3). The corresponding reaction with α-*d*_1_-styrene afforded KIE values of 1.03 (±0.02) and 1.02 (±0.02) with PhI=NTs and PhI=NSO_2_–C_6_H_4_-*p*-CF_3_, respectively. The inverse KIE values suggest that the β-styrenyl carbon is engaged in the initial attack of the metal-nitrene to the styrene, probably with an earlier transition state for the more electron-deficient nitrene. The *cis*/*trans* ratio of β-deuterated aziridines was also evaluated by ^1^H- and ^2^H NMR, either from the same competitive experiment or by employing *cis*-β-*d*_1_-styrene as a single substrate. High levels of stereoretention were obtained (*cis*/*trans* = 17 (≈94:6, NTs), 27 (≈96:4, NSO_2_–C_6_H_4_-*p*-CF_3_)), indicating slightly faster aziridine-ring-closing with the more electron-deficient nitrene.

**Scheme 3:**
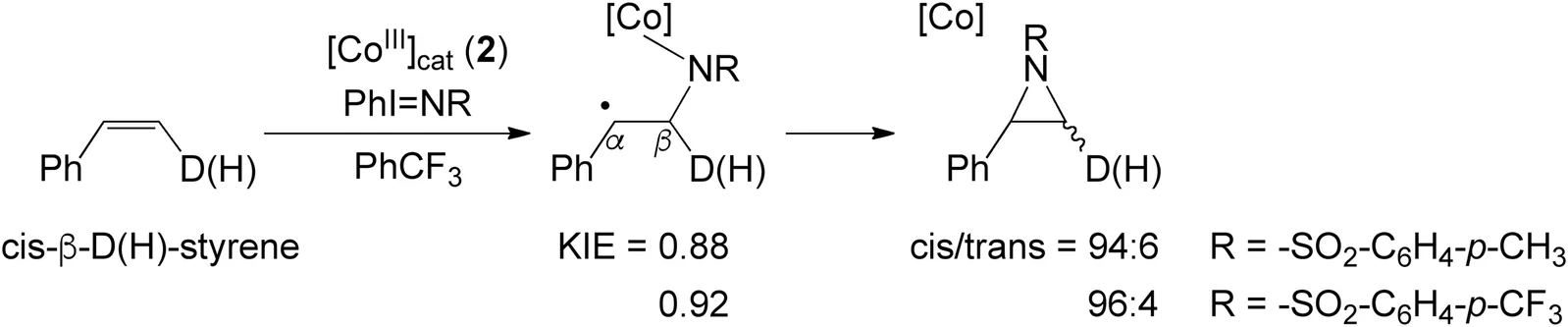
Putative Cobalt-Nitrene Addition to Styrene and Aziridine Ring Formation

#### Stoichiometric Reactions of Catalyst 2 with Nitrene Precursors

(PPh_4_)­[Co^III^(L^1^)] (**2**) was allowed to react with PhI=NTs or PhI=NSO_2_–C_6_H_4_-*p*-CF_3_ (2–12 equiv) in MeCN for 15 min under ambient conditions, and the mixture, after removal of any remaining PhI=NR solid, was analyzed by UV–Vis. UV–Vis data (Figures 6 and S5) indicate progressive reduction of the intensity of the absorption bands of catalyst **2** (312, 383, 517 nm) along with significant increase of absorption intensity below 400 nm and minor decrease above 600 nm, without involvement of clean isosbestic points. Importantly, the 517 nm (λ_max_) absorption band of the catalyst slightly shifts to 518 nm, along with disappearance of the 312 and 383 nm bands, thus indicating generation of new species at higher added equivalents of nitrene precursor.

**Figure 6:**
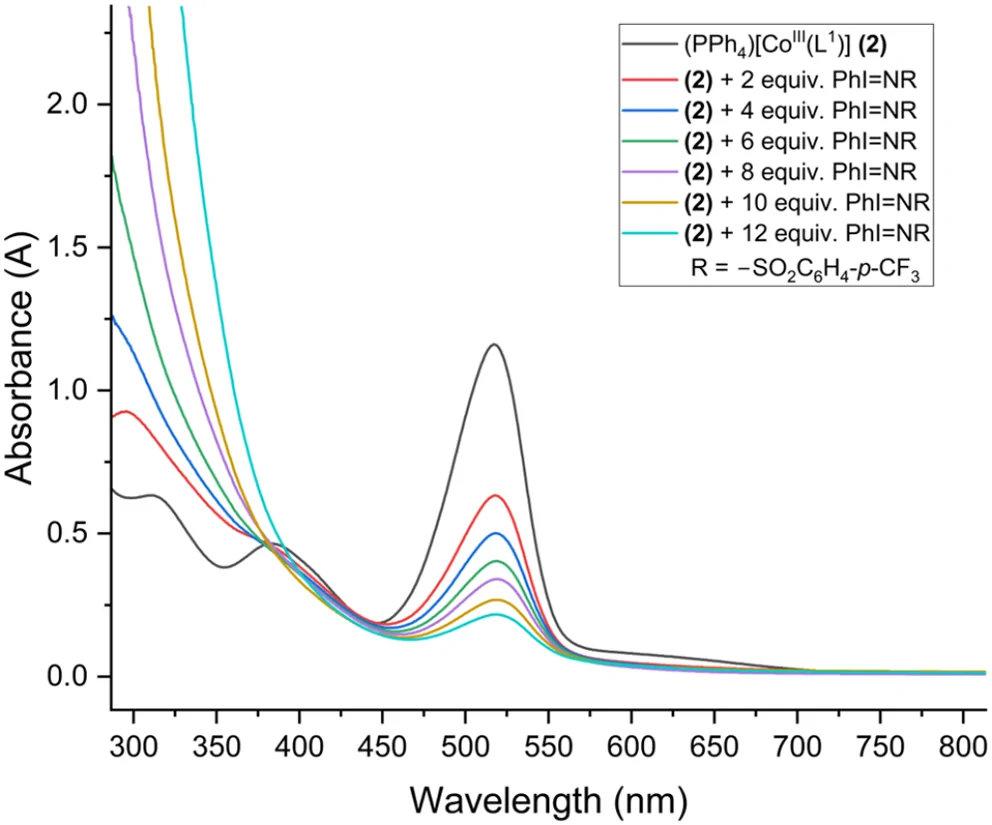
UV–Vis spectra of catalyst **2** upon addition of various equivalents of PhI=NSO_2_–C_6_H_4_-*p*-CF_3_ in MeCN.

ESI-MS (negative mode) spectra obtained after a reaction of **2** with 2 equiv of nitrene precursor for 10 min, indicate that catalyst **2** (*m*/*z* = 559, [Co^III^(L^1^)]^−^) remains the major peak cluster, but new peaks start emerging at masses consistent with [Co­(L^1^) + NR]^−^ and [Co­(L^1^) + 2 NR]^−^ anions (*m*/*z* = 728, 897 (with NTs); 782, 1005 (with NSO_2_–C_6_H_4_-*p*-CF_3_), Figure 7). The abundance of these new peaks by comparison to that of catalyst **2** is significantly more pronounced with the more reactive -NSO_2_–C_6_H_4_-*p*-CF_3_. In addition, the relative abundance between [Co­(L^1^) + NR]^−^ and [Co­(L^1^) + 2 NR]^−^ favors the former with NTs and the latter with NSO_2_–C_6_H_4_-*p*-CF_3_. With 5 equiv of PhI=NR, the peak clusters for [Co­(L^1^) + NR]^−^ and [Co­(L^1^) + 2 NR]^−^ figure more prominently by comparison to the peak cluster for catalyst **2** (*m*/*z* = 559), whereas, with 10 equiv of PhI=NR, catalyst **2** can hardly be observed after 10 min into the reaction (Figure S6). In addition, organic products deriving from nitrene “turnover” are observed, with masses corresponding to the free amine [*p*-R-C_6_H_4_–SO_2_NH_2_ + e^–^] (*m*/*z* = 171 (R = CH_3_), 225 (R = CF_3_)) and dimeric [(*p*-R-C_6_H_4_–SO_2_)_2_N]^−^ (*m*/*z* = 324 (R = CH_3_), 432 (R = CF_3_)).

**Figure 7:**
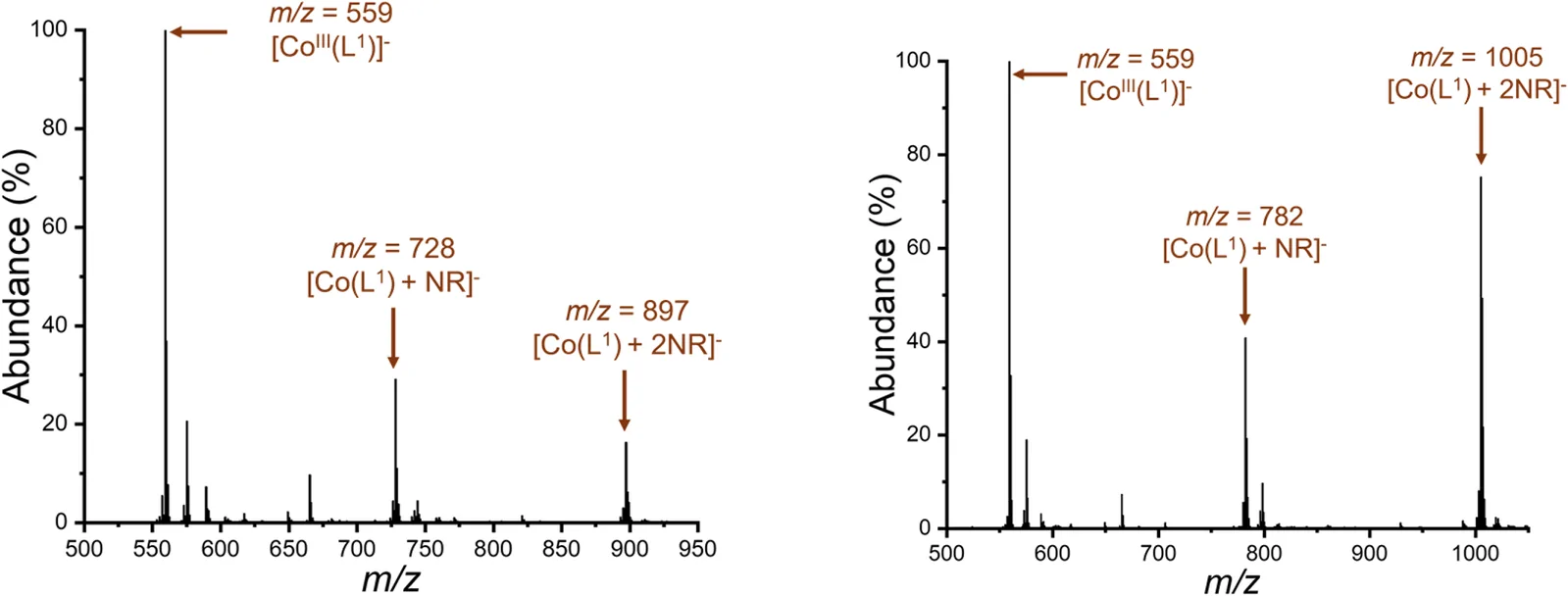
ESI-MS spectra of (PPh_4_)­[Co^III^(L^1^)] (**2**) after addition of 2 equiv of PhI=NTs (left) or PhI=NSO_2_–C_6_H_4_-*p*-CF_3_ (right) in MeCN (10 min).

The structural identity of the [Co­(L^1^) + NR]^−^ and [Co­(L^1^) + 2 NR]^−^ ions was further examined in the presence of D_2_O. For [Co­(L^1^) + NR]^−^, the peak shifts almost exclusively (>90%) to *m*/*z* = 729 (NTs) and 783 (NSO_2_–C_6_H_4_-*p*-CF_3_), hence the mass is consistent with [Co­(L^1^)–H + N­(D)­R]^−^, potentially denoting nitrene insertion into a C–H bond of the ligand (Figure 8). The structural identity of the [Co­(L^1^) + 2 NR]^−^ ion is more difficult to discern, primarily because its abundance is significantly diminished upon exposure to D_2_O, whereas that of [Co­(L^1^) + NR]^−^ is enhanced. Application of the DGet! analysis package for calculating the degree of deuterium enrichment^63^ provides levels of deuteration that vary from D0 to D3 (NTs: D0, 30.3 %; D1, 10.0 %; D2, 48.7 %; D3, 11.0 %. NSO_2_C_6_H_4_-*p*-CF_3_: D0, 27.0 %; D1, 12.5 %; D2, 37.5 %; D3, 23.0 %). Notably, the bis-deuterated adduct prevails with both putative nitrenes, indicating possible double nitrene insertion into C–H bonds ([Co­(L^1^)–2H + 2 N­(D)­R]^−^). However, significant amounts of non-deuterated nitrene adducts are also evident, followed by mono-deuterated products (presumably due to single nitrene C–H bond insertion), tentatively suggesting retention of authentic Co=NR entities in the D0 and D1 adducts. Tri-deuterated adducts of unclear provenance are also noted. The presence of active metal-nitrene entities is in tentative agreement with results from a styrene aziridination experiment, in which catalyst **2** (0.0125 mmol) and 10 equiv of PhI=NTs were stirred in MeCN (1.0 mL) for 10 min, followed by filtration of excess PhI=NTs (insoluble in MeCN) and addition of the supernatant to excess styrene (1.0 mL), affording 58% yield of the corresponding aziridine versus the catalyst.

**Figure 8:**
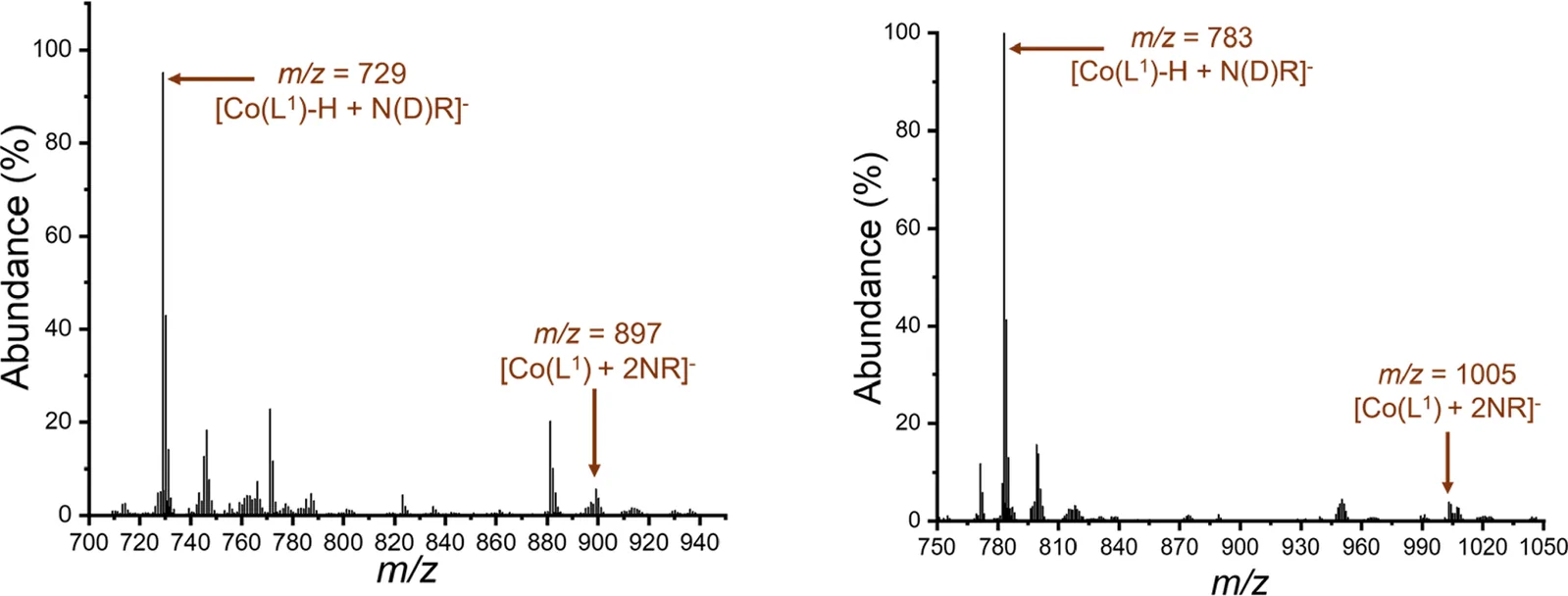
ESI-MS spectra of (PPh_4_)­[Co^III^(L^1^)] (**2**) after addition of 5 equiv of PhI=NTs (left) or PhI=NSO_2_–C_6_H_4_-*p*-CF_3_ (right) in MeCN (10 min) in the presence of D_2_O (5% per volume).

Whereas at the present time we do not have independent confirmation that the observed species represent authentic metal-nitrenes and derivatives thereof, we note the close similarities and differences with analogous mono- and bis-nitrene radical species isolated in Co­(III)-TAML chemistry.^21a,43^ The latter enjoy superior stability due to the redox-active nature of the TAML framework, but also undergo hydrolysis to the corresponding metal oxo/oxyl species followed by slow oxo insertion into ligand-based C–H bonds in the absence of substrates.^53,57^ On the other hand, the present **2**/PhI=NR combination gives rise to apparently more reactive mono- and bis-NR addition products, engaging in fast NR-insertion into ligand moieties. Whether the initial NR addition products are solely metal-bound or potentially involve ligand participation (e.g., bridging between the metal and a ligand residue) will be the subject of further studies.^64^

## Conclusions

The following are the major findings and insights garnered from the present study:

i. A novel macrocycle (L^1^H_4_) has been constructed, capitalizing on previous engagements in the development of a family of tetraaza macrocycles employed as chiral solvating agents. The macrocyclic ring generates a tetraamido ligand scaffold assembled by coupling the commercially available chiral building blocks (1*R*,2*R*)-(−)-1,2-diaminocyclohexane and l-phenylalanine (1:2 ratio), and using dimethylmalonyl as a ring-closing spacer.
ii. Metalation with cobalt, affords Co­(III) anionic sites, [Co^III^(L^1^)]^−^, with K^+^ (**1**) and PPh_4_^+^ (**2**) as counter-cations. These cobalt­(III) sites are analogous to those encountered in the well-explored [Co^III^(TAML^red^)]^−^ compounds, but differ inasmuch as they feature the potentially redox-innocent 1,2-diamido-cyclohexane spacer (in lieu of phenylene) and also incorporate four stereocenters. Compound (PPh_4_)­[Co^III^(L^1^)] is characterized by an *S* = 1 ground state, not unlike that found with the TAML analog, resulting in paramagnetically shifted NMR spectra. On the other hand, the compound displays distinct redox behavior, as revealed by cyclic voltammetry, since an irreversible anodic wave is observed at high voltages, as opposed to two reversible, ligand-centered oxidation events noted for [Co^III^(TAML^red^)]^−^.
iii. Initial studies of nitrene-transfer to styrene, featuring two iminoiodinane precursors (PhI=NSO_2_–C_6_H_4_–CR_3_, R = H, F) and mediated by Co­(III) complexes **1** and **2**, indicate that aziridination yields are consistently higher with the more electron-deficient nitrene and, unlike [Co^III^(TAML^red^)]^−^, rather insensitive to the solvent employed. Catalyst loadings can be reduced to 2.5 mol % without significant loss in yield, and compound **2** tend to be more productive given its superior solubility. These observations also hold for the aziridination of a panel of para-substituted styrenes, with the exception of electron-deficient styrenes (*p*-CF_3_-styrene, *p*-NO_2_-styrene) that give marginally better yields with PhI=NTs.
iv. The aziridination of a wider range of aromatic olefins reveals that yields are very sensitive to steric encumbrance, especially when even small groups occupy ortho-, α-, and to a lesser extent β-styrenyl positions. Yields with aliphatic alkenes are more suppressed, but tend to be superior with the more electron-deficient nitrene. As usual, aromatic alkenes are significantly more reactive than aliphatic alkenes in competitive aziridination reactions (e.g., styrene/1-hexene: 24:1 (**2**/NTs), 39:1 (**2**/NSO_2_–C_6_H_4_-*p*-CF_3_)). Overall, the present catalytic system affords practicable yields only with unencumbered styrenes.
v. Mechanistic investigations (Hammett analysis) reveal that both polar and spin-delocalization effects apply to the aziridination of para-substituted styrenes by PhI=NSO_2_–C_6_H_4_-*p*-CF_3_, mediated by **2**, denoting the development of a small positive charge en route to the transition state, in conjunction with radical contributions. In sharp contrast, spin-delocalization effects constitute a minimal component in the turnover-limiting step of styrene aziridinations facilitated by [Co^III^(TAML^red^)]^−^, indicating a distinct mechanistic pathway. Evaluation of kinetic isotope effects (KIE) suggests that the initial Co-nitrene attack occurs at the β-styrenyl position (N–C_β_ bond formation), followed by fast ring closure (N–C_α_ bond formation), given the high levels of stereoretention in the aziridination of *cis*-β-d_1_-styrene. ESI-MS data suggest that mono- and bis-NR addition species are generated in solution, but their structure, electronic configuration and relative engagement in nitrene-transfer remain unclear, given their apparent fast insertion into ligand residues.

Future work will address remaining mechanistic intricacies with the assistance of a library of TAMCSA-type ligands and first-row transition metals, along with applications to asymmetric aziridinations of high-yielding styrenes.

## Experimental Section

### General Considerations

All operations were performed under anaerobic conditions under a pure dinitrogen atmosphere, using Schlenk techniques on an inert gas/vacuum manifold or in a drybox (O_2_, H_2_O < 1 ppm). Anhydrous diethyl ether, methylene chloride, 1,2-dichloroethane, acetonitrile, hexane, pentane, benzene, toluene, α,α,α-trifluorotoluene, dimethylformamide, dimethylacetamide, and dimethyl sulfoxide were purchased from Sigma-Aldrich (Millipore). Solvents were degassed by three freeze-pump-thaw cycles. Unless otherwise noted, all other reagents were purchased at the highest purity available or synthesized according to literature procedures noted in the text.

^1^H, ^13^C and ^19^F NMR spectra were recorded on Varian XL-400, Varian INOVA/UNITY 400 MHz Unity Plus and a Varian 300 Unity Plus NMR spectrometers. IR spectra were obtained on a PerkinElmer 883 IR spectrometer and FT-IR spectra on Nicolet Nexus 470 and 670, Magna 750 FT-IR ESP and Shimadzu IR-Affinity-1 spectrometers. UV–vis spectra were obtained on a Shimadzu UV-2600i Plus UV–VIS spectrophotometer. Microanalyses were done on an in-house PerkinElmer 2400 CHN analyser. Safety Statement: *The use of KH in N,N-dimethylacetamide (DMA) can be hazardous*.^50^

### Synthesis of Chiral Macrocyclic Ligand L^1^H_4_

#### L-1

The Boc-protected tetraamide precursor (Scheme 1) was synthesized according to a modified literature procedure.^65^ A solution of *N*,*N′*-diisopropylcarbodiimide (DIC) (2.52 g, 20 mmol) in dried CH_2_Cl_2_ (30 mL) was dropwise added to a mixture of (1*R*,2*R*)-(−)-1,2-diaminocyclohexane (1.08 g, 9.5 mmol) and *N*-Boc-protected l-phenylalanine (5.31 g, 20 mmol) in dried CH_2_Cl_2_ (45 mL) in an ice-salt bath under a dinitrogen atmosphere. The mixture was stirred over 16 h and filtered. The filtered cake was washed with CH_2_Cl_2_ three times (3 × 10 mL). The combined organic phase was dried over anhydrous Na_2_SO_4_. The solvent was removed under vacuum, and the residue was purified by column chromatography using hexanes/ethyl acetate (2:1 v/v) on silica gel to afford the pure compound as a white solid (4.04 g, 70 %). ^1^H NMR (400 MHz, CDCl_3_): δ = 7.17–7.32 (m, 10H), 6.23 (br, 2H), 5.30 (br, 2H), 4.15–4.31 (m, 2H), 3.44–3.58 (m, 2H), 3.01–3.23 (m, 2H), 2.75–3.00 (m, 2H), 1.84 (d, *J* = 11.4 Hz, 2H), 1.68 (d, *J* = 8.36 Hz, 2H), 1.36 (s, 18H), 1.17–1.28 (m, 2H), 0.9–1.11 (m, 2H). ^13^C NMR (101 MHz, CDCl_3_): δ = 172.1, 155.9, 137.2, 129.5, 128.7, 126.9, 80.3, 56.0, 53.8, 38.4, 32.0, 28.5, 24.7.

#### L-2

The diamidodiamine precursor (Scheme 1) was synthesized following a literature protocol.^66^ CF_3_COOH (3.0 mL) was added to the Boc-protected precursor L-1 (1.18 g, 1.8 mmol) in 10.0 mL of CH_2_Cl_2_. The mixture was allowed to react for 1.0 h, and was then diluted with CH_2_Cl_2_ (100 mL) and basified to pH 9–10 with 0.50 N NaOH. The organic layer was separated, dried over anhydrous Na_2_SO_4_ and evaporated to give the desired diamidodiamine as an off-white powder (0.603 g, 82%). ^1^H NMR (400 MHz, CDCl_3_): δ *=* 7.15–7.36 (m, 12 H), 3.59–3.71 (m, 2H), 3.45–3.56 (m, 2H), 3.25–3.23 (m, 2H), 2.46–2.58 (m, 2H), 1.99 (d, *J* = 12.8 Hz, 2H), 1.75 (d, *J* = 7.7 Hz, 2H), 1.53 (br, 4H), 1.28–1.37 (m, 2H), 1.15–1.27 (m, 2H). ^13^C NMR (101 MHz, CDCl_3_): δ = 174.7, 138.3, 129.3, 128.9, 126.9, 57.1, 53.3, 41.8, 32.4, 24.8.

#### L^1^H_4_

Under dinitrogen, the acyclic diamidodiamine L-2 (0.650 g, 1.59 mmol) and Et_3_N (0.354 g, 3.5 mmol) were dissolved in THF (80 mL) in a Schlenk flask. A solution of dimethyl malonyl chloride (0.295 mg, 1.75 mmol) in THF (80 mL) was added to the above solution at 0 ^o^C over the course of 2.0 h using a dropping funnel. The white suspension was stirred for 24 h at room temperature, followed by addition of H_2_O (60 mL). The THF was removed under reduced pressure to afford a white suspension, which was subsequently filtered. The residue was washed with H_2_O and Et_2_O, and dried under vacuum to afford the product as a white powder (0.510 g, 64 %). X-ray quality crystals of L^1^H_4_·3DMSO were obtained from concentrated DMSO solutions at room temperature. ^1^H NMR (400 MHz, (CD_3_)_2_SO): δ = 7.71 (d, *J* = 7.1 Hz, 2H), 7.11–7.28 (m, 12H), 4.39–4.48 (m, 2H), 3.37 (br, 2H) 2.92 (dd, *J* = 13.4 Hz, 7.4 Hz, 2H), 2.71 (dd, *J* = 13.4 Hz, 7.2 Hz, 2H), 1.64–1.73 (m, 2H), 1.54–1.64 (m, 2H), 1.14 (br, 10H). ^13^C NMR (101 MHz, (CD_3_)_2_SO): δ = 171.9, 170.1, 138.1, 129.4, 127.9, 126.1, 54.2, 52.6, 49.8, 36.9, 32.0, 24.4, 22.6. ATR-IR (KBr, cm^–1^): 3305, 2930, 2859, 1635, 1683, 1635, 1540. Elemental analysis (L^1^H_4_): Anal. Calcd for C_29_H_36_N_4_O_4_: C, 69.02; H, 7.19; N, 11.10. Found: C, 69.12; H, 7.21; N, 11.07.

### Synthesis of Cobalt­(III) Complexes

#### [K­(DMA)_2_Co^III^(L^1^)] (1)

Ligand L^1^H_4_ (0.246 g, 0.487 mmol) and potassium hydride (KH) (0.078 g, 1.94 mmol) were added to a 20 mL vial in a drybox. *N*,*N*-Dimethylacetamide (2.0 mL)^50^ was then added, and the mixture was stirred for 8 h affording a light-colored orange solution. Anhydrous CoCl_2_ beads (0.060 g, 0.463 mmol) were added to this solution, resulting in a brown-green colored mixture that was allowed to stir overnight. Dioxygen was then introduced in a Schlenk flask containing the mixture, turning its color from green to deep reddish purple. The mixture was then filtered to remove the solids, and the red-purple solution was layered with 40 mL of diethyl ether to afford red-purple crystals (0.222 g, 59%).^1^H NMR (400 MHz, CD_3_CN): δ = 10.9 (br, 2H), 9.89 (br, 2H), 4.36 (br, 4H), 2.93 (s, 6H), 2.82 (s,2H), 2.27–1.15 (m, 26H), -1.37 (br, 2H), −11.25 (br, 2H). ATR-IR (KBr, cm^–1^): 3278, 2929, 2863, 1626, 1574, 1393, 1013, 700, 590. UV–Vis (DMA): λ_max_ (ε (M^–1^ cm^–1^)): 517 nm (13135). Elemental analysis: Anal. Calcd for C_37_H_50_CoKN_6_O_6_: C, 57.50; H, 6.52; N, 10.87. Found: C, 57.57; H, 6.54; N, 10.84.

#### (PPh_4_)­[Co^III^(L^1^)] (2)

Under aerobic conditions, [K­(DMA)_2_Co^III^(L^1^)] (**1**) (0.290 g, 0.375 mmol) was dissolved in H_2_O (50 mL) and PPh_4_Cl (0.562 g, 1.5 mmol) in H_2_O (20 mL) was added while stirring. After 30 min, the purple suspension was filtered, and the residue was washed with H_2_O and pentane. The residue was dissolved in dichloromethane (50 mL) and dried over Na_2_SO_4_. The solution was filtered, and the filtrate was concentrated under reduced pressure to afford the product as a fine purple powder, which was dried under vacuum overnight. The solid was then dissolved in acetonitrile (2.0 mL), and the solution was layered with diethyl ether (40 mL) to afford reddish crystals upon standing at room temperature (0.242 g, 72 %). ^1^H NMR (400 MHz, CD_2_Cl_2_): δ = 11.1 (br, 2H), 9.87 (br, 2H), 7.67–7.53 (m, 8H), 7.18 (br, 8H), 7.01 (br, 4H), 5.10 (br, 4H), 2.60–1.10 (m, 20H), −1.01 (br, 2H), -11.23 (br, 2H). ATR-IR (KBr, cm^–1^) 3057, 2926, 2863, 2847, 1619, 1595, 1435, 1381, 1340, 1108, 721, 625. UV–Vis (MeCN): λ_max_ (ε (M^–1^ cm^–1^)): 517 nm (10979). Elemental analysis: Anal. Calcd for C_53_H_52_CoN_4_O_4_P: C, 70.82; H, 5.83; N, 6.23. Found: C, 70.89; H, 5.86; N, 6.20.

### Catalytic Studies

#### General Catalytic Olefin Aziridination Procedure

In a typical experiment conducted in a Drybox, a 20 mL screw-cap vial containing a small magnetic bar was charged in sequence with catalyst **1** or **2** (0.00625 mmol (2.5 mol %) or 0.0125 mmol (5.0 mol %)), *N*-(*p*-tolylsulfonyl)­imido-phenyliodinane (PhI=NTs) or *N*-(*p*-trifluoromethylbenzenesulfonyl)­imido-phenyliodinane (PhI=NSO_2_–C_6_H_4_-*p*-CF_3_)^8b^ (93.3 or 106.8 mg, 0.25 mmol) and molecular sieves (5 Å) (20 mg). Olefin (2.0 mmol) and solvent (0.300 mL) (as stated in the text) were charged in a separate vial. The solution of the olefin and the solvent was then added to the vial containing the catalyst, and the reaction mixture was stirred vigorously at 25 °C for 12 h (unless otherwise stated). After completion of the reaction, the products were purified by column chromatography (silica gel), first by eluting with hexane to remove the substrate and then with petroleum ether/ethyl acetate to elute products. Yields are quantified by ^1^H NMR (in CDCl_3_ or CD_3_CN) versus an internal standard (4-methoxyacetophenone). ^1^H- ^13^C- and ^19^F-NMR data of new compounds are summarized in the SI. All other aziridination and amination products (Tables 1–3) are known compounds and have been identified with the assistance of ^1^H and ^13^C NMR spectra, by comparison to spectroscopic features reported for authentic samples in the literature.^8b,55,67^

#### General Chemoselective Reaction Procedure Involving Competitive Aziridination of Olefins

For the competitive aziridinations of styrene by PhI=NTs or PhI=NSO_2_–C_6_H_4_-*p*-CF_3_ versus 1-hexene, allylbenzene and 4-phenyl-1-butene (Table 3), mediated by catalyst **2** (2.5 mol %) in PhCF_3_, the general aziridination procedure noted above was followed, with the exception that 2.0 mmol of each olefin was used.

### Mechanistic Studies

#### Catalytic Reaction Procedure for Hammett Analysis

For each competition experiment, a 20 mL screw-cap vial containing a small magnetic bar was charged in sequence with catalyst **2** (5.6 mg, 0.00625 mmol, 2.5 mol %), *N*-(*p*-tolylsulfonyl)­imido-phenyliodinane (PhI=NTs) or N-(*p*-trifluoromethyl-benzenesulfonyl)­imido-phenyliodinane (PhI=NSO_2_–C_6_H_4_-*p*-CF_3_) (93.3 or 106.8 mg, 0.25 mmol) and molecular sieves (5 Å) (20 mg). Styrene (2.0 mmol), 4-X-styrene (2.0 mmol, X = ^*t*^Bu, Me, F, Cl, Br, CN, CF_3_, NO_2_), and α,α,α-trifluorotoluene (0.300 mL) were charged in another vial. The olefinic solution was then added to the vial containing the solids, and the reaction mixture was stirred vigorously at 25 °C for 2.5 h. At the end of the reaction, the mixture was flash chromatographed on silica gel (hexane, then petroleum ether/ethyl acetate 8:1 v/v; petroleum ether/ethyl acetate 4:1 v/v for 4-X-styrene (X = CN, CF_3_, NO_2_)) in order to recover the aziridination products and evaluate the ratio *k*_X_/*k*_H_ (Table S3) by quantitative ^1^H NMR analysis in CD_3_CN (relaxation delay: 120 s).

#### Competitive Aziridinations of Deuterated Styrenes versus Styrene (KIE)

In order to determine secondary deuterium kinetic isotope effect values (*k*_H_/*k*_D_), a suitably deuterated styrene (α-d_1_-styrene or *cis*-β-d_1_-styrene) was used together with styrene (2.0 mmol each) and was subjected to aziridination by *N*-(*p*-tolylsulfonyl)­imido-phenyliodinane (PhI=NTs) or *N*-(*p*-trifluoromethylbenzenesulfonyl)­imido-phenyliodinane (PhI=NSO_2_–C_6_H_4_-*p*-CF_3_) (93.3 or 106.8 mg, 0.25 mmol) in the presence of catalyst **2** (2.5 mol %) and molecular sieves (5 Å, 20 mg) in α,α,α-trifluorotoluene (0.30 mL), according to the general olefin aziridination procedure noted above. Prior to mixing with any other reagents, a drop of the original mixture of deuterio and protio styrene was retained for ^1^H NMR analysis (CDCl_3_, 150 s relaxation delay) to determine the starting d_0_/d_1_ styrene ratio. The reaction was quenched after 0.5 h, and the mixture was flash chromatographed on silica gel, first with hexane to remove the styrenes and then with petroleum ether/ethyl acetate (5:1 v/v) to recover the aziridination products. The ratio of d_0_/d_1_ aziridines is evaluated by ^1^H NMR (CD_3_CN, 120 s relaxation delay) and compared to the ratio of the starting d_0_/d_1_ styrenes in order to extract secondary KIE values.

#### Stereochemical Scrambling in the Aziridination of *cis*-β-d_1_-Styrene

The aziridination of *cis*-β-d_1_-styrene versus styrene, mediated by catalyst **2** (2.5 mol %), was conducted according to the general procedure noted in the previous section. In addition, the aziridination of *cis*-β-d_1_-styrene, as a sole substrate, was executed under the same conditions. In both cases, the isolated aziridines were dissolved in CHCl_3_ (700 μL)/CDCl_3_ (20 μL) and loaded in a precision NMR tube. Extensive ^2^H NMR data were collected (relaxation delay: 15 s) for three independent reaction trials, in order to quantify the relative deuterium content at the *cis* and *trans* β-carbon sites of the aziridination products and extract *cis*/*trans* aziridination ratios.

### Stoichiometric Reactions of Catalyst 2 with Nitrene Precursors

#### UV–Vis Studies

A 3.7 × 10^–4^ M solution of (PPh_4_)­[Co^III^(L^1^)] (2) in CH_3_CN was prepared in a dinitrogen-filled glovebox. Various equivalents (2–12 equiv) of the nitrene precursor (PhI=NTs or PhI=NSO_2_–C_6_H_4_-*p*-CF_3_) were weighed in a separate vial, charged with a stir bar. A 10 mL aliquot of the aforementioned solution of the Co­(III) complex 2 was added to the vial containing the nitrene precursor, and the mixture was allowed to stir for 15 min. An 1.0 mL aliquot of this reaction mixture was taken and diluted to a final volume of 3 mL to obtain UV–Vis spectra in a dinitrogen-filled 1.0 cm quartz cuvette that was covered with a Teflon screw-cap.

#### ESI-LCMS Studies

(PPh_4_)­[Co^III^(L^1^)] (**2**) (3.0 μmol) was allowed to react with PhI=NTs or PhI=NSO_2_–C_6_H_4_-*p*-CF_3_ (2, 5, and 10 equiv) in MeCN for 10 min under ambient conditions, and the mixture, after removal of any remaining PhI=NR solid, was analyzed by ESI-MS. Negative mode electrospray ionization liquid chromatography–mass spectrometry (ESI-LCMS) analysis was performed using an Agilent InfinityLab 1290 II UHPLC/ESI-MS system and/or a Shimadzu 9030 q-tof high-resolution LC-MS. Samples were analyzed in negative-ion mode using HPLC grade acetonitrile as solvent.

## Supplementary Material



## Notes

X-ray crystallographic details, additional procedures for physical measurements (magnetic susceptibility, electrochemistry), additional mechanistic data (Hammett analysis, UV–vis and ESI-MS spectra), ^1^H, ^13^C, ^19^F NMR data (PDF)
