# Supplementary material for: Nitrene-Transfer Chemistry to Olefins Mediated by Co(III) Catalysts Supported by a Tetraamido Macrocycle
Source: Inorg Chem. 2026 Sep 13;65(40):23487–504. doi: 10.1021/acs.inorgchem.6c02795 (PMC13664075; doi:10.1021/acs.inorgchem.6c02795)
Supplement: Supplementary file 1 [file ic6c02795_si_001.pdf]

Supporting Information

Nitrene-Transfer Chemistry to Olefins Mediated by Co(III) Catalysts Supported by a Tetraamido Macrocycle

Himanshu Bhatia, Jacob Green, Mia Ehrhart, Rohit G. Jadhav, Amitava Choudhury, and Pericles Stavropoulos*

Department of Chemistry, Missouri University of Science and Technology, Rolla, Missouri 65409, United States

* To whom correspondence should be addressed. Tel.: (+1) 573-341-7220, Fax: (+1) 573-341-6033. E-mail: pericles@mst.edu

Table of Contents

1. Physical Measurements S3

   X-ray crystallography S3

   Magnetic Susceptibility Measurements by the Evans ^{1}H-NMR Method. S5

   Electrochemistry S6

2. Catalytic and Mechanistic Data S7

3. Stoichiometric Reactions of Catalyst **2** with Nitrene Precursors S10

4. NMR Data for New Products S12

5. References S40

Physical Measurements

X-ray Crystallography. Intensity data sets for all compounds were collected on either a Rigaku XtaLAB Synergy or a Bruker Apex II diffractometer using graphite monochromated Mo or Cu (λ_{Mo} = 0.71073 and λ_{Cu} = 1.54178 Å) Kα radiation from a fine focus or microfocus sealed X-ray tube. Suitable crystals were selected and mounted on a glass fiber using super glue. The datasets were collected at low temperatures ranging between 100 to 173 K, employing a scan of 0.3° in ω with an exposure time of 10 s/frame using Apex III or CrysAlisPro software.[1,2] The unit cell refinement and data reduction were carried out with SAINT or CrysAlisPro, while the program SADABS was used for the absorption correction.[1,2] The structures were solved by direct methods using SHELXS-97 and difference Fourier syntheses.[3] Full-matrix least-squares refinement against $|F^2|$ was carried out using the SHELXTL-PLUS[1] suite of programs. All non-hydrogen atoms were refined anisotropically. Hydrogen atoms were placed geometrically and held in the riding mode during the final refinement. All structures were finally refined with SHELX-2014 using SHELXle.[4] The overall data quality of ligand L^1H_4 was poor due to thin and needle-shaped crystals, resulting in B-level alerts. The noisy data beyond 2-theta = 48 were eliminated, which lowered the sine(theta_max)/wavelength ratio. However, this action improved the ratio of Observed/Unique reflections and refinement parameters. Explanations for the B-level alerts are provided in the validation response form (VRF) embedded in the cif files. Unit cell parameters and pertinent crystallographic details are listed in Table S1. ORTEP-type diagrams and selective metrical parameters for all compounds are shown in Figures 2 and S1.

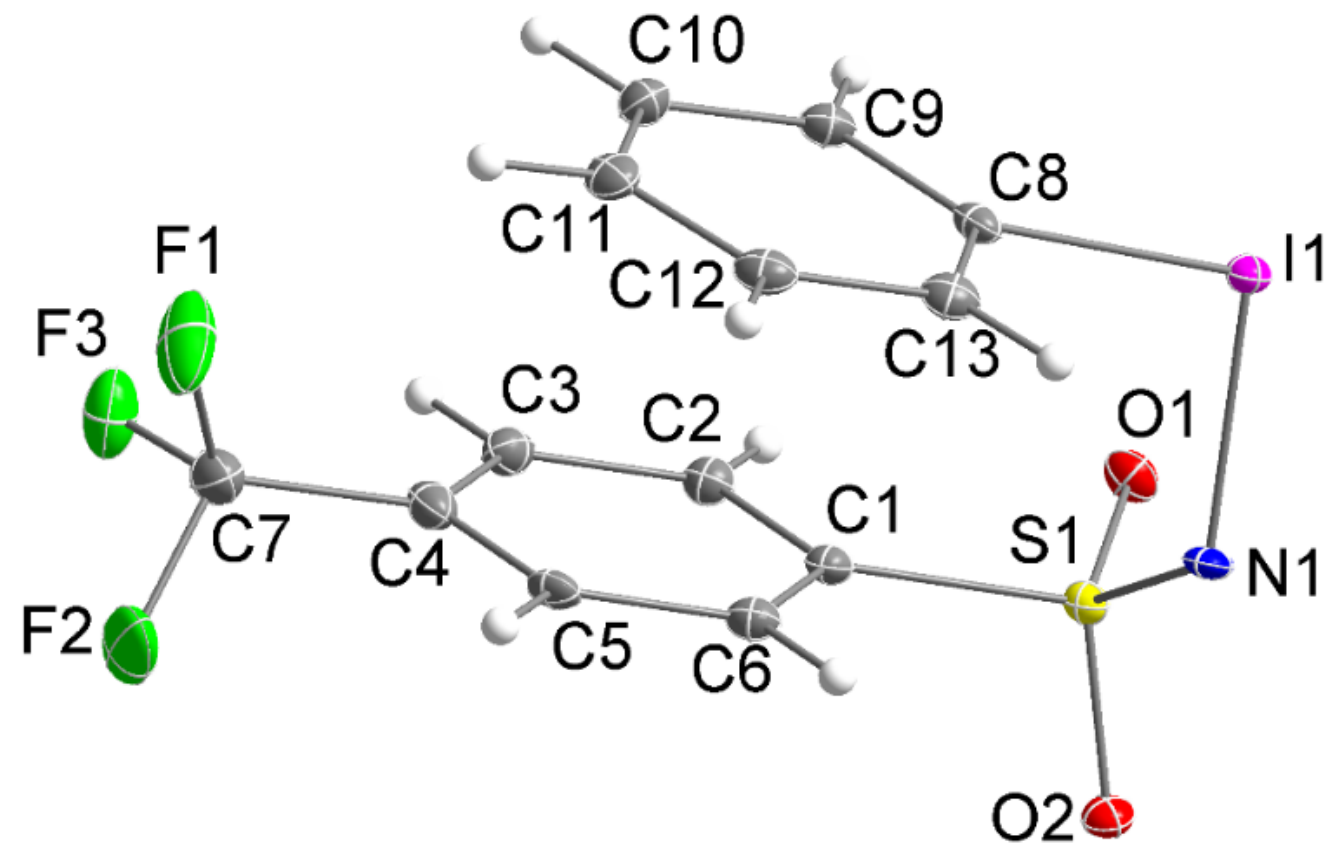

Figure S1. ORTEP diagram of PhI=NSO_2-C_6H_4-*p*-CF_3 drawn with 40% thermal ellipsoids. Selective interatomic distances (Å) and angles (°) for PhI=NSO_2-C_6H_4-*p*-CF_3 : I(1)–N(1) = 2.007(3), I(1)–C(8) = 2.099(3), S(1)–O(1) = 1.439(3), S(1)–O(2) = 1.460(2), S(1)–N(1) = 1.595(3), S(1)–C(1) = 1.785(4), N(1)–I(1)–C(8) = 97.86(12), S(1)–N(1)–I(1) = 113.87(16).

Table S1. Summary of Crystallographic Data for Ligand L^1H_4•3DMSO, [K(DMA)$_2$Co^{III}(L^1)], (PPh_4)[Co^{III}(L^1)] and PhI=NSO_2-C_6H_4-*p*-CF_3

	**L^1H_4•3DMSO**	**[K(DMA)$_2$Co^{III}(L^1)]**	**(PPh_4)[Co^{III}(L^1)]**	**PhI=NSO_2-C_6H_4-*p*-CF_3**
formula	$C_{70}H_{108}N_8O_{14}S_6$	$C_{74}H_{100}Co_2K_2N_{12}O_{12}$	$C_{53}H_{52}CoN_4O_4P$	$C_{13}H_9F_3INO_2S$
M_r	1478.00	1545.71	898.88	427.17
crystal system	Monoclinic	Orthorhombic	Tetragonal	Monoclinic
space group	$P2_1$	$P2_12_12$	$P4_3$	$P2_1/c$
a (Å)	17.6918(11)	36.469(9)	11.0102(3)	15.3372(3)
b (Å)	9.9501(7)	16.262(4)	11.0102(3)	6.42700(10)
c (Å)	21.8872(14)	12.756(3)	38.2002(16)	14.0010(2)
α (deg)	90	90	90	90
β (deg)	97.432(4)	90	90	96.334(2)
γ (deg)	90	90	90	90

V (Å^3)	3820.5(4)	7565(3)	4630.8(3)	1371.68(4)
Z	2	4	4	4
D_{calcd} (g cm^{-3})	1.285	1.356	1.289	2.069
T (K)	150(2)	150(2)	173(2)	100(2)
λ (Å)	1.54178	0.71073	0.71073	1.54178
μ (mm^{-1})	2.189	0.616	0.455	20.156
$R_1{}^a$ (I>2sigma(I))	0.0675	0.0477	0.0344	0.0391
$wR_2{}^b$ (I>2sigma(I))	0.1734	0.1073	0.0647	0.1101

^{a} $R_1 = \Sigma||Fo| - |Fc||/\Sigma|Fo|$. ^{b} $wR_2 = [\Sigma w(Fo^2 - Fc^2)^2/\Sigma w(Fo^2)^2]^{1/2}$

Magnetic Susceptibility Measurements by the Evans ^{1}H-NMR Method. A solvent mixture containing 0.2 mL CD_3CN, 4.0 mL CH_3CN and 1-2 drops of tetramethylsilane (TMS) was prepared and used to charge a tip-sealed glass pipette (2 inches of bubble-free liquid), followed by flame-sealing of the top narrow end of the pipette, to generate a thin capillary insert. The same solvent mixture (1.2 mL) was used to fully dissolve 38 mg of paramagnetic compound **2**, accurately weighted on an analytical balance before and after adding the solvent. The capillary insert was then transferred in a J Young 5mm NMR tube, followed by the solution of the compound, and a standard ^{1}H NMR spectrum was recorded. The temperature of the probe and the operating instrument radiofrequency (400 MHz) were noted. The effective magnetic moment was calculated according to literature procedures,[5] by evaluating the peak shifts (Δppm) of CH_3CN and TMS of the sample versus those of the reference (CH_3CN and TMS in the capillary). Those were converted to hertz (Hz) by using the equation: Δf (Hz) = ΔppmF/106, where F is the spectrometer's radiofrequency in Hz. Then, the molar magnetic susceptibility (XM) is obtained via the equation: XM = 3Δf /4πFc, where c is the concentration of the paramagnetic species (mol/mL). The magnetic moment (μ) is then calculated from the following equation: μ = [8(XMT)]$^{1/2}$, where XM is the

molar magnetic susceptibility (cm^3/mol), T is the temperature (K) of the NMR probe and μ is the μ_{obs} (B.M.)

Electrochemistry. Electrochemical measurements were performed under moisture- and oxygen-free conditions inside an Ar-filled glovebox using a BioLogic VSP-300 potentiostat. All electrochemical experiments were carried out using dry, degassed solvent and supporting electrolyte prepared and handled under an inert atmosphere to minimize interference from residual water and oxygen. Cyclic voltammetry was used to evaluate the redox behavior of the cobalt complex, and scan-rate-dependent measurements were recorded to assess the reversibility and transport characteristics of the redox couple using glassy carbon electrode as working electrode, Ag/Ag^+ electrode as reference electrode and Pt as counter electrode. The cathodic and anodic peak potentials were extracted from the corresponding reduction and oxidation waves, and the peak-to-peak separation ($\Delta E_p = E_{ox} - E_{red}$), i_{pa}/i_{pc} ratio and proportionality of cathodic and anodic currents versus the square root of the scan rate (Figure S2) were used to evaluate electrochemical reversibility.

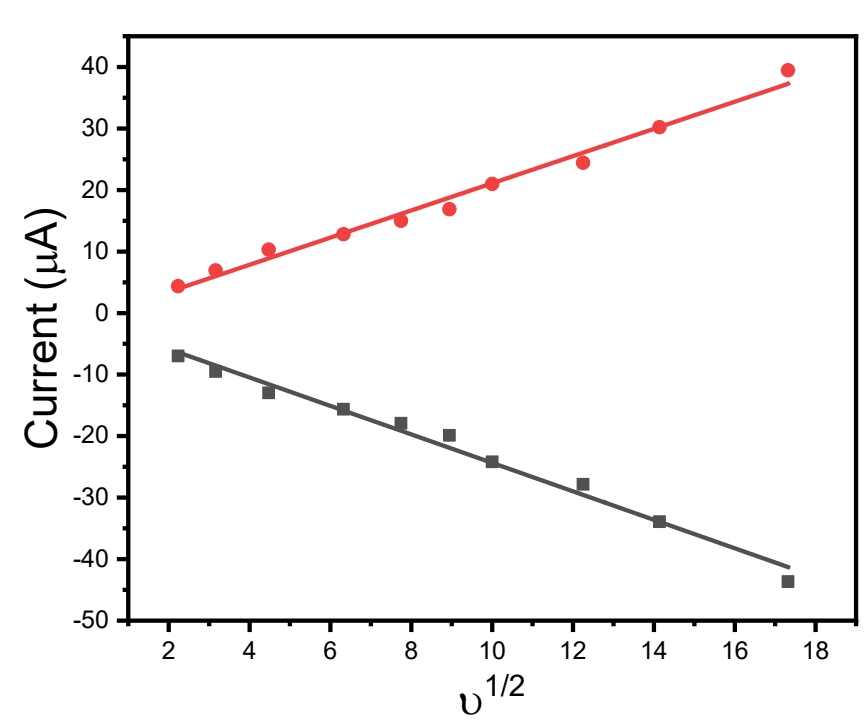

Figure S2. Plot of cathodic and anodic peak current versus the square root of scan rate for the Co(II)/Co(III) redox couple of complex $(PPh_4)[Co^{III}(L^1)]$ (**2**).

Catalytic and Mechanistic Data

Table S2. Yields of Styrene Aziridination Mediated by Catalysts **1** and **2**[a]

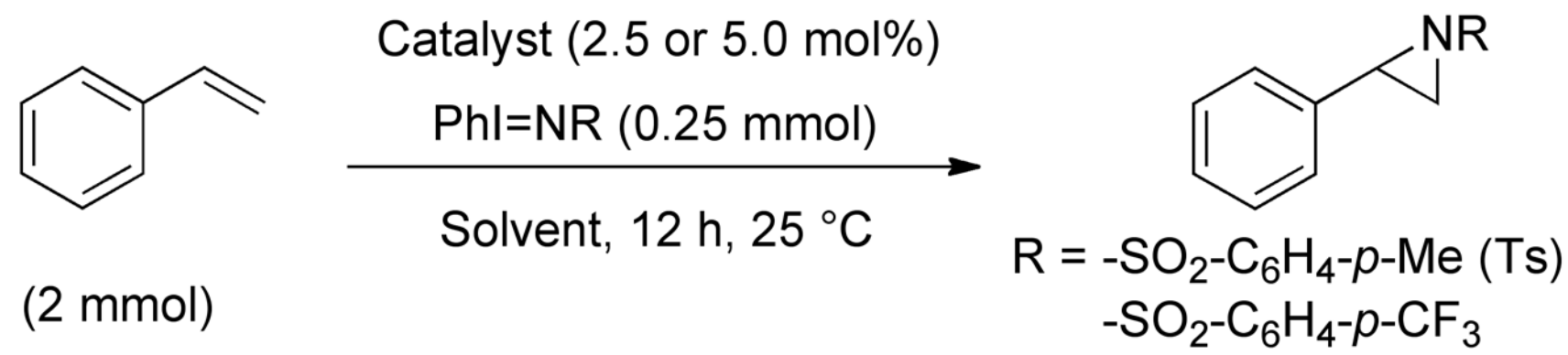

Entry No.	Catalyst (mol%)	PhI=NR	Solvent	Yield (%)
1.	**1** (2.5 mol%)	PhI=NTs	MeCN	55
2.	**1** (2.5 mol%)	PhI=NTs	1,2-DCE[b]	59
3.	**1** (2.5 mol%)	PhI=NTs	DCM	55
4.	**1** (2.5 mol%)	PhI=NTs	PhCl	58
5.	**1** (2.5 mol%)	PhI=NTs	$PhCF_3$	62
6.	**1** (5.0 mol%)	PhI=NTs	$PhCF_3$	62
7.	**2** (2.5 mol%)	PhI=NTs	MeCN	63
8.	**2** (2.5 mol%)	PhI=NTs	1,2-DCE	60
9.	**2** (2.5 mol%)	PhI=NTs	DCM	63
10.	**2** (2.5 mol%)	PhI=NTs	PhCl	63
11.	**2** (2.5 mol%)	PhI=NTs	$PhCF_3$	64
12.	**2** (5.0 mol%)	PhI=NTs	$PhCF_3$	63
13.	**2** (2.5 mol%)	$PhI=NSO_2-C_6H_4-$ *p*-CF_3	MeCN	67
14.	**2** (2.5 mol%)	$PhI=NSO_2-C_6H_4-$ *p*-CF_3	1,2-DCE	74
15.	**2** (2.5 mol%)	$PhI=NSO_2-C_6H_4-$ *p*-CF_3	DCM	70
16.	**2** (2.5 mol%)	$PhI=NSO_2-C_6H_4-$ *p*-CF_3	PhCl	73
17.	**2** (2.5 mol%)	$PhI=NSO_2-C_6H_4-$ *p*-CF_3	1,2-DFB[c]	69
18.	**2** (2.5 mol%)	$PhI=NSO_2-C_6H_4-$ *p*-CF_3	$PhCF_3$	**74**

19.	**2** (5.0 mol%)	$PhI=NSO_2-C_6H_4-$*p*$-CF_3$	$PhCF_3$	**79**
20.	**2** (2.0 mol%)	$PhI=NSO_2-C_6H_4-$*p*$-CF_3$	$PhCF_3$	62

[a]Co(III) catalyst **1** or **2**, 0.00625 (2.5 mol%) or 0.0125 mmol (5.0 mol%); PhI=NR, 0.25 mmol; styrene, 2.0 mmol; MS 5Å, 20 mg; solvent, 0.30 mL; 25 °C; 12 h; NMR yields. [b]1,2-DCE = 1,2-dichloroethane. [c]1,2-DFB = 1,2-difluorobenzene.

Hammett Analysis

Table S3. Competitive Aziridination Reactions of 4-X-Styrenes *vs.* Styrene by PhI=NTs or $PhI=NSO_2-C_6H_4-$*p*$-CF_3$ Mediated by Catalyst **2**[a]

**X**	σ_p	σ^+	σ_{mb}	$\sigma_{JJ}^{\bullet}$	**PhINTs** k_X/k_H	$PhI=NSO_2-C_6H_4-$***p***$-CF_3$ k_X/k_H
t-Bu	-0.197	-0.26	-0.22	0.26	1.7642	1.5454
Me	-0.17	-0.31	-0.29	0.15	1.9099	1.774
H	0	0	0	0	1	1
F	0.062	-0.07	-0.24	-0.02	1.7643	1.1238
Cl	0.227	0.11	0.11	0.22	1.7666	1.0728
Br	0.23	0.11	0.13	0.23	1.7588	0.9808
CF_3	0.54	0.61	0.49	-0.01	0.8665	0.4317
CN	0.66	0.659	0.86	0.42	0.9344	0.4264
NO_2	0.778	0.79	0.86	0.36	0.7256	0.3611

[a]Reaction conditions: catalyst **2**, 0.00625 mmol (2.5 mol%); 4-X-styrene, 2.0 mmol; styrene, 2.0 mmol; PhI=NTs or $PhI=NSO_2-C_6H_4-$*p*$-CF_3$ 0.125 mmol; $PhCF_3$, 0.300 mL; MS 5 Å, 20 mg; T = 25 °C; *t* = 2.5 h.

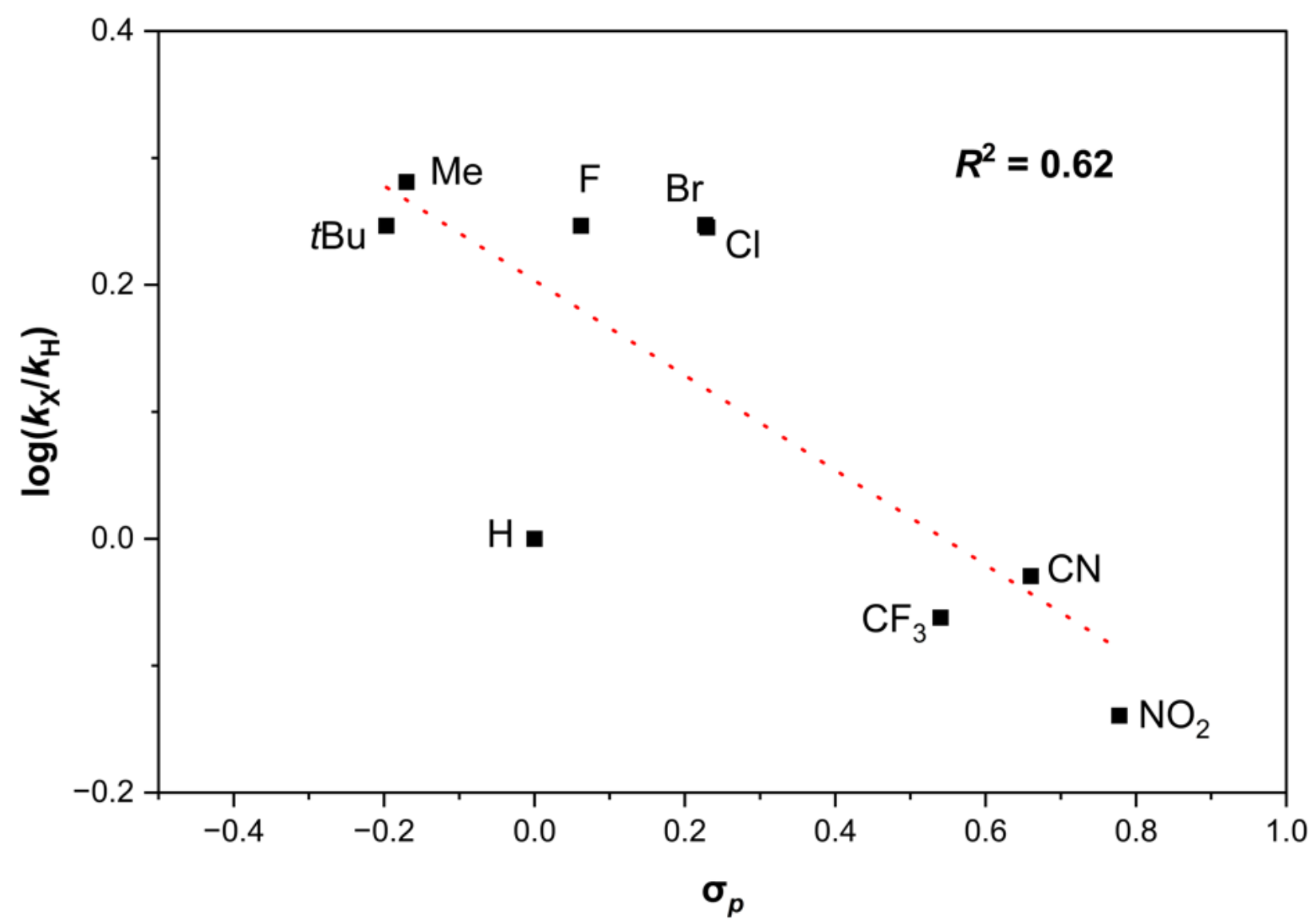

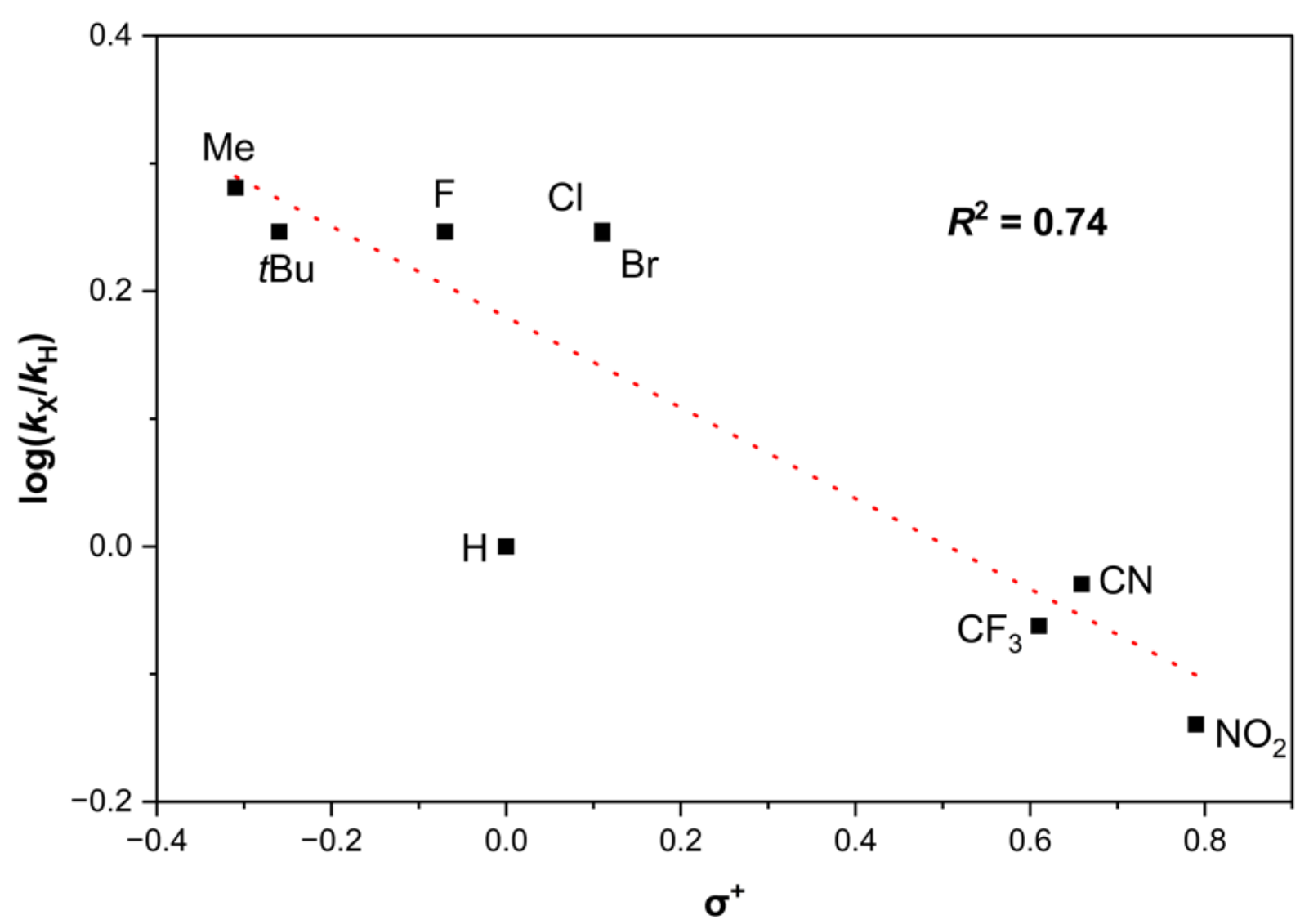

Figure S3. Linear free energy correlation of $\log(k_X/k_H)$ as a function of σ_p (top) and σ^+ (bottom) for the competitive aziridination of 4-X-styrene vs styrene with PhI=NTs catalyzed by $[(PPh_4)][Co^{III}(L^1)]$ (**2**).

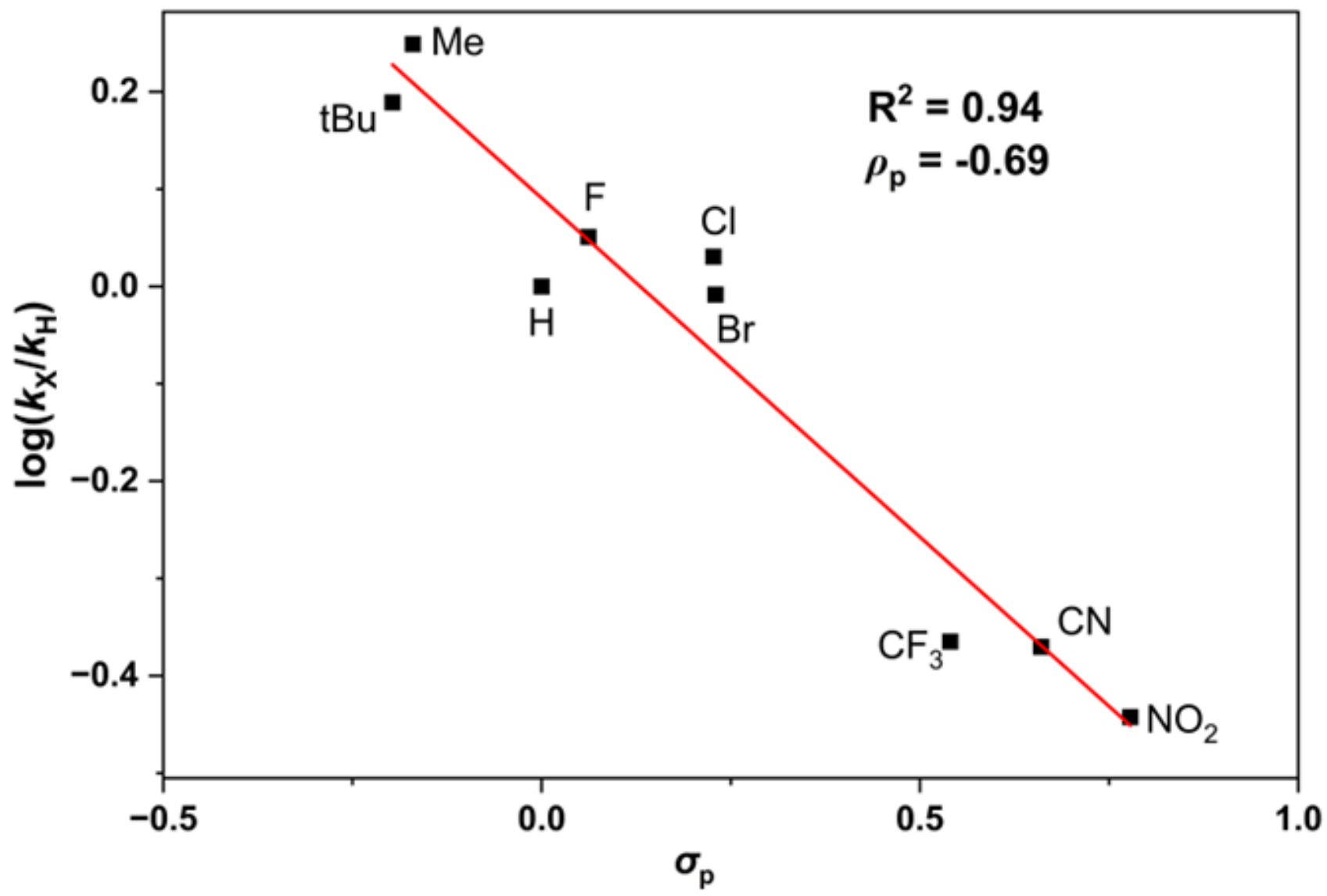

Figure S4. Linear free energy correlation of $\log(k_X/k_H)$ as a function of σ_p for the competitive aziridination of 4-X-styrene vs styrene with PhI=NSO_2-C_6H_4-*p*-CF_3 catalyzed by [(PPh_4)][$Co^{III}(L^1)$] (**2**).

Stoichiometric Reactions of Catalyst (PPh_4)[$Co^{III}(L^1)$] (2) with Nitrene Precursors:

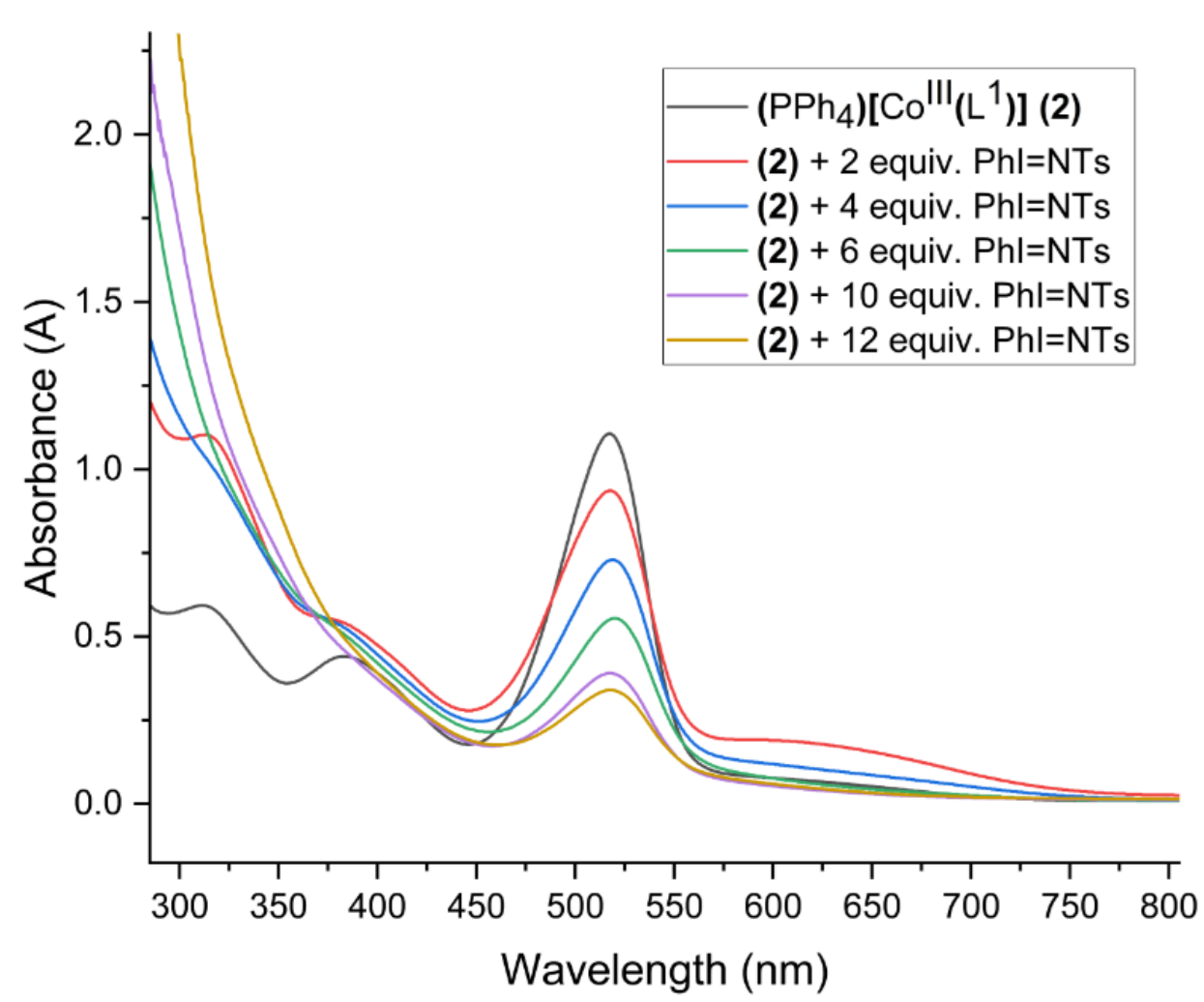

Figure S5. UV-Vis spectra of **2** upon addition of various equivalents of PhI=NTs in MeCN.

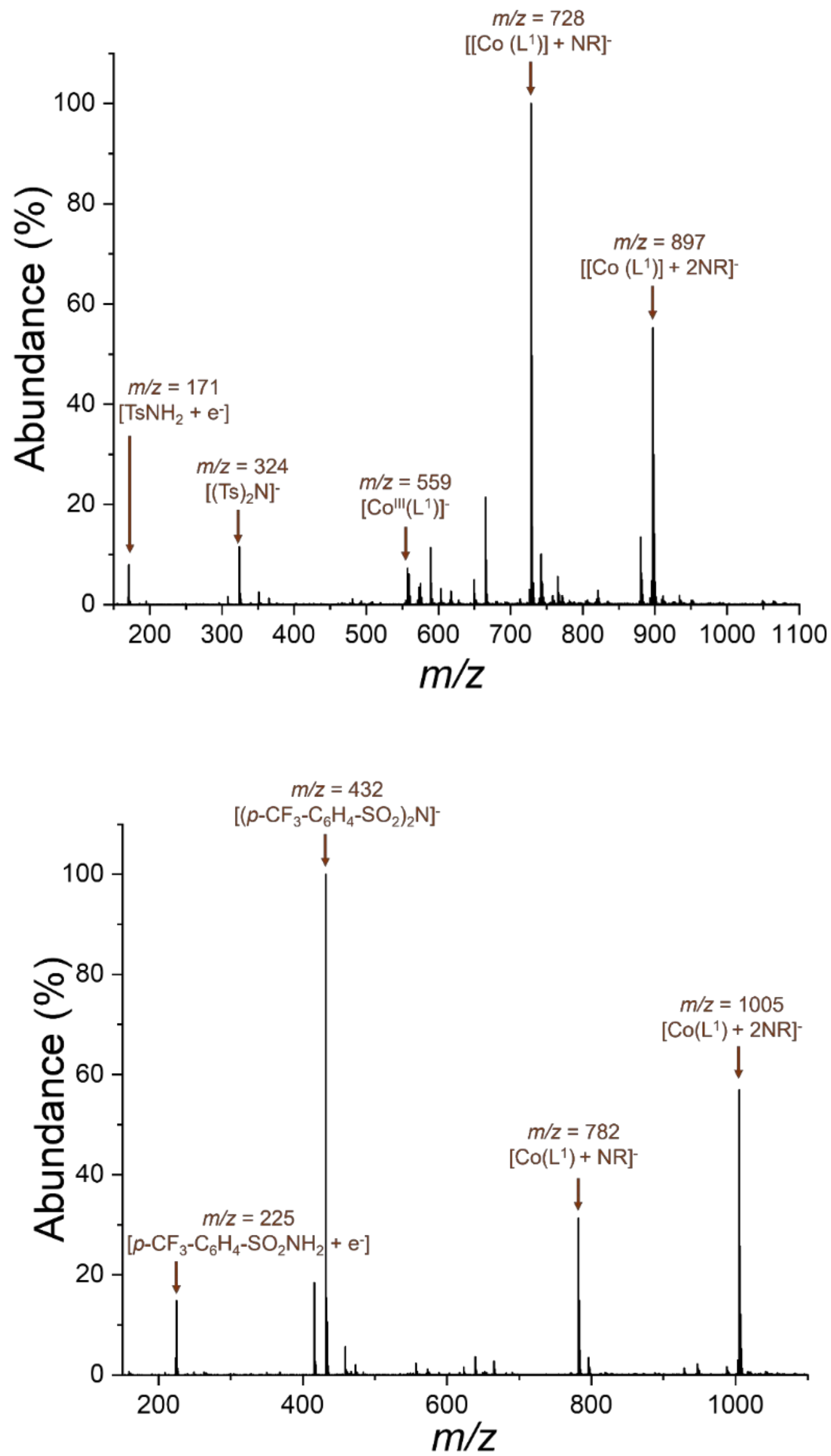

Figures S6. ESI-MS spectra of $(PPh_4)[Co^{III}(L^1)]$ (**2**) after addition of 10 equiv. of PhI=NTs (top) or PhI=NSO_2-C_6H_4-*p*-CF_3 (bottom) in MeCN (10 min).

NMR Data for New Products of Catalytic Reactions

2-(p-Tolyl)-1-((4-(trifluoromethyl)phenyl)sulfonyl)aziridine:

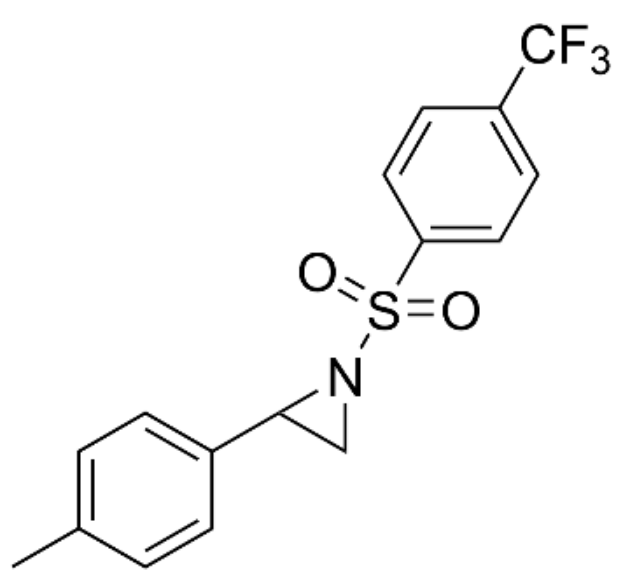

^{1}H NMR (400 MHz, $CDCl_3$): δ = 8.12 (d, J = 8.2 Hz, 2H), 7.80 (d, J = 8.2 Hz, 2H), 7.15 – 7.08 (m, 4H), 3.85 (dd, J = 7.2 Hz, J = 4.6 Hz, 1H), 3.06 (d, J = 7.2 Hz, 1 H), 2.47 (d, J = 4.6 Hz, 1H), 2.32 (s, 3H). **^{13}C NMR** (101 MHz, $CDCl_3$): δ = 142.0, 138.7, 135.3 (q, J = 32.9 Hz), 131.5, 129.5 128.5, 126.5, 126.4 (q, J = 3.7 Hz), 123.3 (q, J = 271.3 Hz), 41.8, 36.4, 21.3. **^{19}F NMR** (376 MHz, $CDCl_3$): δ = -63.2.

2-(4-(Tert-butyl)phenyl)-1-((4-(trifluoromethyl)phenyl)sulfonyl)aziridine:

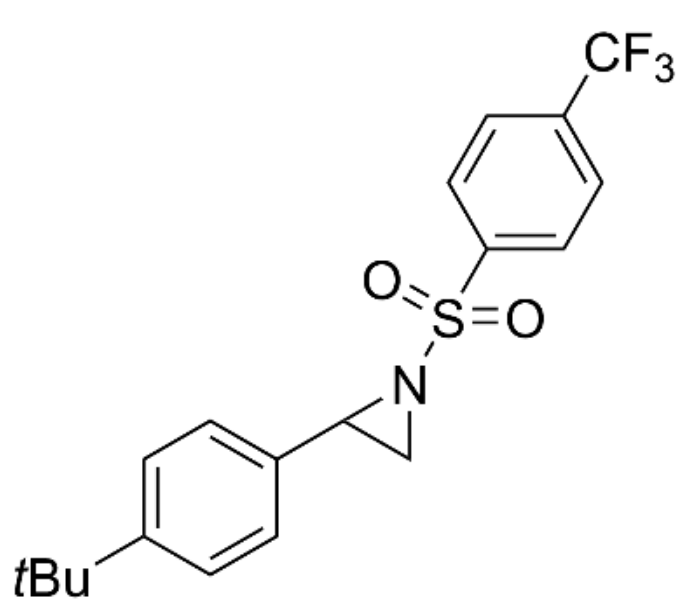

^{1}H NMR (400 MHz, $CDCl_3$): δ = 8.13 (d, J = 8.2 Hz, 2H), 7.81 (d, J = 8.2 Hz, 2H), 7.37 – 7.32 (m, 2H), 7.19 – 7.13 (m, 2H), 3.88 (dd, J = 6.1 Hz, J = 4.6 Hz, 1H), 3.06 (d, J = 7.2 Hz, 1 H), 2.47 (d, J = 4.6 Hz, 1H), 1.29 (s, 9H). **^{13}C NMR** (101 MHz, $CDCl_3$): δ = 152.0, 142.0, 138.7, 135.4 (q,

J = 32.0 Hz), 131.5, 128.6, 126.4 (q, *J* = 3.5 Hz), 126.4, 125.8, 123.3 (q, *J* = 271.4 Hz), 41.7, 36.4, 34.8, 31.4. **^{19}F NMR** (376 MHz, $CDCl_3$): δ = -63.2.

2-(4-Chlorophenyl)-1-((4-(trifluoromethyl)phenyl)sulfonyl)aziridine:

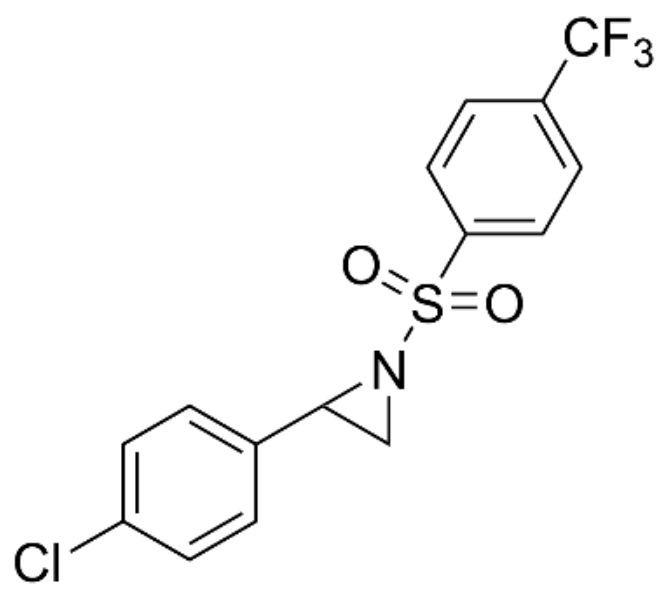

^{1}H NMR (400 MHz, $CDCl_3$): δ = 8.12 (d, *J* = 8.2 Hz, 2H), 7.82 (d, *J* = 8.2 Hz, 2H), 7.31 – 7.26 (m, 2H), 7.19 – 7.14 (m, 2H) 3.85 (dd, *J* = 7.2 Hz, *J* = 4.5 Hz, 1H), 3.06 (d, *J* = 7.2 Hz, 1 H), 2.42 (d, *J* = 4.6 Hz, 1H). **^{13}C NMR** (101 MHz, $CDCl_3$): δ = 141.8, 135.6 (q, *J* = 33.0 Hz), 134.7, 133.2, 129.1 128.6, 128.0 126.5 (q, *J* = 3.7 Hz), 123.2 (q, *J* = 271.4 Hz), 40.9, 36.7. **^{19}F NMR** (376 MHz, $CDCl_3$): δ = -63.2.

2-(4-Fluorophenyl)-1-((4-(trifluoromethyl)phenyl)sulfonyl)aziridine:

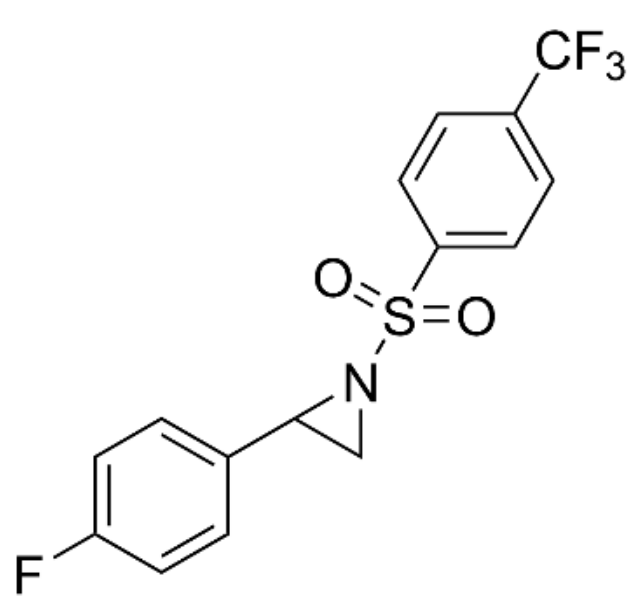

^{1}H NMR (400 MHz, $CDCl_3$): δ = 8.12 (d, *J* = 8.2 Hz, 2H), 7.82 (d, *J* = 8.0 Hz, 2H), 7.23 – 7.17 (m, 2H), 7.04 – 7.96 (m, 2H) 3.86 (dd, *J* = 7.2 Hz, *J* = 4.6 Hz, 1H), 3.05 (d, *J* = 7.2 Hz, 1 H), 2.43

(d, *J* = 4.6 Hz, 1H). [13]**C NMR** (101 MHz, $CDCl_3$): δ = 163.0 (d, *J*= 246.3 Hz), 141.8, 135.5 (q, *J* = 33.2 Hz), 130.4 (d, *J* =3.3 Hz), 128.6, 128.4 (d, *J* = 8.2 Hz) 126.5 (q, *J* = 3.6 Hz), 123.2 (q, *J* = 271.0 Hz), 115.9 (d, *J* = 21.6), 41.0, 36.6. [19]**F NMR** (376 MHz, $CDCl_3$): δ = -63.2, -113.8.

2-(4-(Trifluoromethyl)phenyl)-1-((4-(trifluoromethyl)phenyl)sulfonyl)aziridine:

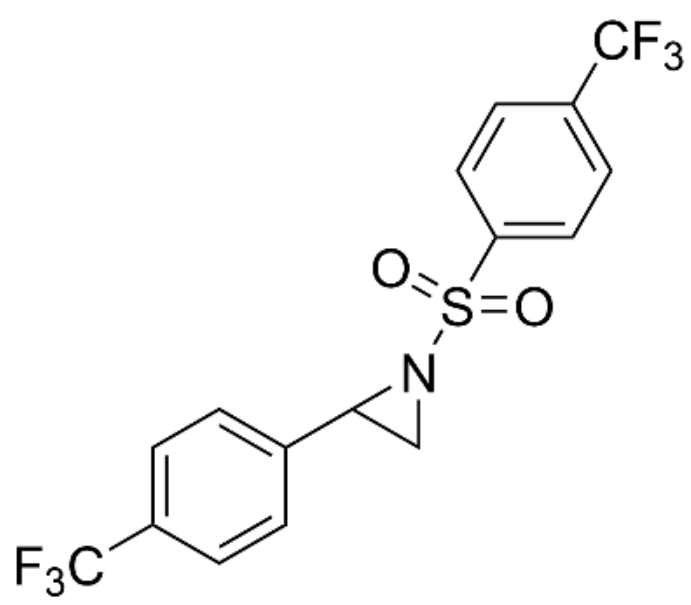

[1]**H NMR** (400 MHz, $CDCl_3$): δ = 8.13 (d, *J* = 8.2, 2H), 7.83 (d, *J* = 8.2 Hz, 2H), 7.58 (d, *J* = 8.2 Hz, 2H), 7.36 (d, *J* = 8.2 Hz, 2H), 3.92 (dd, *J* = 7.2 Hz, *J* = 4.4 Hz, 1H), 3.10 (d, *J* = 7.2 Hz, 1 H), 2.45 (d, *J* = 4.4 Hz, 1H). [13]**C NMR** (101 MHz, $CDCl_3$): δ = 141.6, 138.8 136.2 (q, *J* = 33.2 Hz), 131.0 (q, *J* = 32.7 Hz), 128.6, 127.0 126.6 (q, *J* = 3.7 Hz), 125.9 (q, *J* = 3.9 Hz) 124.0 (q, *J* = 270.6 Hz), 123.2 (q, *J* = 271.5 Hz), 40.7, 36.8. [19]**F NMR** (376 MHz, $CDCl_3$): δ = -63.3, -62.8.

2-(4-Nitrophenyl)-1-((4-(trifluoromethyl)phenyl)sulfonyl)aziridine:

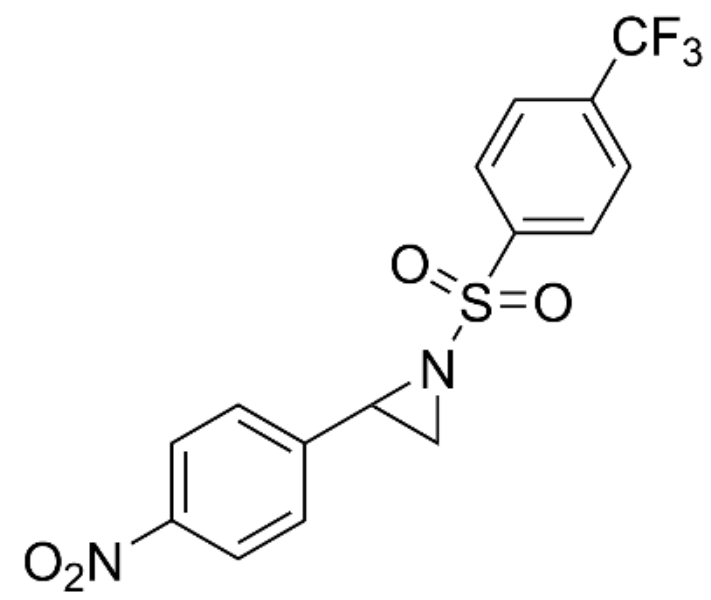

^{1}H NMR (400 MHz, $CDCl_3$): δ = 8.20 – 8.15 (m, 2H), 8.14 (d, *J* = 8.2 Hz, 2H), 7.84 (d, *J* = 8.3 Hz, 2H), 7.45 – 7.40 (m, 2H), 3.96 (dd, *J* = 7.2 Hz, *J* = 4.4 Hz, 1H), 3.13 (d, *J* = 7.2 Hz, 1 H), 2.45 (d, *J* = 4.4 Hz, 1H). **^{13}C NMR** (101 MHz, $CDCl_3$): δ 141.8, 135.6 (q, *J* = 33.0 Hz), 134.7, 133.2, 129.1 128.6, 128.0 126.5 (q, *J* = 3.7 Hz), 123.2 (q, *J* = 271.4 Hz), 40.9, 36.7. **^{19}F NMR** (376 MHz, $CDCl_3$): δ = -63.2.

***cis*-2-Methyl-3-phenyl-1-((4-(trifluoromethyl)phenyl)sulfonyl)aziridine:**

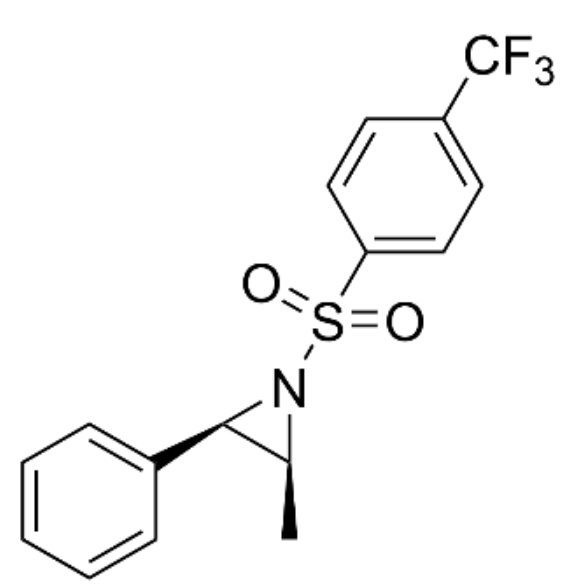

^{1}H NMR (400 MHz, $CDCl_3$): δ = 8.15 (d, *J* = 8.2 Hz, 2H), 7.82 (d, *J* = 8.3 Hz, 2H), 7.34 – 7.25 (m, 3H), 7.23 – 7.17 (m, 2H), 4.04 (d, *J* = 7.3, 1H), 3.33 – 3.24 (m, 1 H), 1.05 (d, *J* = 5.8 Hz, 3H). **^{13}C NMR** (101 MHz, $CDCl_3$): δ = 142.3, 135.3 (q, *J* = 33.0 Hz), 132.3, 128.6, 128.4, 128.3, 127.6, 126.4 (q, *J* = 3.7 Hz), 123.3 (q, *J* = 271.4 Hz), 46.8, 42.5, 12.0. **^{19}F NMR** (376 MHz, $CDCl_3$): δ = -63.2.

***cis*-2,3-Diphenyl-1-((4-(trifluoromethyl)phenyl)sulfonyl)aziridine:**

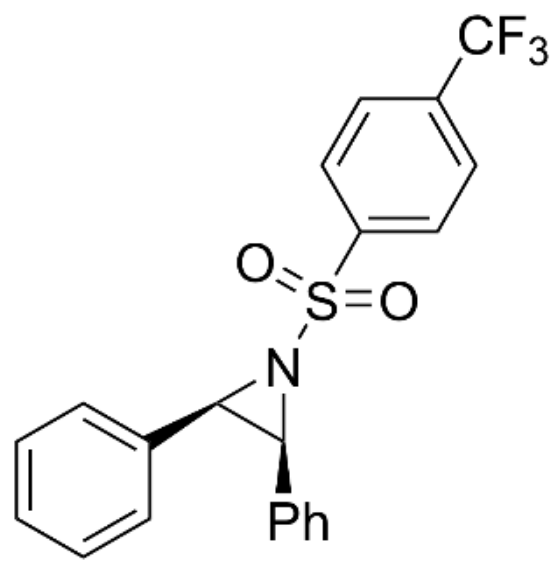

^{1}H NMR (400 MHz, CD_3CN): δ = 8.28 (d, J = 8.2 Hz, 2H), 7.95 (d, J = 8.3 Hz, 2H), 7.18 – 7.09 (m, 10 H), 7.23 – 7.17 (m, 2H), 4.35 (s, 2H). **^{13}C NMR** (101 MHz, CD_3CN): δ 142.3, 135.7 (q, J = 32.5 Hz), 132.9, 129.9, 129.0, 128.9, 128.6, 127. 6 (q, J = 3.7 Hz), 124.4 (q, J = 270.6 Hz), 48.8. **^{19}F NMR** (376 MHz, CD_3CN): δ = -63.8.

***trans*-2,3-Diphenyl-1-((4-(trifluoromethyl)phenyl)sulfonyl)aziridine:**

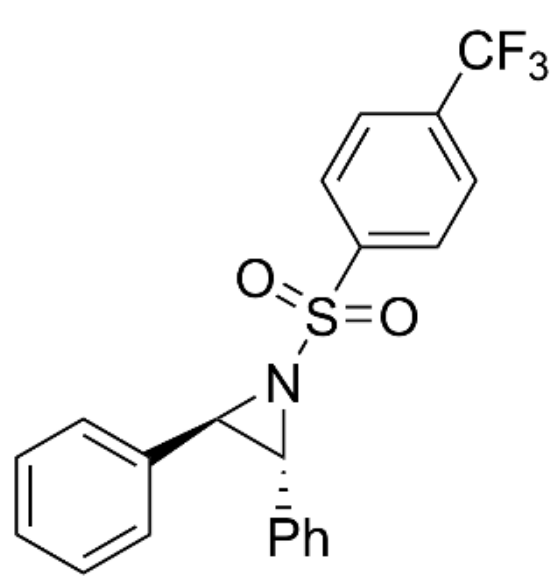

^{1}H NMR (400 MHz, CD_3CN): δ = 7.89 (d, J = 8.2 Hz, 2H), 7.77 (d, J = 8.3 Hz, 2H), 7.49 – 7.44 (m, 4 H), 7.40 – 7.34 (m, 6H), 4.47 (s, 2H). **^{13}C NMR** (101 MHz, CD_3CN): δ = 144.4, 134.9 (q, J = 32.5 Hz), 129.8, 129.4, 129.4, 129.3, 129.0, 127. 2 (q, J = 3.8 Hz), 124.4 (q, J = 270.5 Hz), 51.4. **^{19}F NMR** (376 MHz, CD_3CN): δ = -63.8.

1-((4-(Trifluoromethyl)phenyl)sulfonyl)-1a,2,3,7b-tetrahydro-1H-naphtho[1,2-b]azirine:

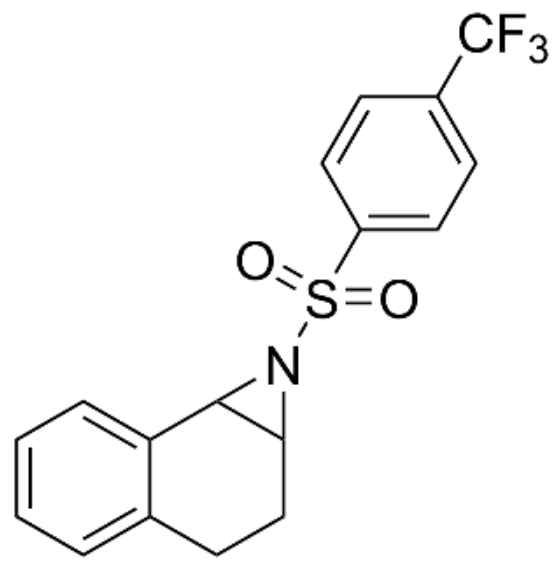

^{1}H NMR (400 MHz, $CDCl_3$): δ = 8.07 (d, J = 8.2 Hz, 2H), 7.78 (d, J = 8.2 Hz, 2H), 7.32 (dd, J = 7.4 Hz, J = 1.4 Hz 1H), 7.25 (dt, J = 7.4 Hz, J = 1.5 Hz, 1H), 7.19 (tt, J = 7.2 Hz, J = 1.4 Hz, 1H), 7.08 (d, J = 7.2 Hz, 1H), 3.91 (d, J = 7.1 Hz, 1H), 3.71 – 3.65 (m, 1H), 2.81 – 2.69 (m, 1H), 2.62 – 2.54 (m, 1H), 2.35 – 2.25 (m, 1H), 1.79 – 1.67 (m, 1H). **^{13}C NMR** (101 MHz, $CDCl_3$): δ = 142.6, 136.8, 135.2 (q, J = 32.9 Hz), 129.7, 129.6, 129.0, 128.9, 128.3, 126.7, 126.4 (q, J = 3.7 Hz), 123.4 (q, J = 271.4 Hz), 43.1, 42.6, 24.8, 20.2. **^{19}F NMR** (376 MHz, $CDCl_3$): δ = -63.2.

1-((4-(Trifluoromethyl)phenyl)sulfonyl)-1-azaspiro[2.5]octane:

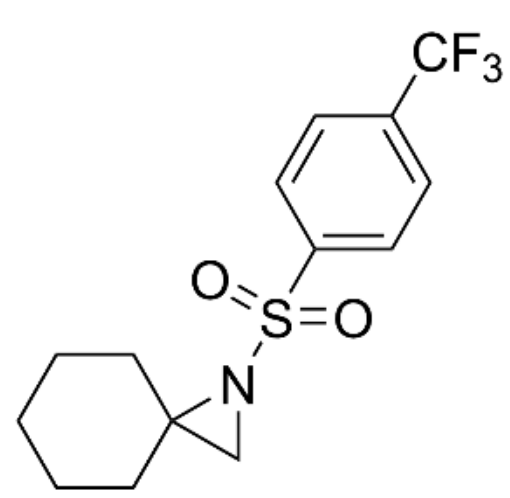

^{1}H NMR (400 MHz, $CDCl_3$): δ = 8.08 (d, J = 8.2 Hz, 2H), 7.78 (d, J = 8.3 Hz, 2H), 2,46 (br, 2H), 2.01 – 1.91 (m, 2H), 1.91 – 1.81 (m, 2H), 1.79 – 1.71 (m, 2H), 1.63 – 1.44 (m, 4H). **^{13}C NMR** (101 MHz, $CDCl_3$): δ = 142.6, 136.8, 135.2 (q, J = 32.9 Hz), 129.7, 129.6, 129.0, 128.9, 128.3, 126.7, 126.4 (q, J = 3.7 Hz), 123.4 (q, J = 271.4 Hz), 43.1, 42.6, 24.8, 20.2. **^{19}F NMR** (376 MHz, $CDCl_3$): δ = -63.2.

N-(Cyclohex-1-en-1-ylmethyl)-4-(trifluoromethyl)benzenesulfonamide:

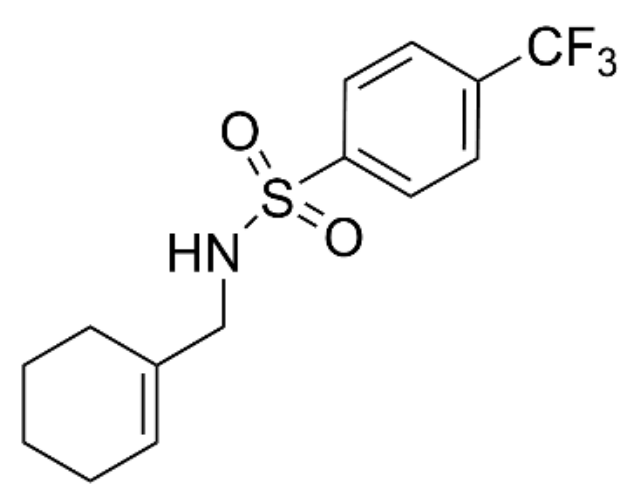

^{1}H NMR (400 MHz, $CDCl_3$): δ = 8.08 (d, J = 8.2 Hz, 2H), 7.78 (d, J = 8.3 Hz, 2H), 2,46 (br, 2H), 2.01 – 1.91 (m, 2H), 1.91 – 1.81 (m, 2H), 1.79 – 1.71 (m, 2H), 1.63 – 1.44 (m, 4H). **^{13}C NMR** (101 MHz, $CDCl_3$): δ = 144.2, 134.6 (q, J = 32.9 Hz), 132.5, 127.8, 126.3 (q, J = 3.7 Hz), 123.4 (q, J = 271.4 Hz), 50.0, 26.3, 25.1, 22.4, 22.1 **^{19}F NMR** (376 MHz, $CDCl_3$): δ = -63.1.

2-Butyl-1-((4-(trifluoromethyl)phenyl)sulfonyl)aziridine:

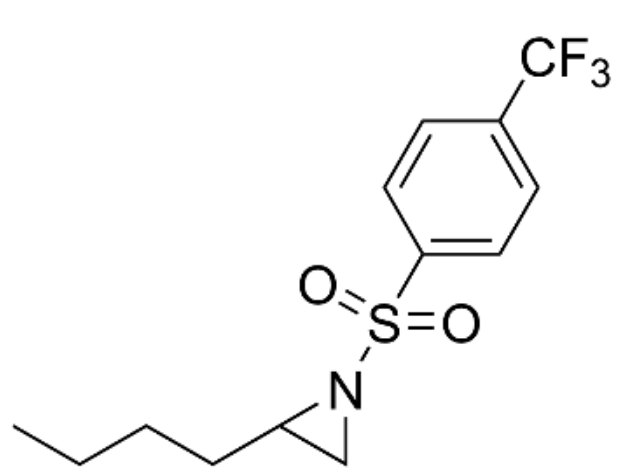

^{1}H NMR (400 MHz, $CDCl_3$): δ = 8.10 (d, J = 8.2 Hz, 2H), 7.82 (d, J = 8.3 Hz, 2H), 2.87 – 2.79 (m, 1H), 2.71 (d, J = 7 Hz, 1H), 2.12 (d, J = 4.7 Hz, 1H), 1.64 – 1.52 (m, 1H), 1.43 – 1.31 (m, 1H), 1.32 – 1.18 (m, 4H), 0.82 (t, J = 7 Hz, 3H). **^{13}C NMR** (101 MHz, $CDCl_3$): δ = 142.1, 135.3 (q, J = 32.9 Hz), 128.6, 126.3 (q, J = 3.7 Hz), 123.3 (q, J = 271.4 Hz), 41.2, 34.5, 31.1, 29.0, 22.2, 14.0. **^{19}F NMR** (376 MHz, $CDCl_3$): δ = -63.1.

2-Heptyl-1-((4-(trifluoromethyl)phenyl)sulfonyl)aziridine:

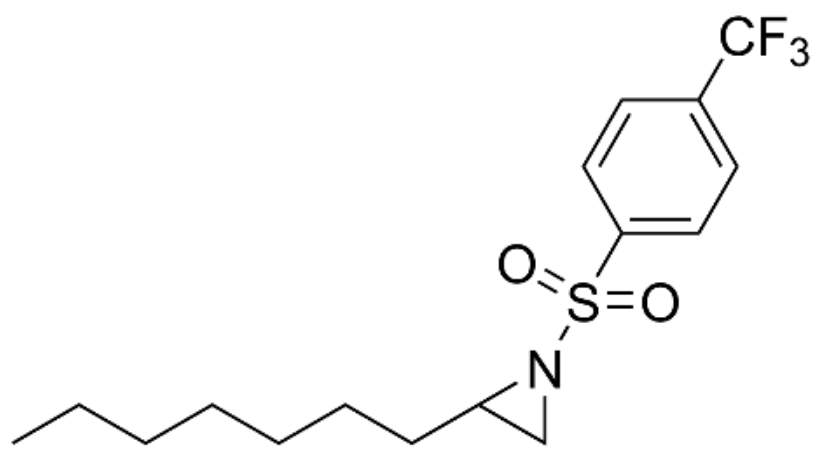

^{1}H NMR (400 MHz, $CDCl_3$): δ = 8.10 (d, J = 8.2 Hz, 2H), 7.81 (d, J = 8.2 Hz, 2H), 2.87 – 2.79 (m, 1H), 2.86 – 2.79 (m, 1H), 2.71 (d, J = 7 Hz, 1H), 2.12 (d, J = 4.7 Hz, 1H), 1.62 – 1.51 (m, 1H), 1.40 – 1.14 (m, 11 H), 0.86 (t, J = 7.2 Hz, 3H). **^{13}C NMR** (101 MHz, $CDCl_3$): δ = 142.1, 135.3 (q, J = 33 Hz), 128.6, 126.3 (q, J = 3.7 Hz), 123.3 (q, J = 271.3 Hz), 41.2, 34.5, 31.8, 29.2, 29.1, 26.9, 22.7, 14.1. **^{19}F NMR** (376 MHz, $CDCl_3$): δ = -63.1.

***cis*-2-Methyl-3-propyl-1-((4-(trifluoromethyl)phenyl)sulfonyl)aziridine:**

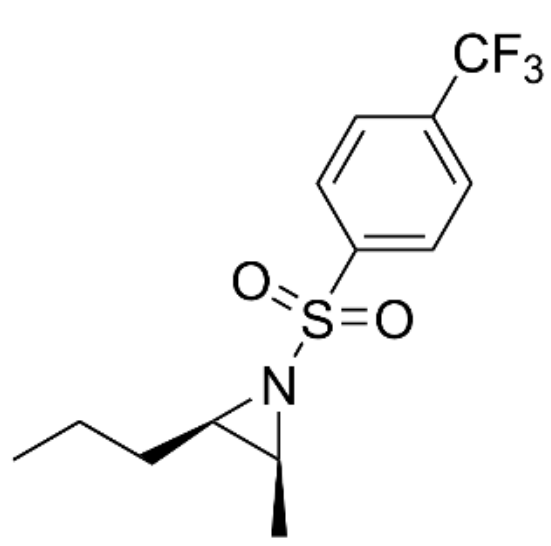

^{1}H NMR (400 MHz, $CDCl_3$): δ = 8.08 (d, J = 8.2 Hz, 2H), 7.81 (d, J = 8.2 Hz, 2H), 3.04 – 2.96 (m, 1H), 2.90 – 2.83 (m, 1H), 1.51 – 1.37 (m, 1H), 1.36 – 1.25 (m, 1H), 1.22 (d, J = 5.8 Hz, 1H), 0.89 (t, J = 7.2 Hz, 3H). **^{13}C NMR** (101 MHz, $CDCl_3$): δ = 142.1, 135.3 (q, J = 32.9 Hz), 128.6, 126.3 (q, J = 3.7 Hz), 123.3 (q, J = 271.4 Hz), 41.2, 34.5, 31.1, 29.0, 22.2, 14.0. **^{19}F NMR** (376 MHz, $CDCl_3$): δ = -63.1.

***trans*-2-Methyl-3-propyl-1-((4-(trifluoromethyl)phenyl)sulfonyl)aziridine:**

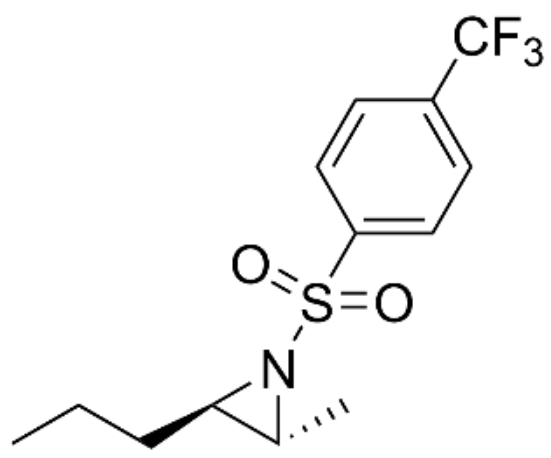

^{1}H NMR (400 MHz, $CDCl_3$): δ = 8.09 (d, J = 8.2 Hz, 2H), 7.79 (d, J = 8.3 Hz, 2H), 2.80 – 2.72 (m, 2H), 1.64 – 1.52 (m, 5H), 1.36 – 1.24 (m, 2H), 0.88 (t, J = 7.4 Hz, 3H). **^{13}C NMR** (101 MHz, $CDCl_3$): δ = 144.6, 134.7 (q, J = 32.8 Hz), 128.0, 126.2 (q, J = 3.7 Hz), 123.4 (q, J = 271.3 Hz), 50.5, 46.7, 32.6, 20.6, 15.2, 13.2. **^{19}F NMR** (376 MHz, $CDCl_3$): δ = -63.1

2-Benzyl-1-((4-(trifluoromethyl)phenyl)sulfonyl)aziridine:

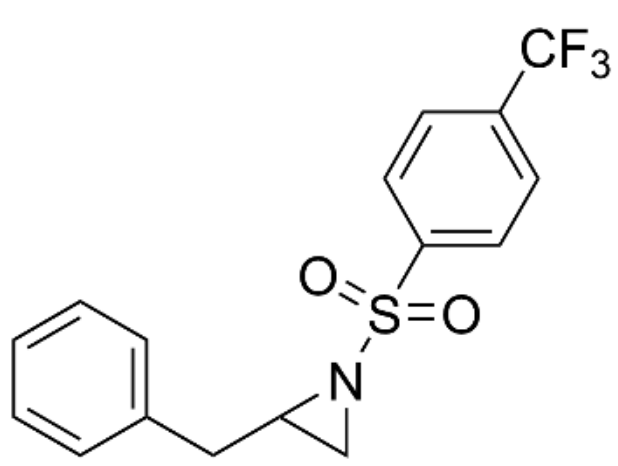

^{1}H NMR (400 MHz, $CDCl_3$): δ =7.85 (d, J = 8. Hz, 2H), 7.62 (d, J = 8.2 Hz, 2H), 7.16 – 7.07 (m, 3H), 7.00 – 6.95 (m, 2H), 3.05 – 2.97 (m, 1H), 2.94 (dd, J = 14.3 Hz, J = 4.4 Hz 1H), 2.85 (d, J = 6.8 Hz, 1 H), 2.52 (dd, J = 14.3 Hz, J = 8.1 Hz 1H), 2.27 (d, J = 4.6 Hz, 1H). **^{13}C NMR** (101 MHz, $CDCl_3$): δ = 141.5, 137.0, 135.0 (q, J = 32.8 Hz), 128.7, 128.6, 128.4, 127.4, 127.0, 126.1 (q, J = 3.7 Hz), 123.3 (q, J = 271.6 Hz), 42.6, 37.7, 33.2. **^{19}F NMR** (376 MHz, $CDCl_3$): δ = -63.8.

2-Phenethyl-1-((4-(trifluoromethyl)phenyl)sulfonyl)aziridine:

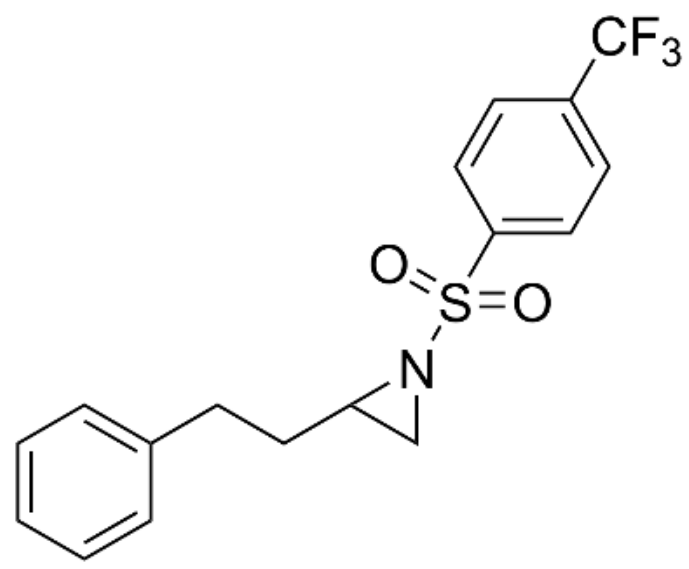

^{1}H NMR (400 MHz, $CDCl_3$): δ = 8.09 (d, J = 8.1 Hz, 2H), 7.82 (d, J = 8.0 Hz, 2H), 7.23 – 7.17 (m, 3H), 7.15 – 7.11 (m, 2H), 2.95 – 2.86 (m, 1H), 2.73 – 2.59 (m, 3H), 2.11 (d, J = 4.7 Hz, 1 H), 1.97 – 1.86 (m, 1H), 1.80 (m, 1H). **^{13}C NMR** (101 MHz, $CDCl_3$): δ = 142.0, 140.5, 135.3 (q, J = 32.8 Hz), 128.7, 128.6, 128.4, 126.4, 126.3 (q, J = 3.7 Hz), 123.3 (q, J = 271.6 Hz), 40.3, 34.7, 33.1, 33.0. **^{19}F NMR** (376 MHz, $CDCl_3$): δ = -63.2.

^{1}H NMR of L-1 in $CDCl_3$

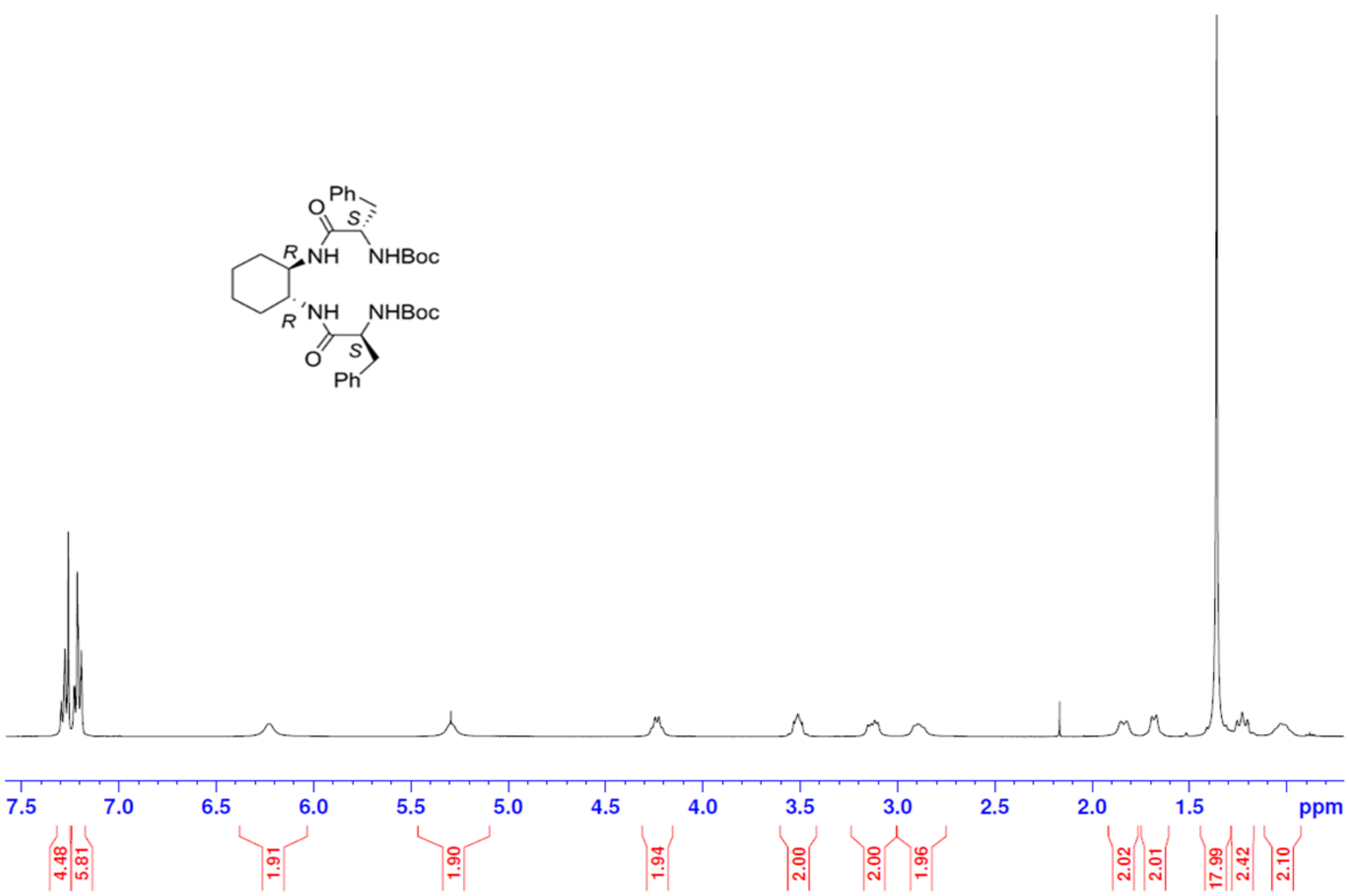

^{13}C NMR of L-1 in $CDCl_3$

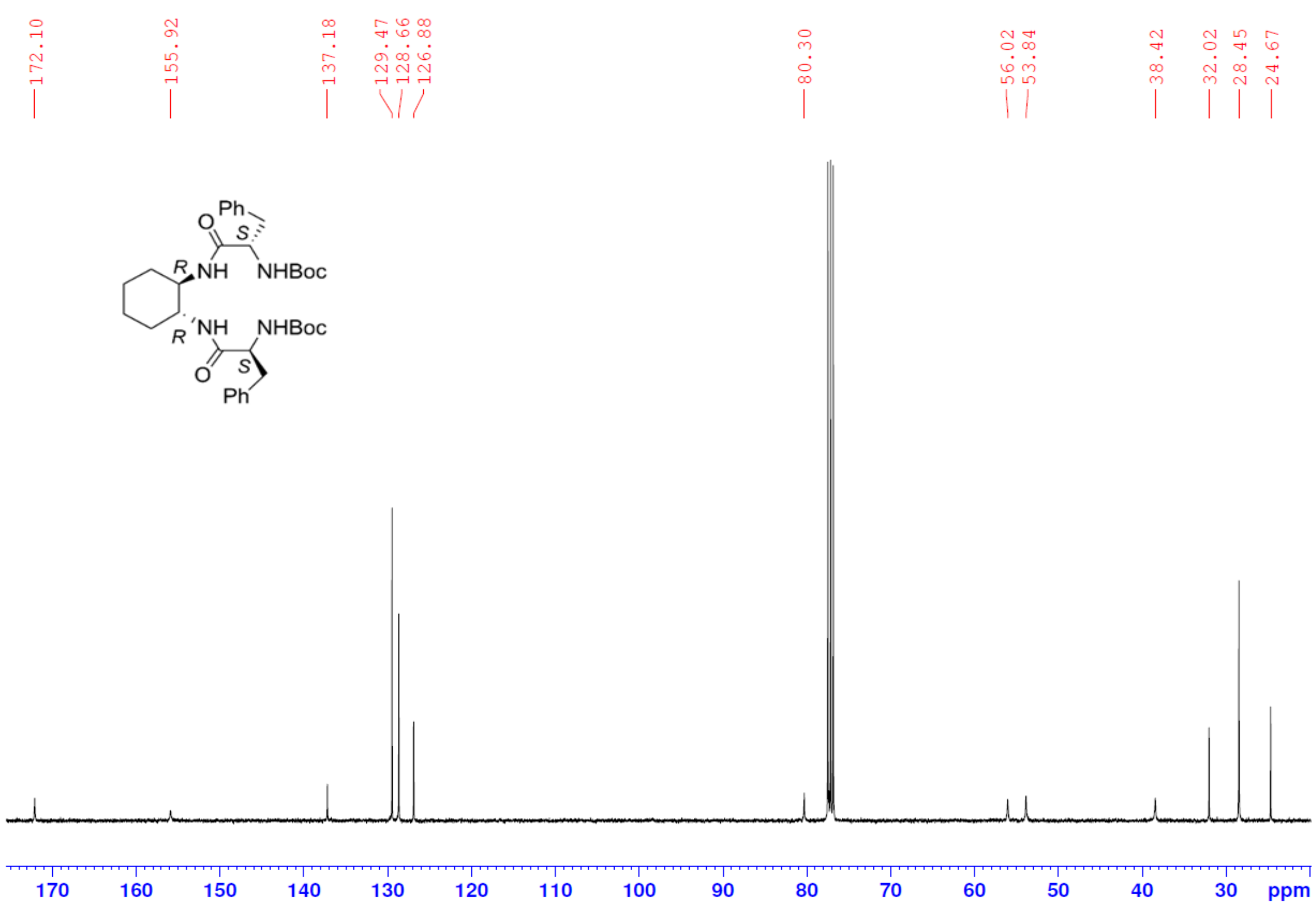

^{1}H NMR of L-2 in $CDCl_3$

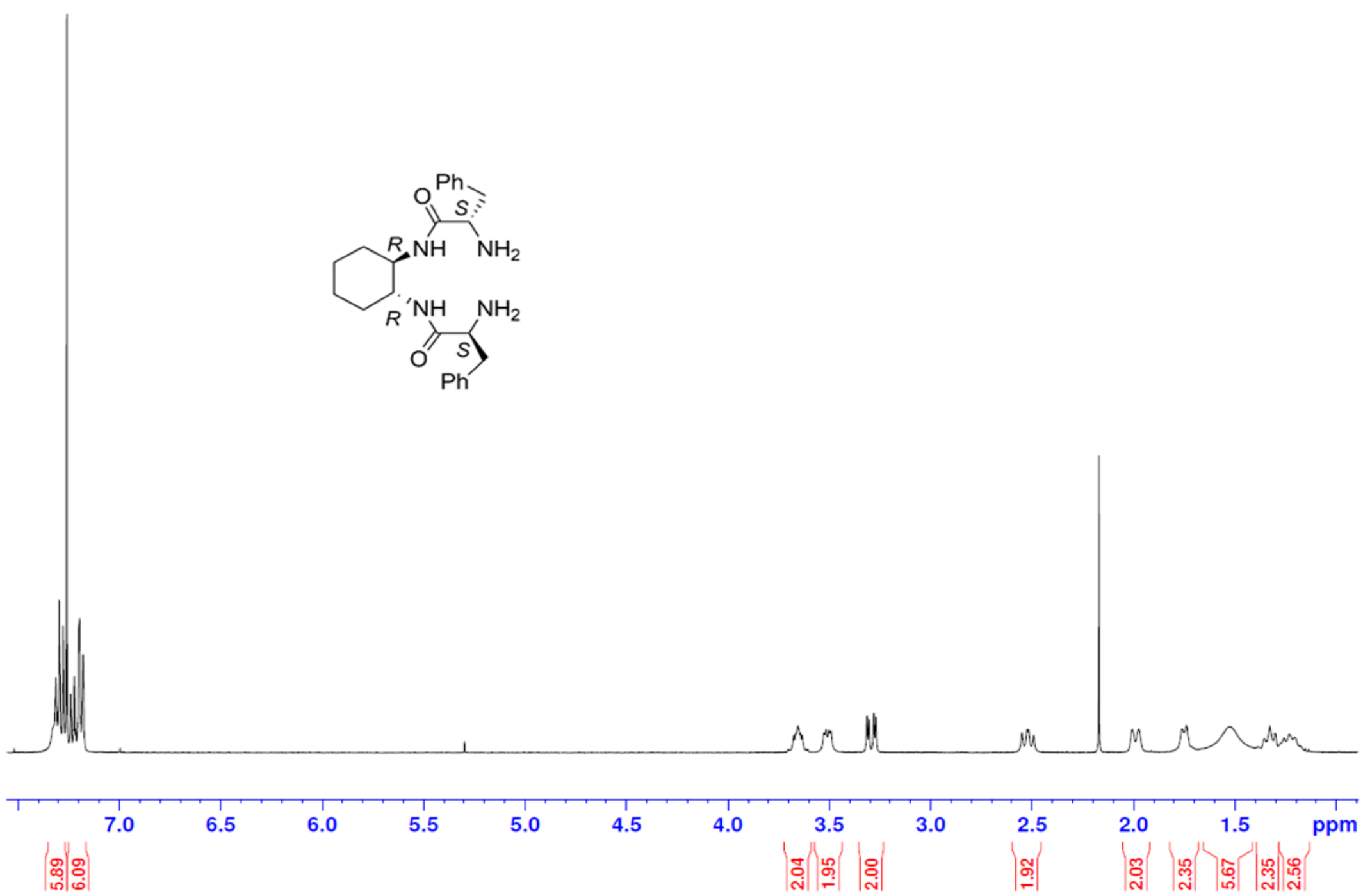

^{13}C NMR of L-2 in $CDCl_3$

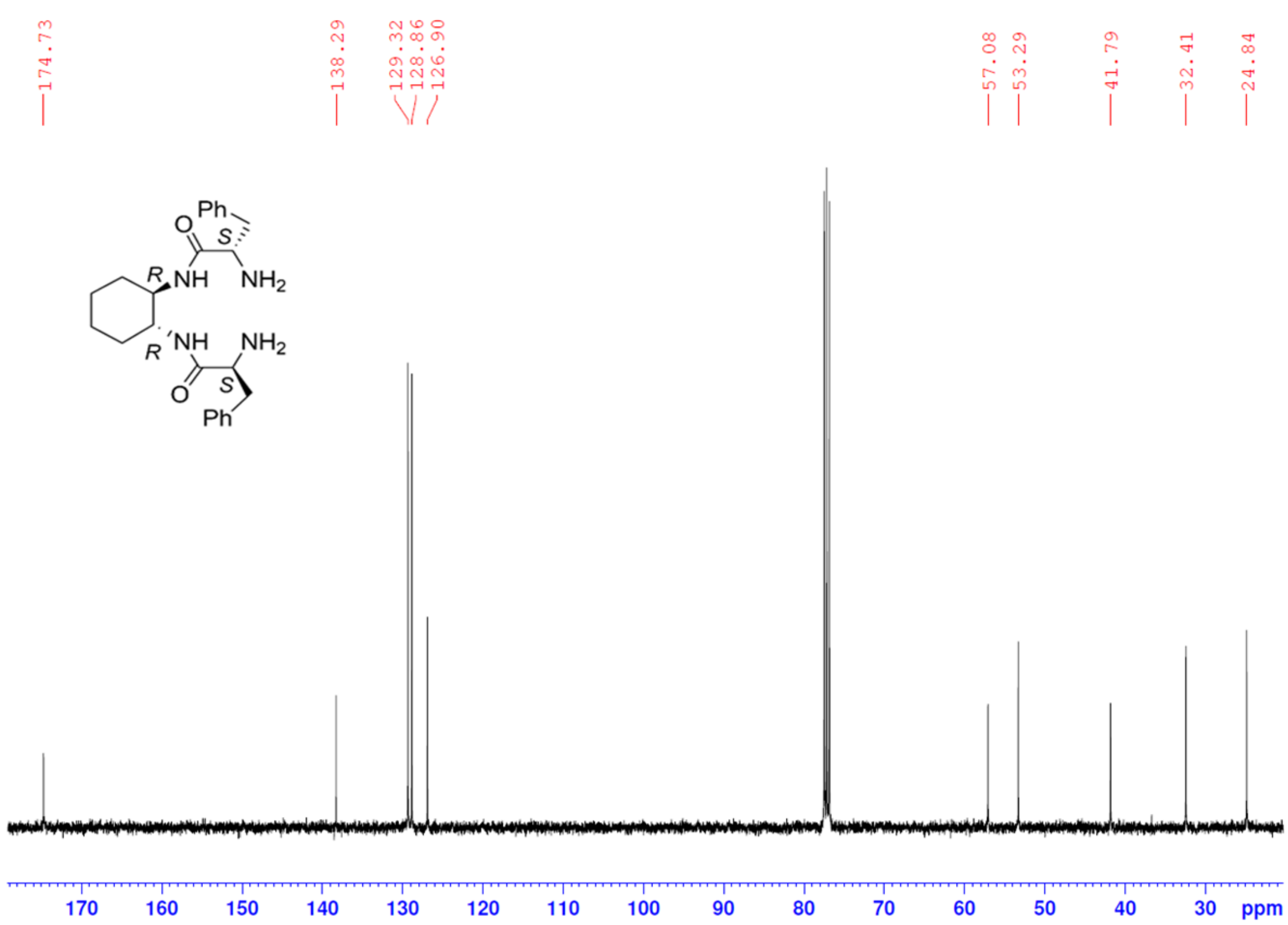

^{1}H NMR of $L^{1}H_4$ in $(CD_3)_2SO$

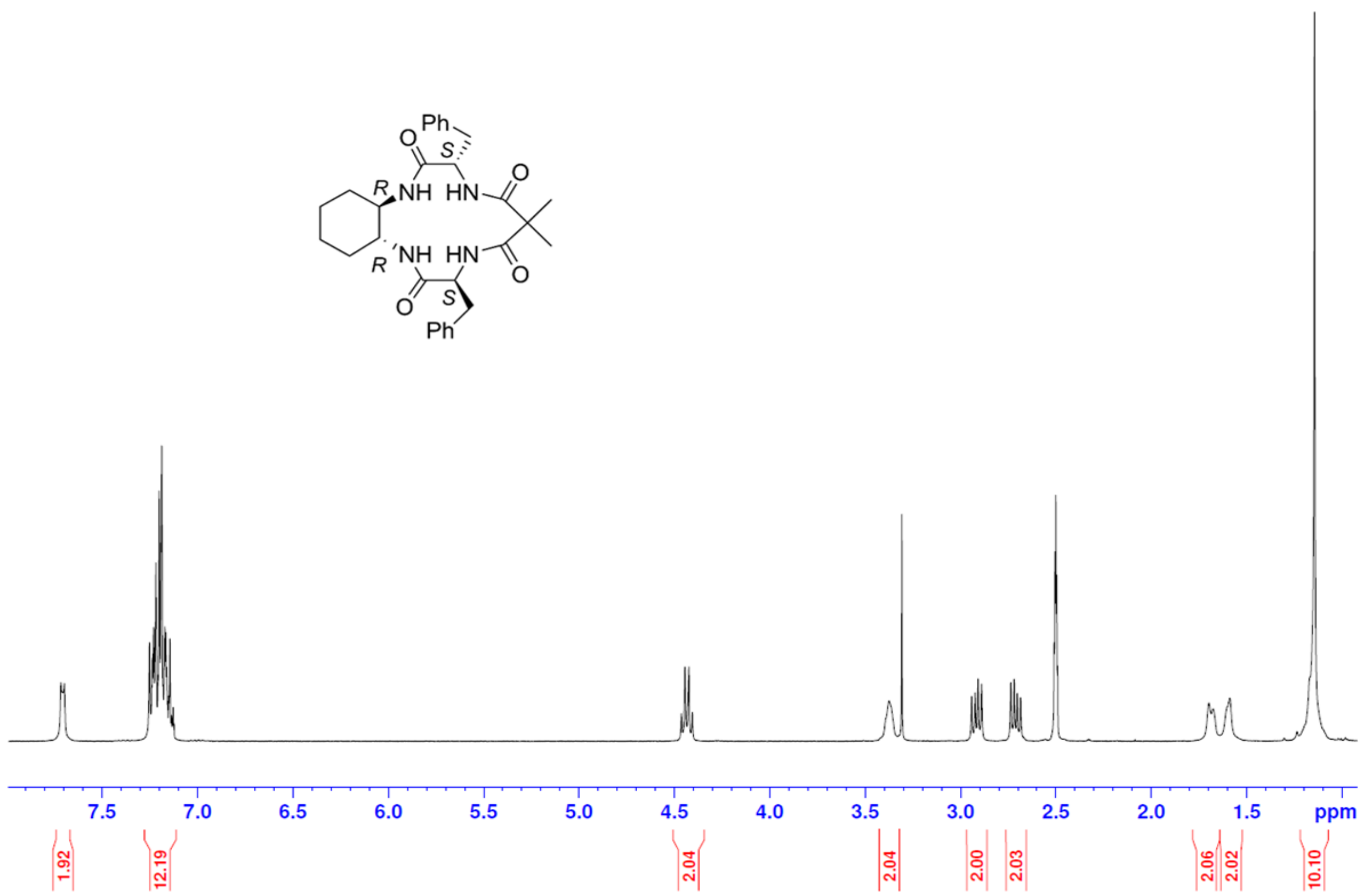

^{13}C NMR of $L^{1}H_4$ in $(CD_3)_2SO$

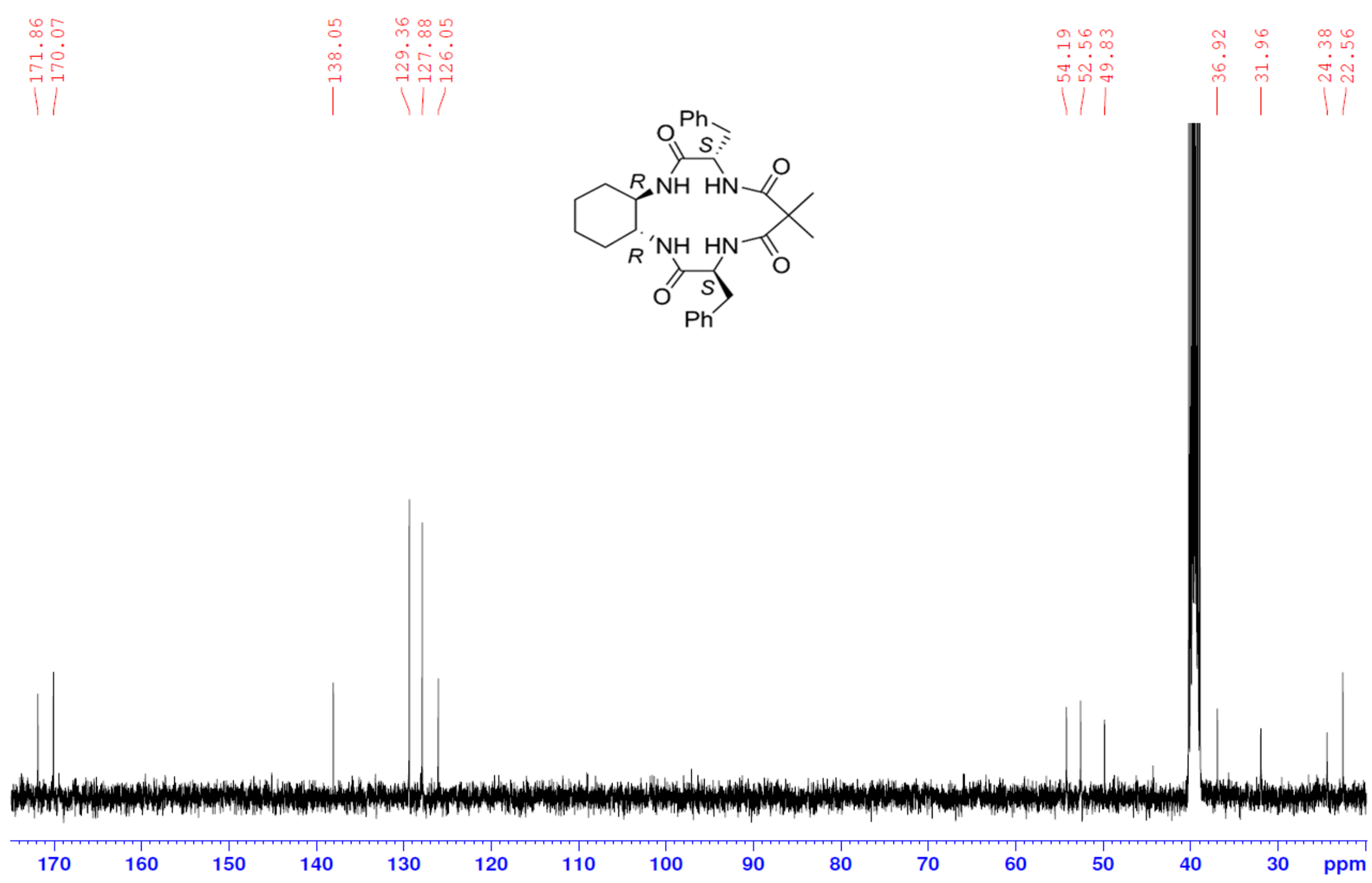

^{1}H NMR of (PPh$_4$)[Co^{III}(L^1)] in CD$_2$Cl$_2$

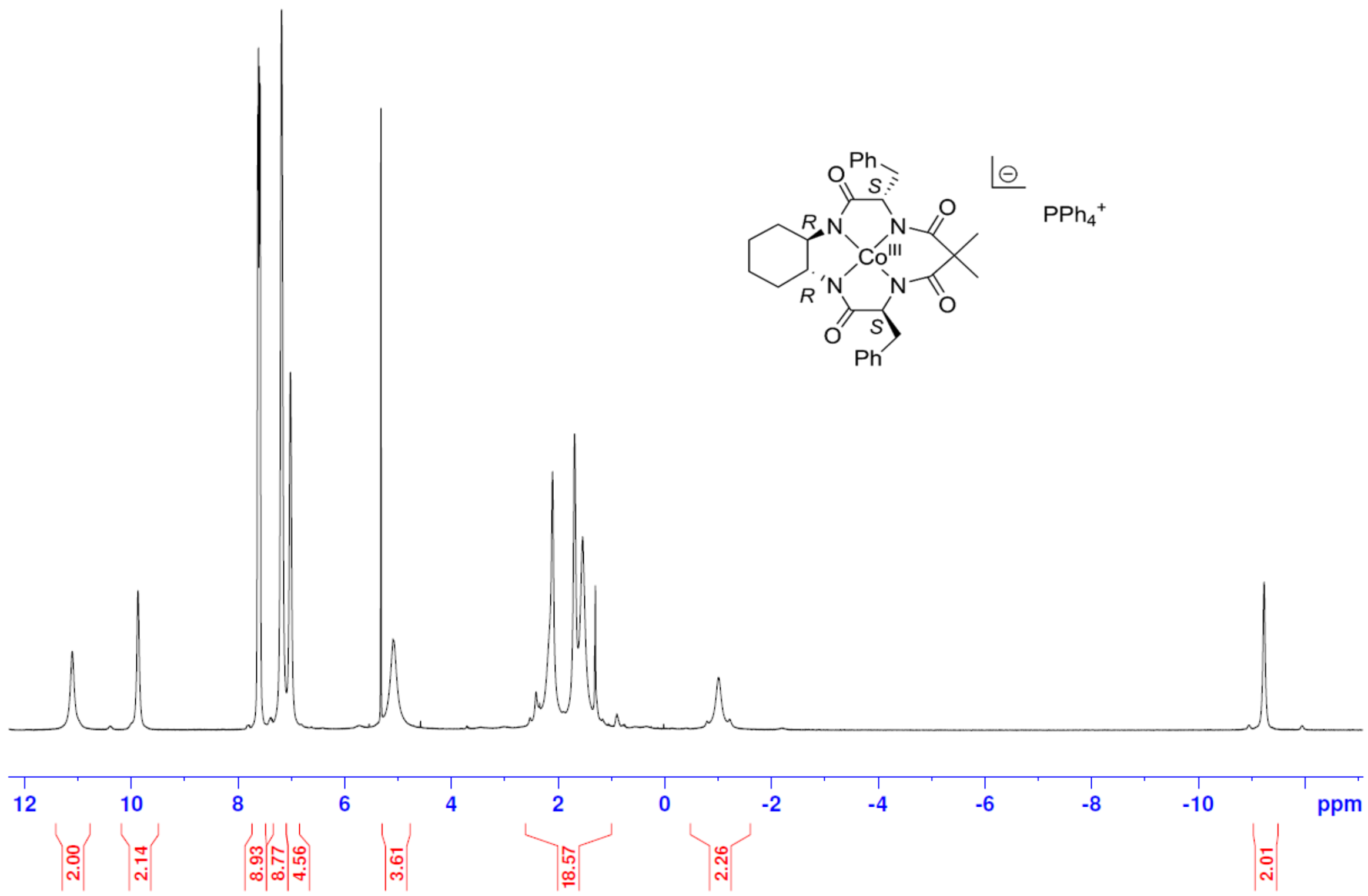

Quantification of products of cis-β-Methylstyrene with PhI=NTs (top) and PhI=NSO$_2$-C$_6$H$_4$-*p*-CF$_3$ (bottom) in CDCl$_3$ by ^{1}H NMR

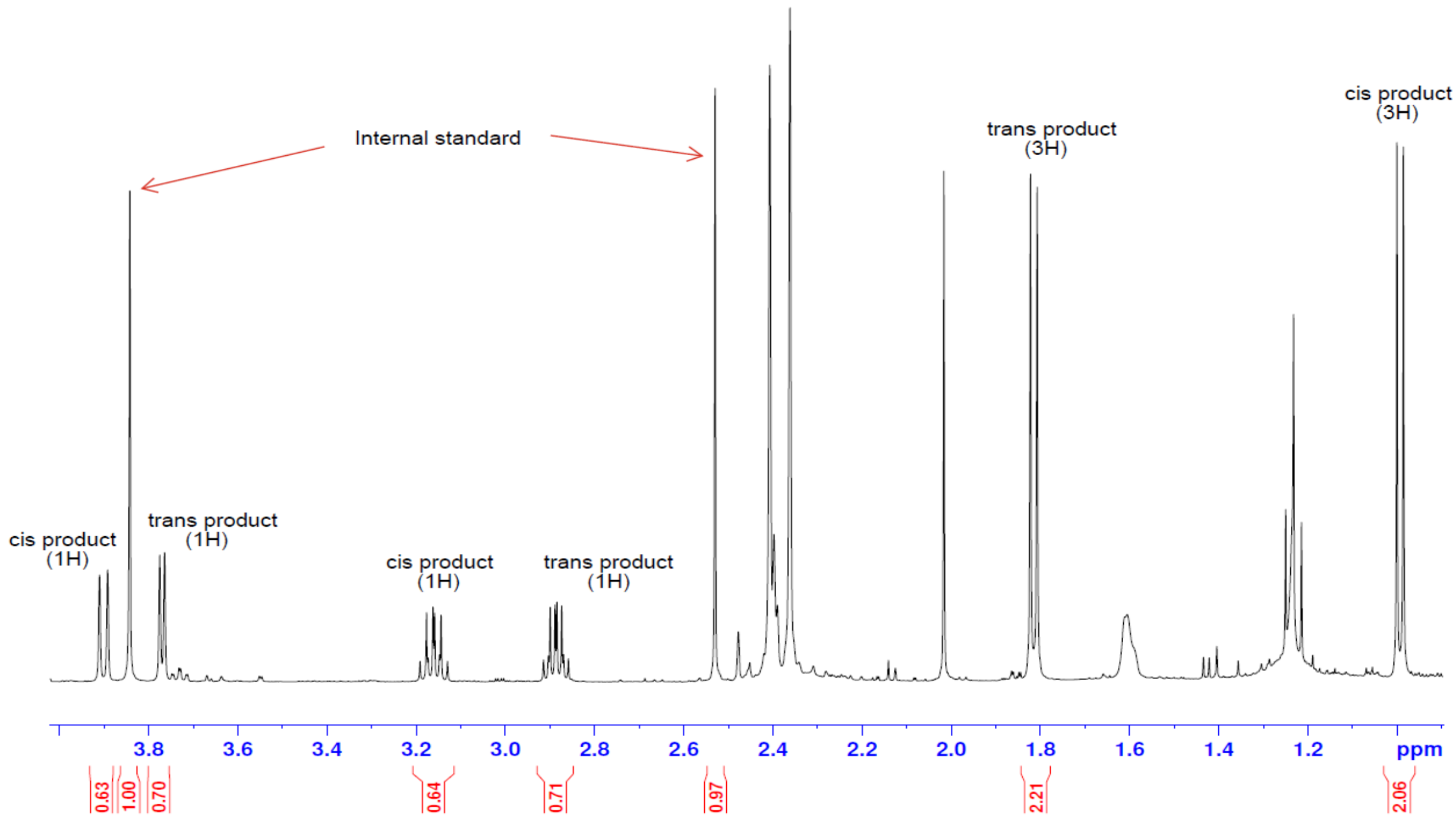

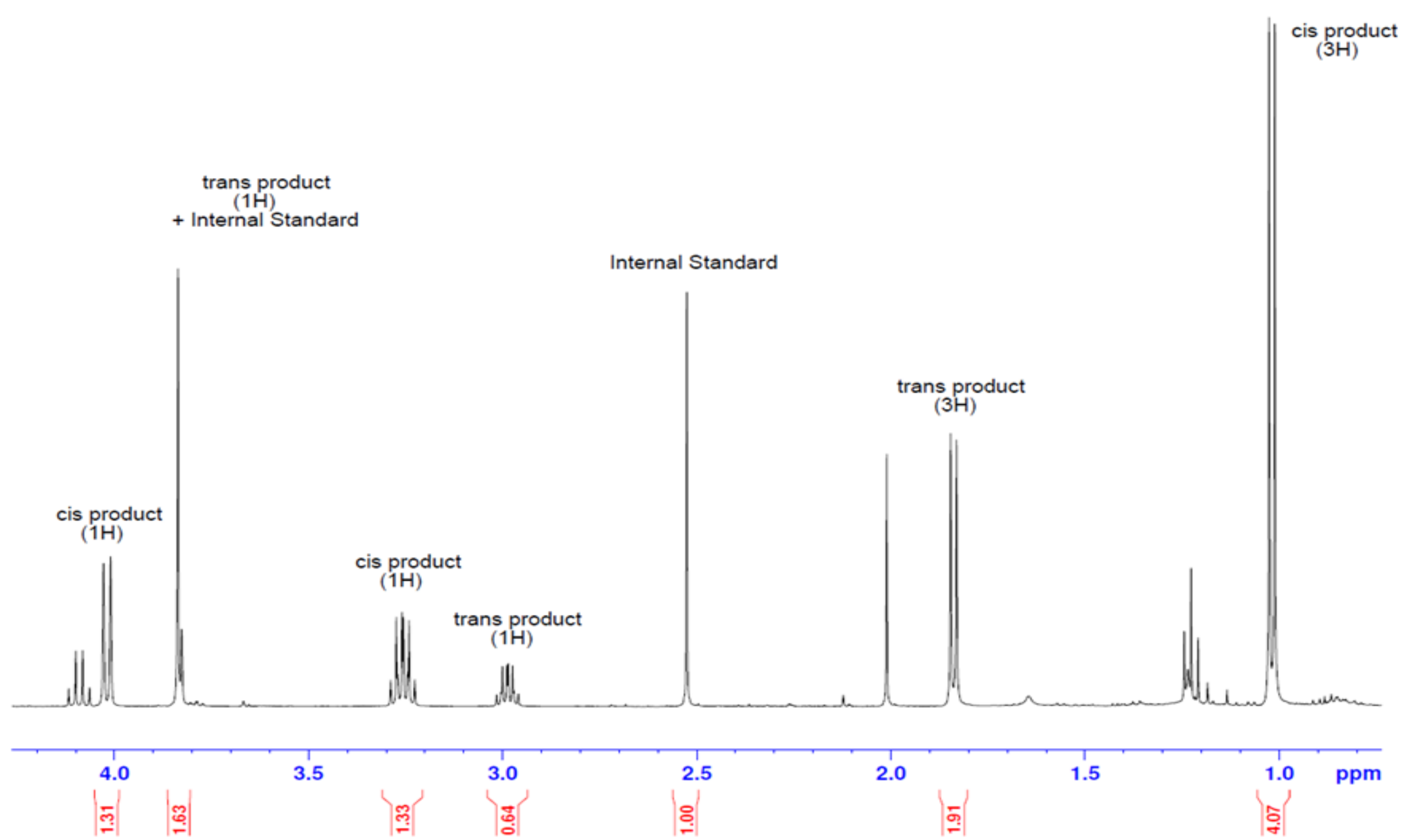

Quantification of products of *cis*-stilbene with PhI=NTs (top) and PhI=NSO$_2$-C$_6$H$_4$-*p*-CF$_3$ (bottom) in CDCl$_3$ by ^{1}H NMR

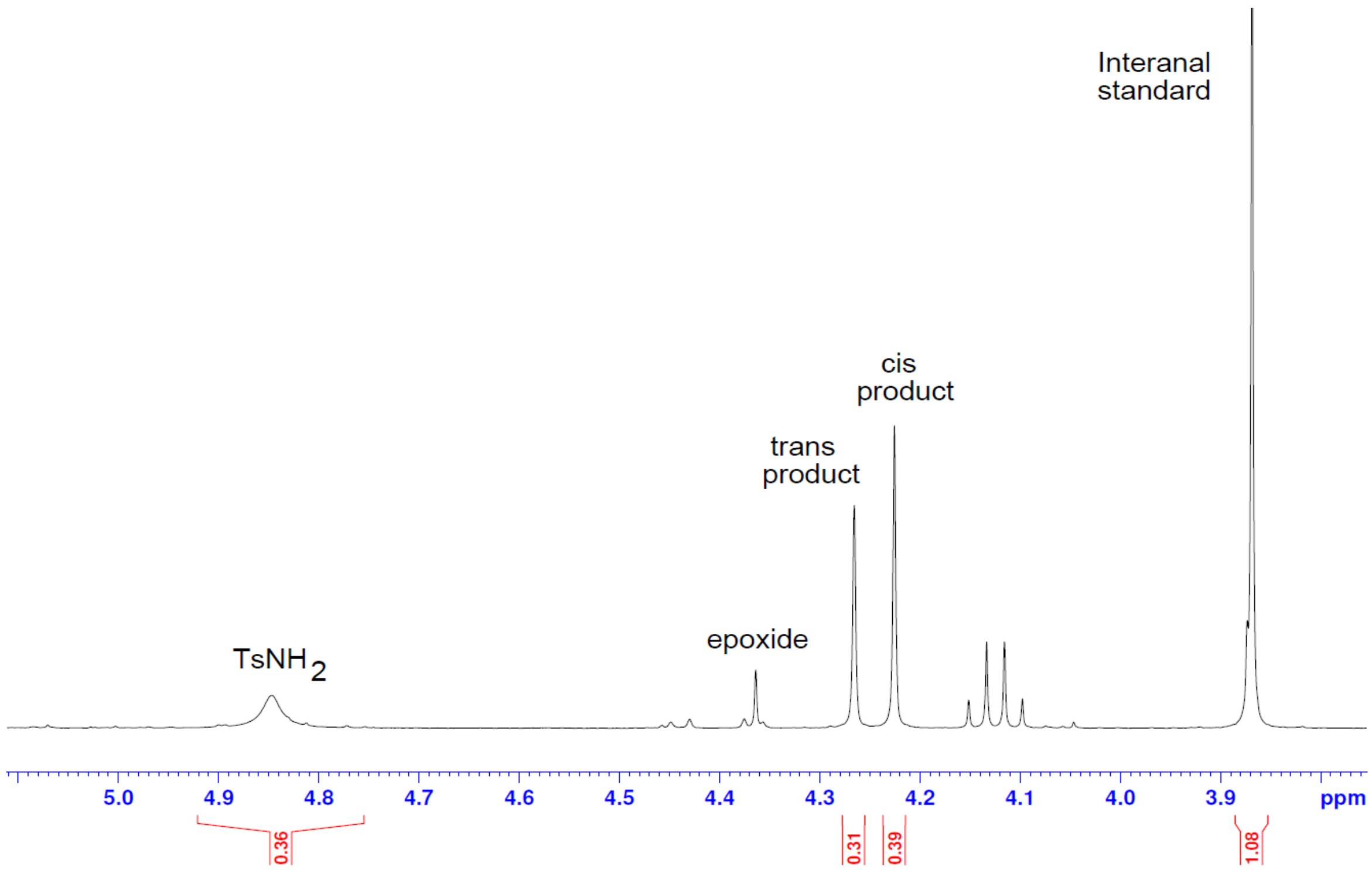

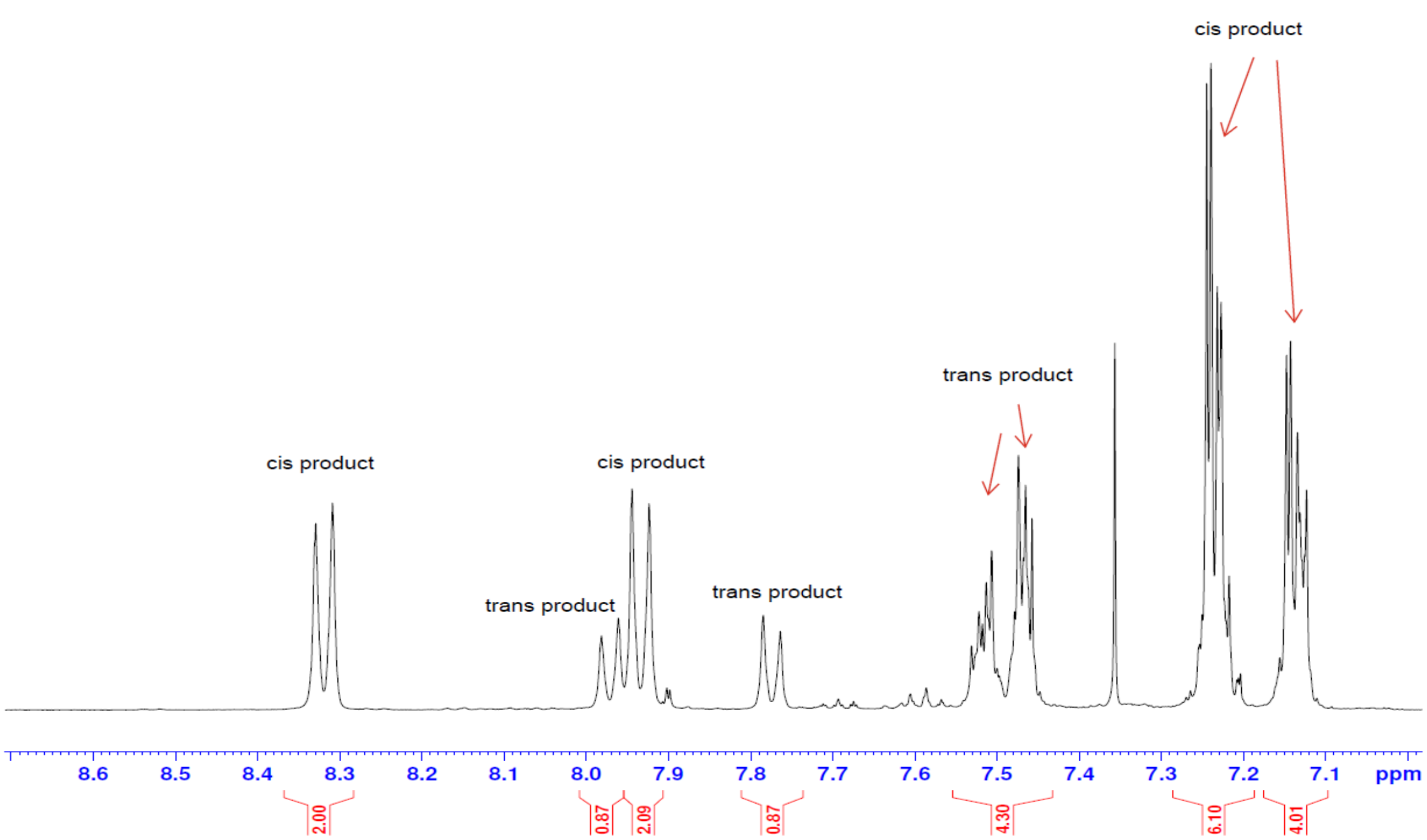

Quantification of products of *cis*-2-hexene with PhI=NTs (top) and PhI=NSO$_2$-C$_6$H$_4$-*p*-CF$_3$ (bottom) in CDCl$_3$ by ^{1}H NMR

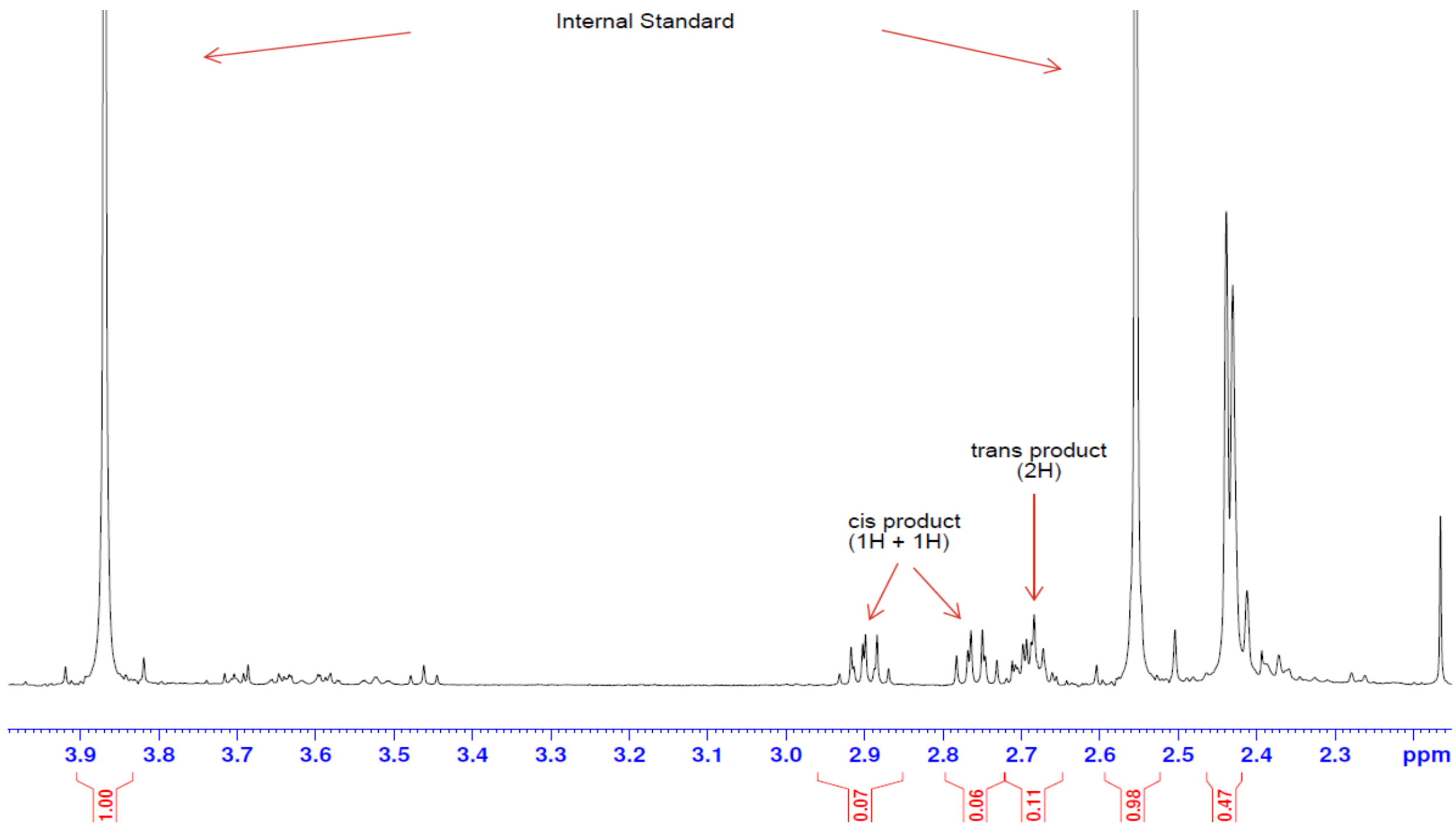

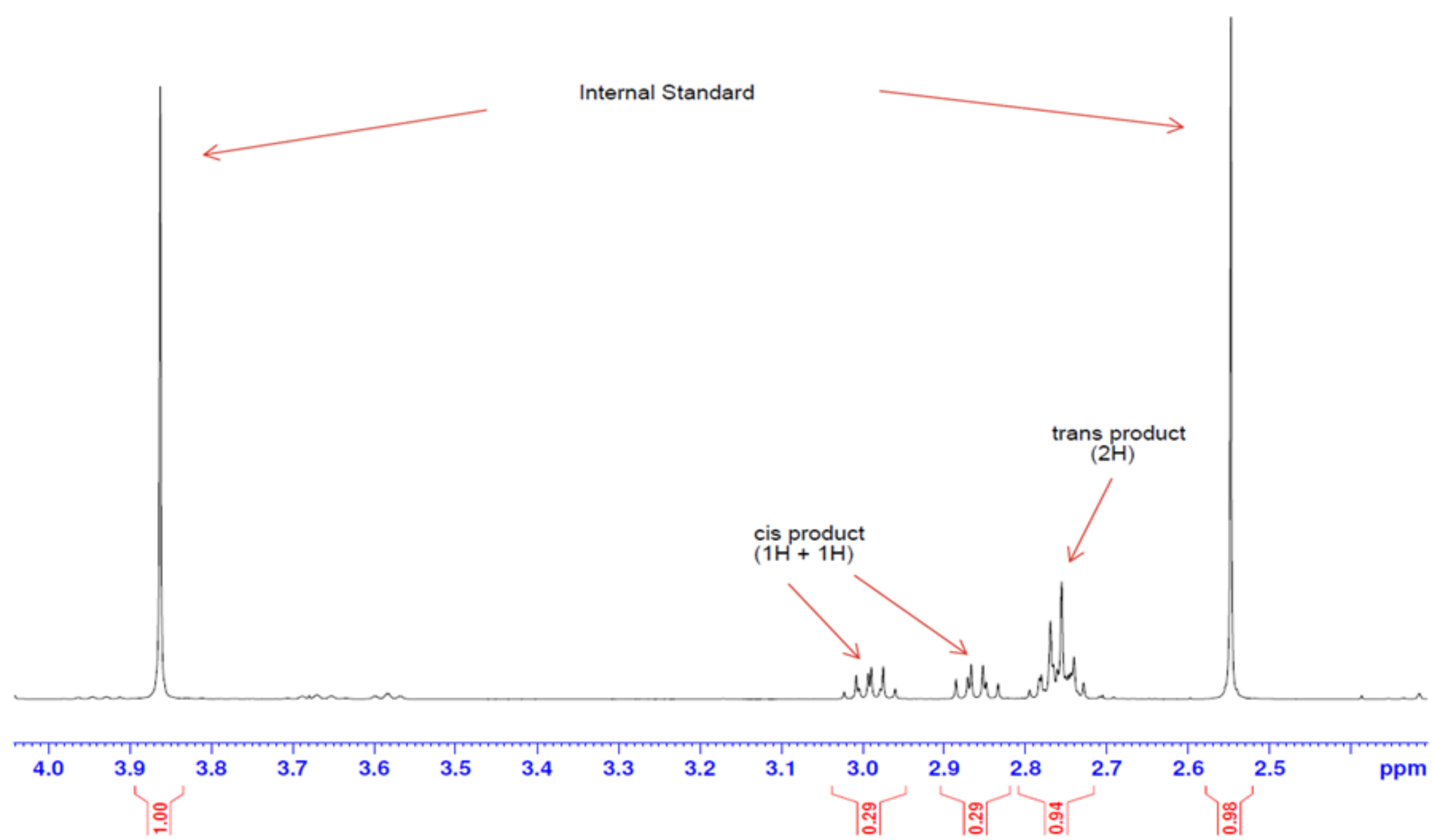

^{1}H NMR Spectra of Products in $CDCl_3$ (or as mentioned otherwise):

2-(p-Tolyl)-1-((4-(trifluoromethyl)phenyl)sulfonyl)aziridine :

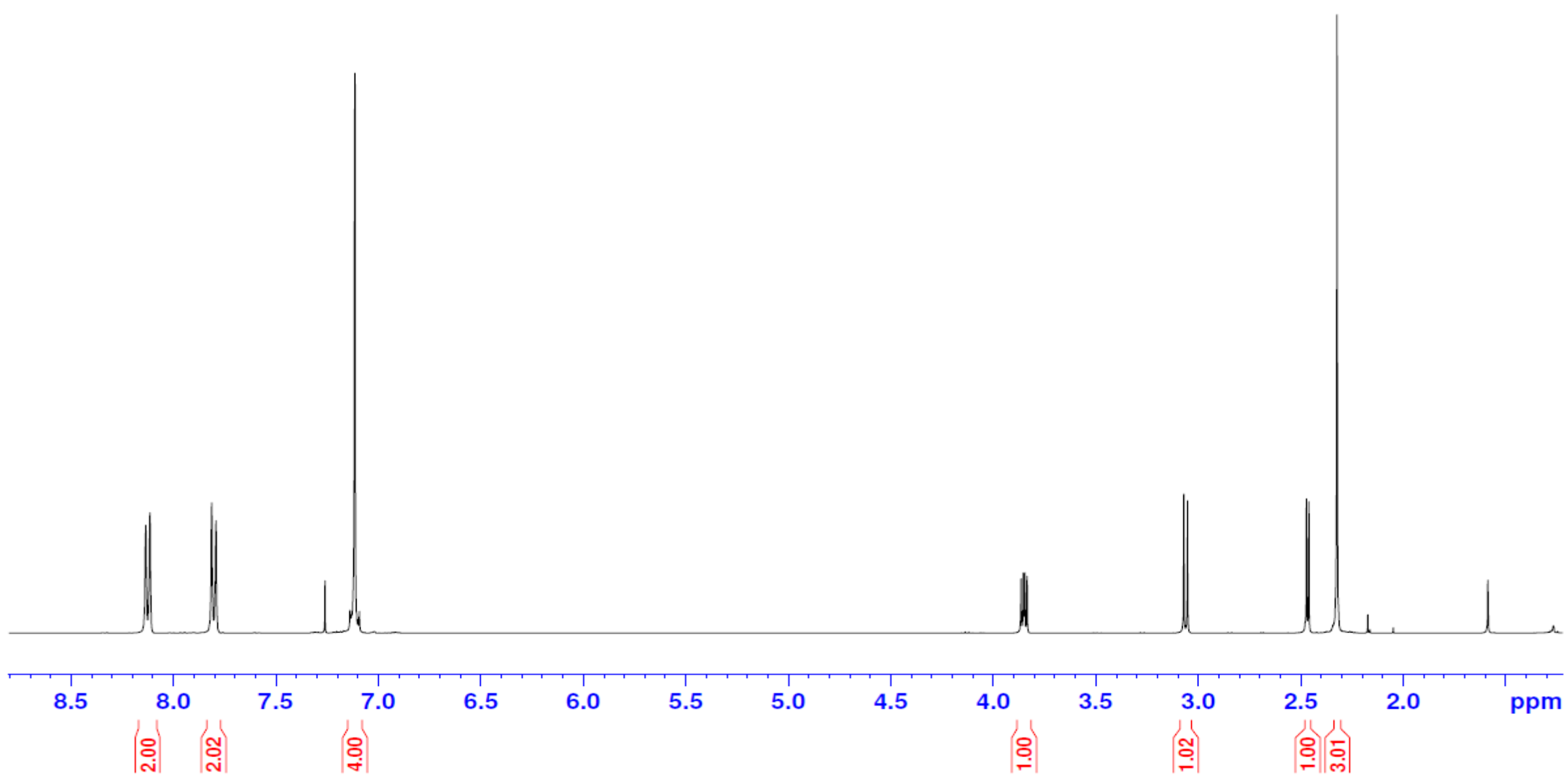

2-(4-(Tert-butyl)phenyl)-1-((4-(trifluoromethyl)phenyl)sulfonyl)aziridine:

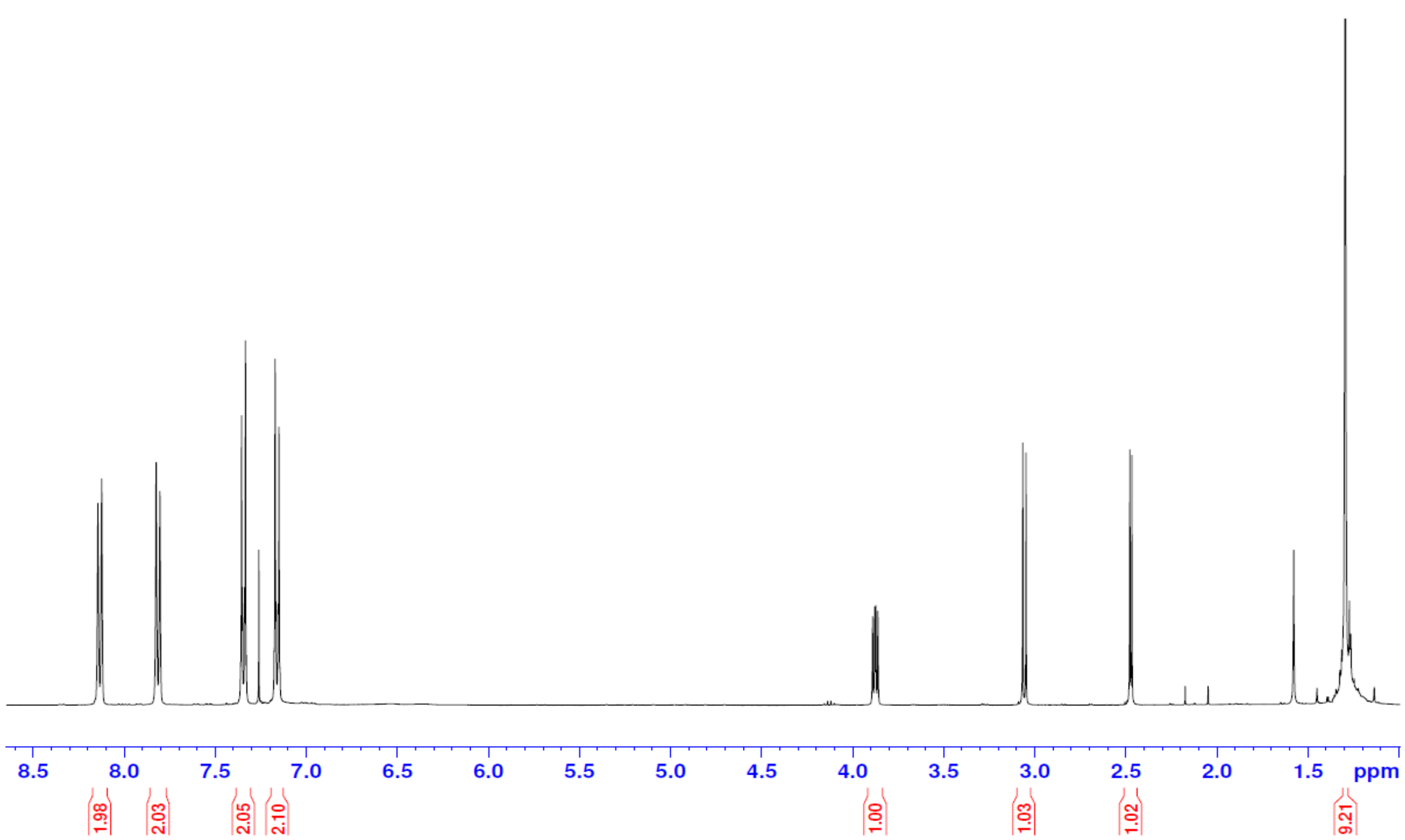

2-(4-Chlorophenyl)-1-((4-(trifluoromethyl)phenyl)sulfonyl)aziridine:

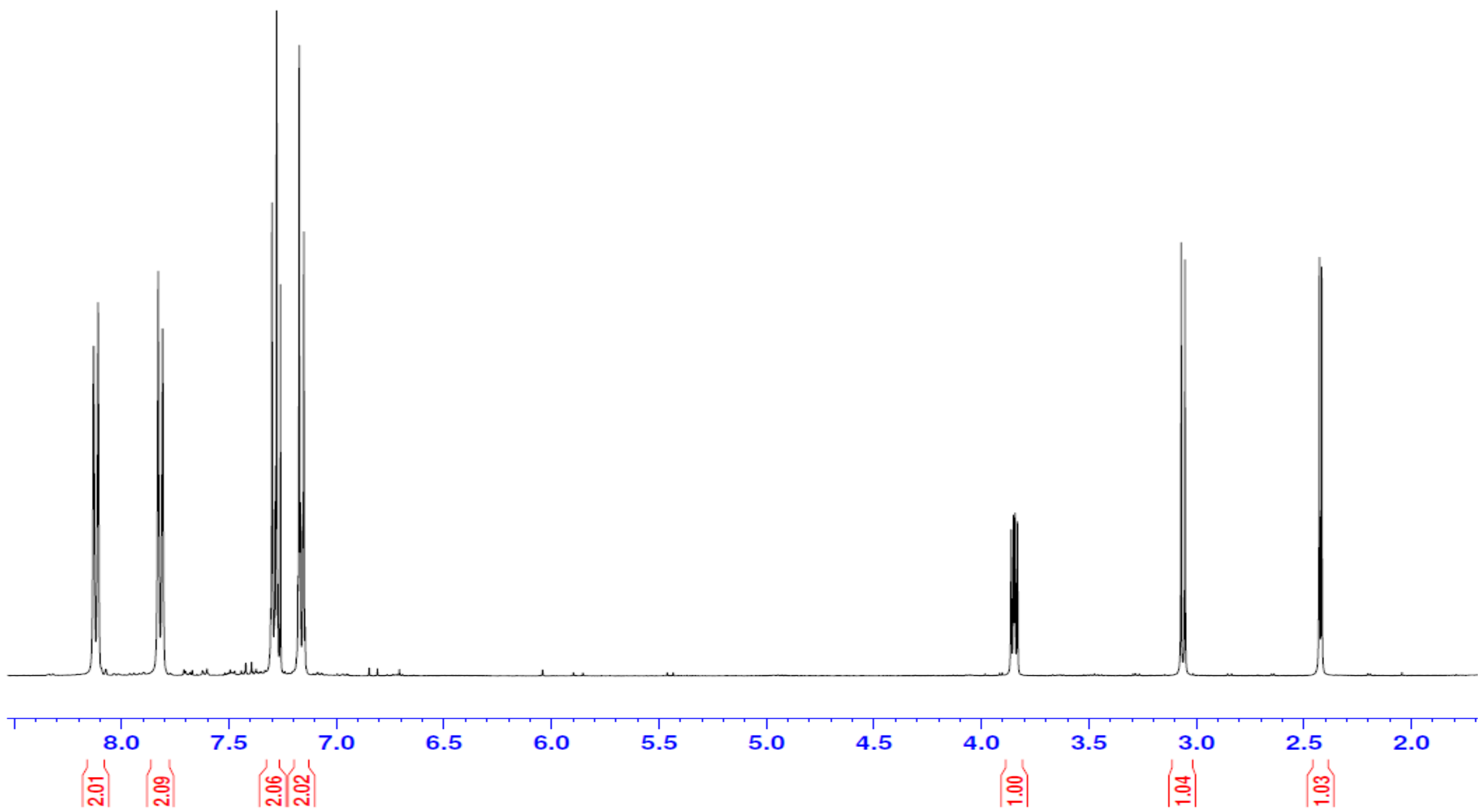

2-(4-Fluorophenyl)-1-((4-(trifluoromethyl)phenyl)sulfonyl)aziridine:

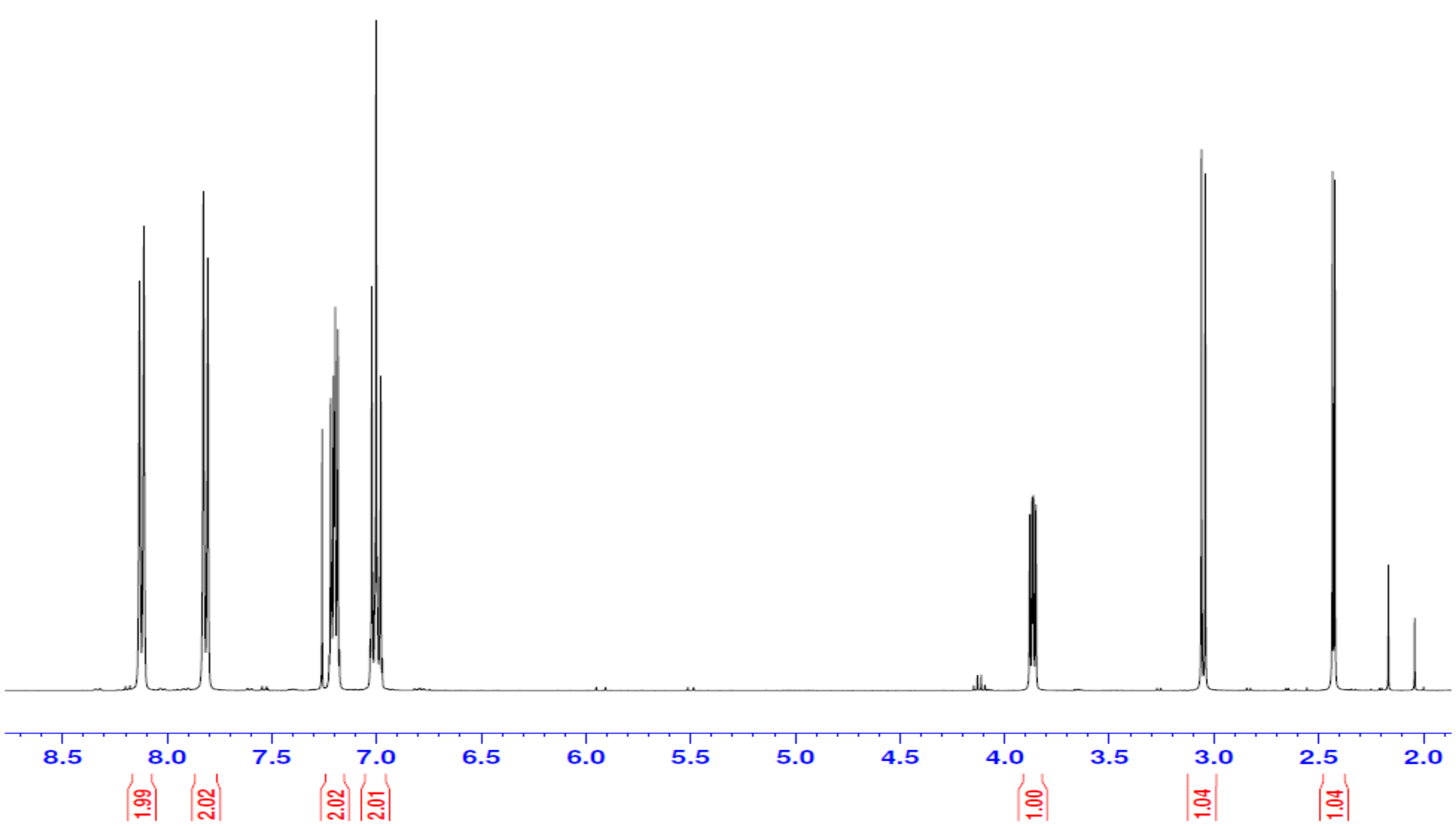

2-(4-(Trifluoromethyl)phenyl)-1-((4-(trifluoromethyl)phenyl)sulfonyl)aziridine:

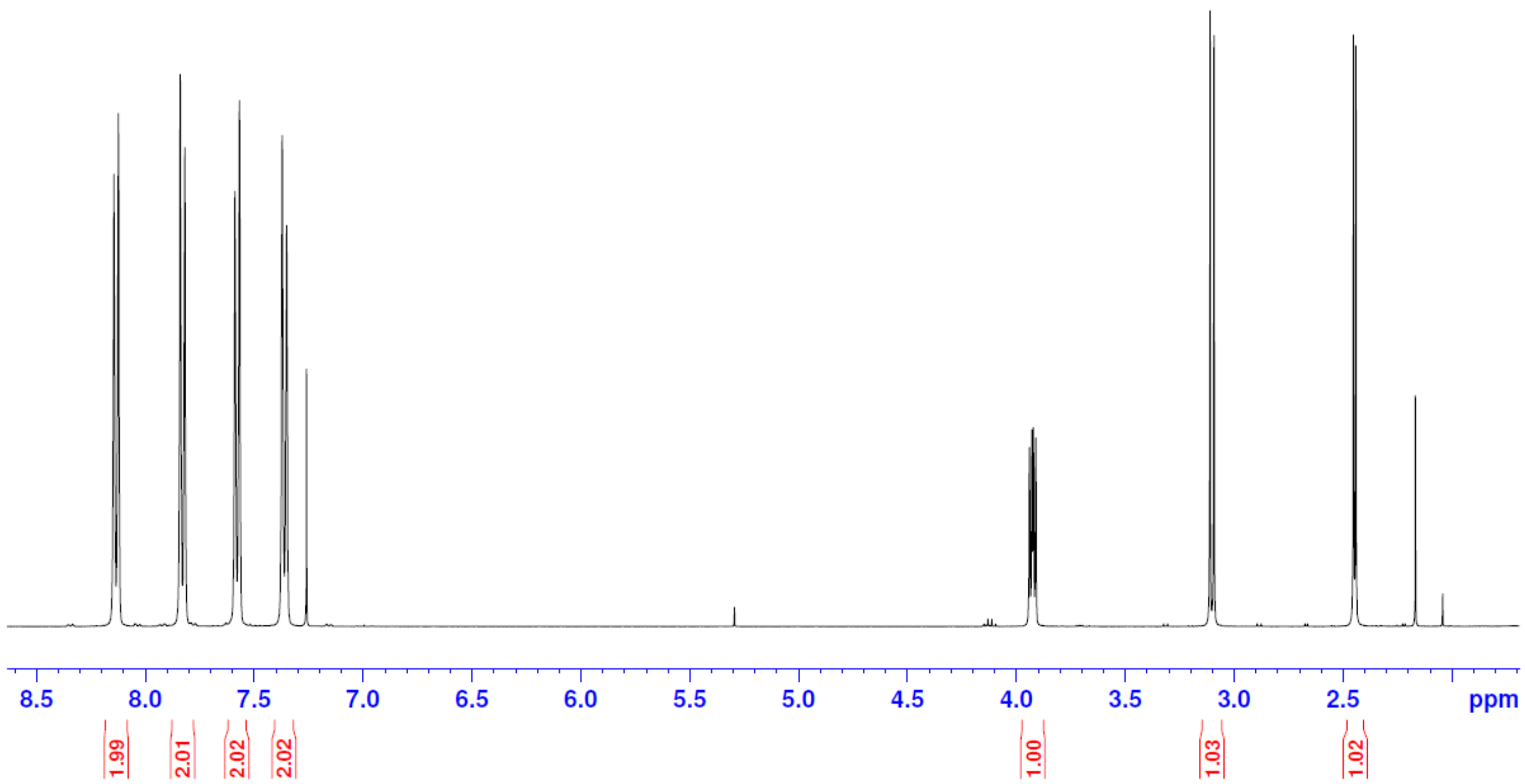

2-(4-Nitrophenyl)-1-((4-(trifluoromethyl)phenyl)sulfonyl)aziridine:

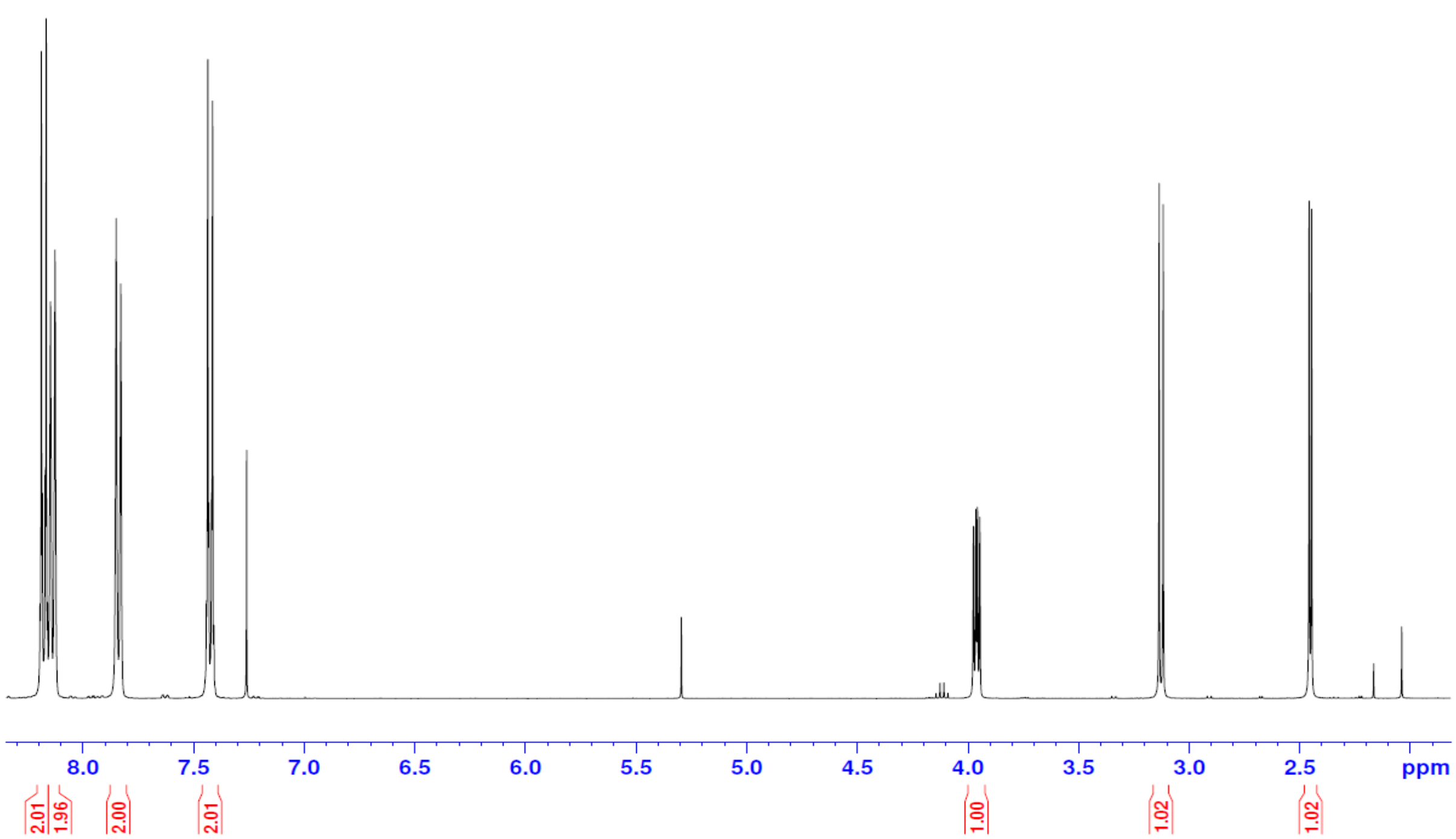

***cis*-2-Methyl-3-phenyl-1-((4-(trifluoromethyl)phenyl)sulfonyl)aziridine:**

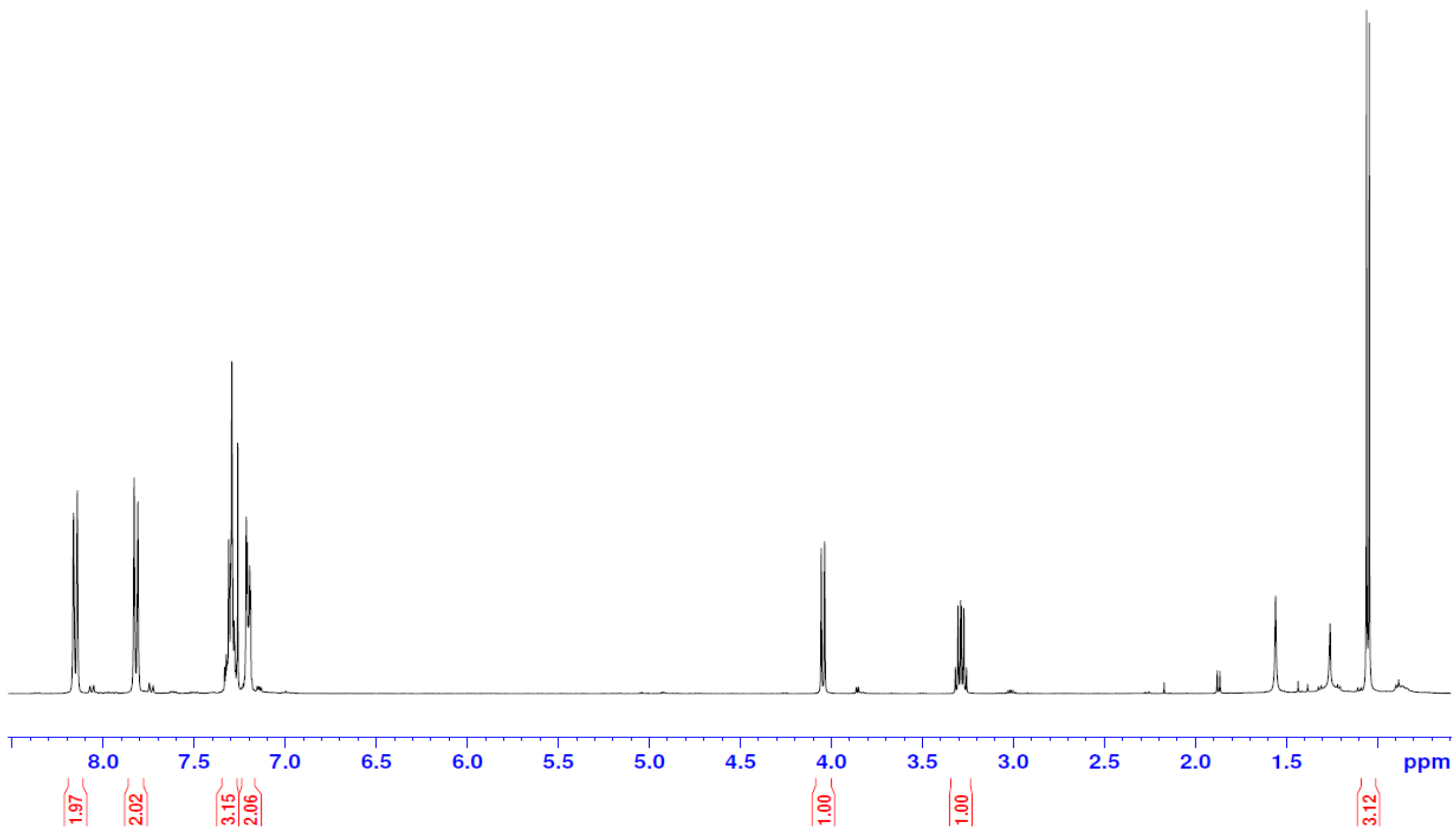

***trans*-2-Methyl-3-phenyl-1-((4-(trifluoromethyl)phenyl)sulfonyl)aziridine:**

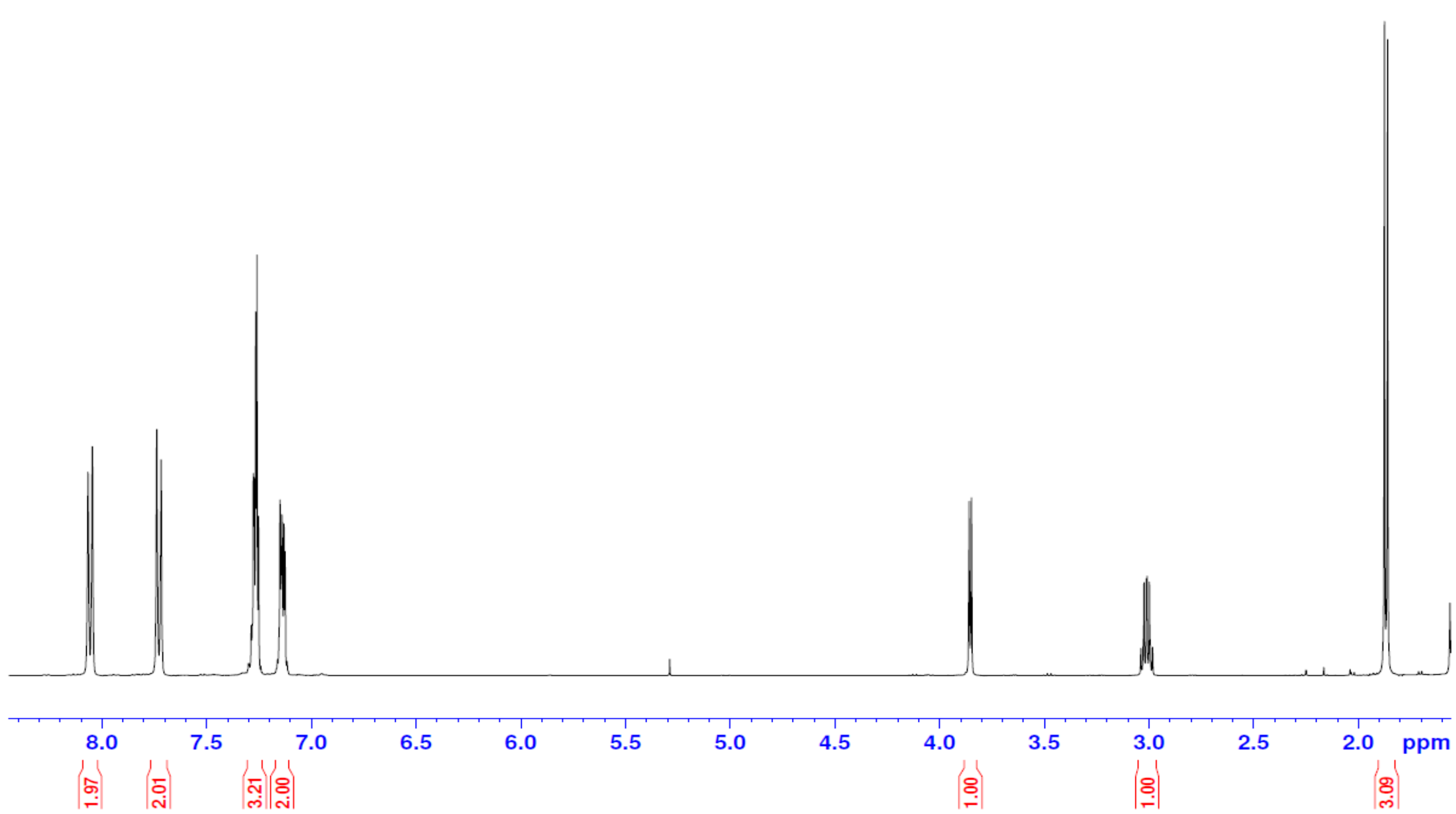

cis-2,3-Diphenyl-1-((4-(trifluoromethyl)phenyl)sulfonyl)aziridine (CD_3CN):

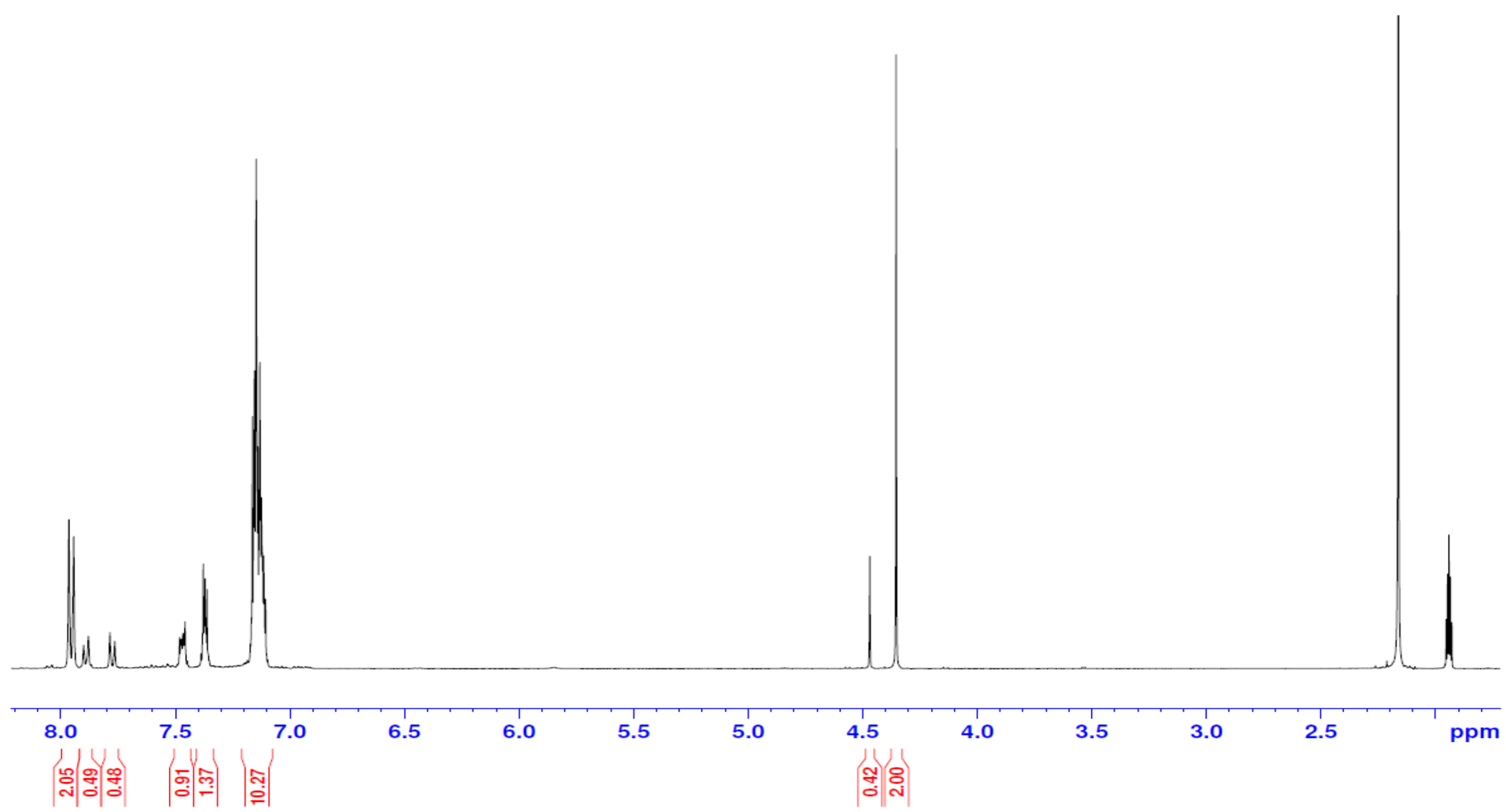

trans-2,3-Diphenyl-1-((4-(trifluoromethyl)phenyl)sulfonyl)aziridine (CD_3CN):

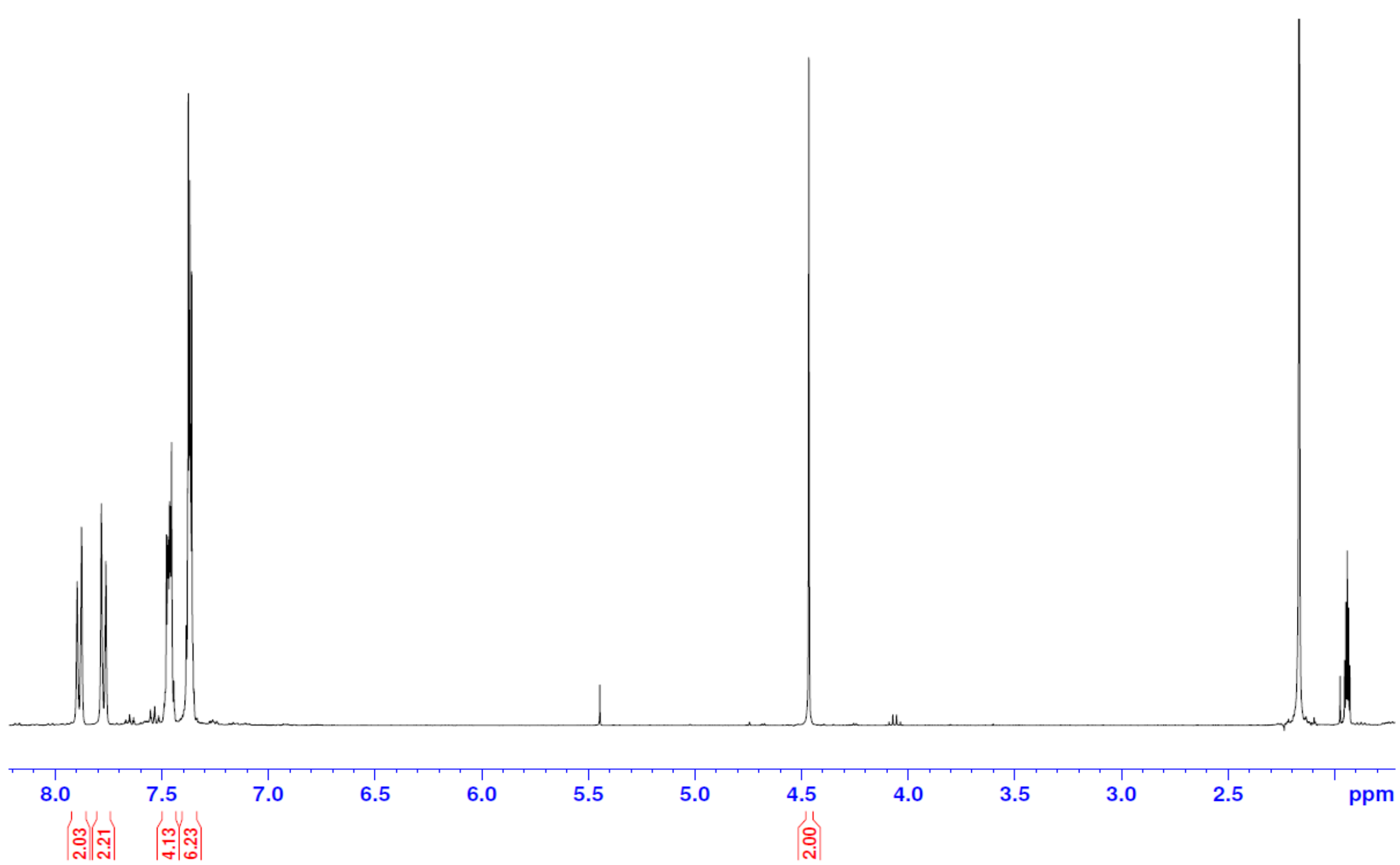

1-((4-(Trifluoromethyl)phenyl)sulfonyl)-1a,2,3,7b-tetrahydro-1H-naphtho[1,2-b]azirine:

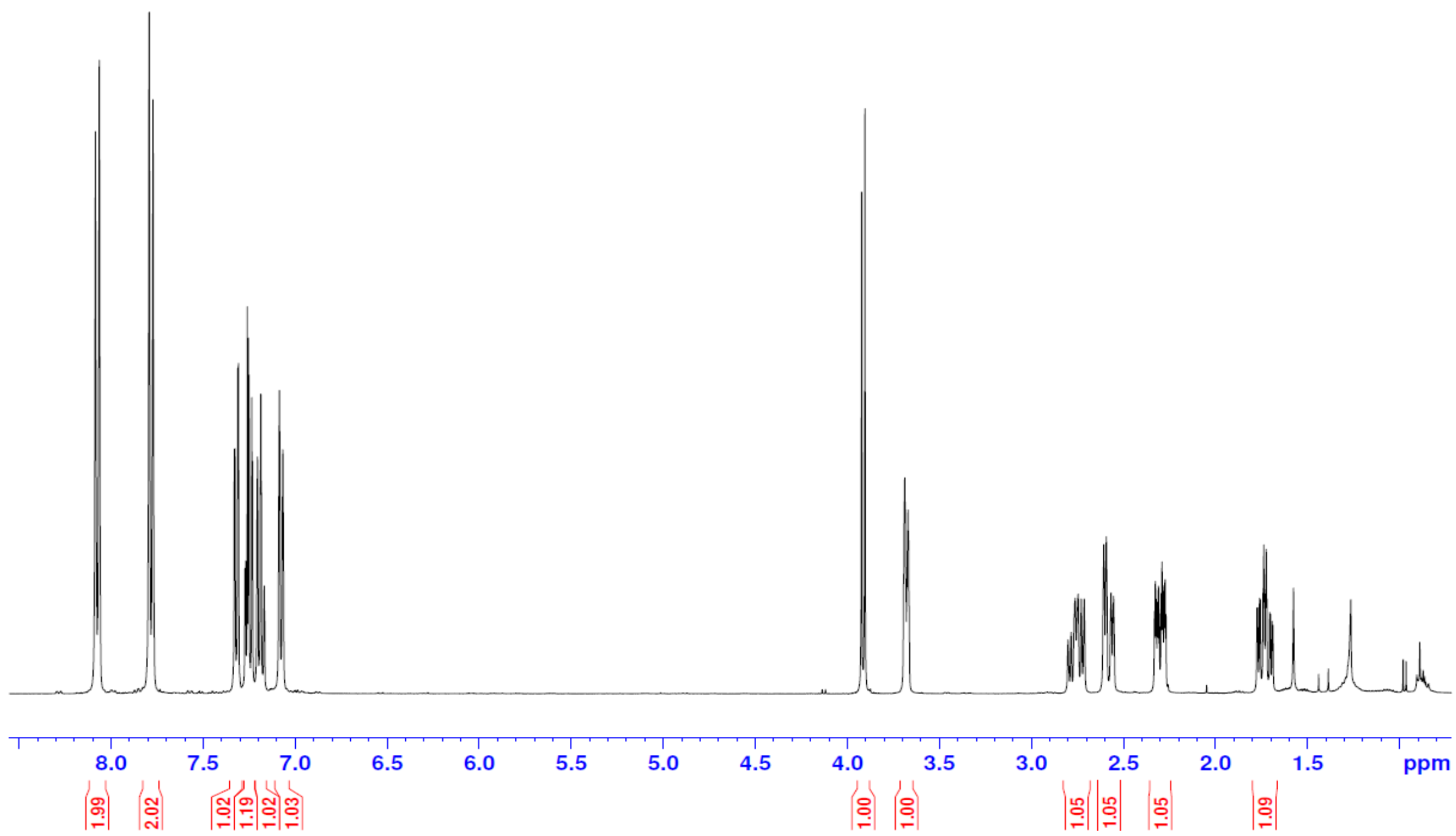

7-((4-(Trifluoromethyl)phenyl)sulfonyl)-7-azabicyclo[4.1.0]heptane:

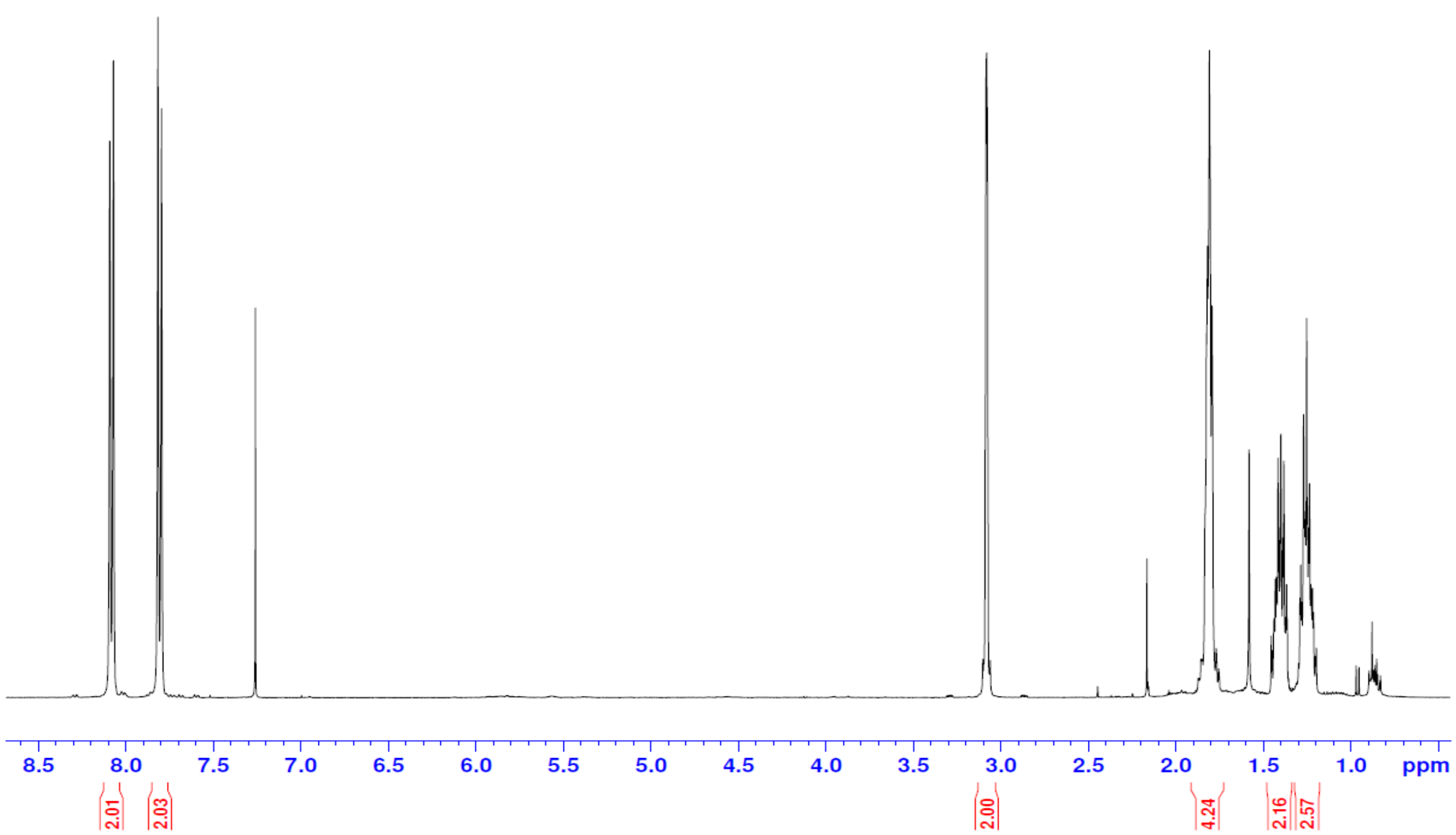

N-(Cyclohex-2-en-1-yl)-4-(trifluoromethyl)benzenesulfonamide

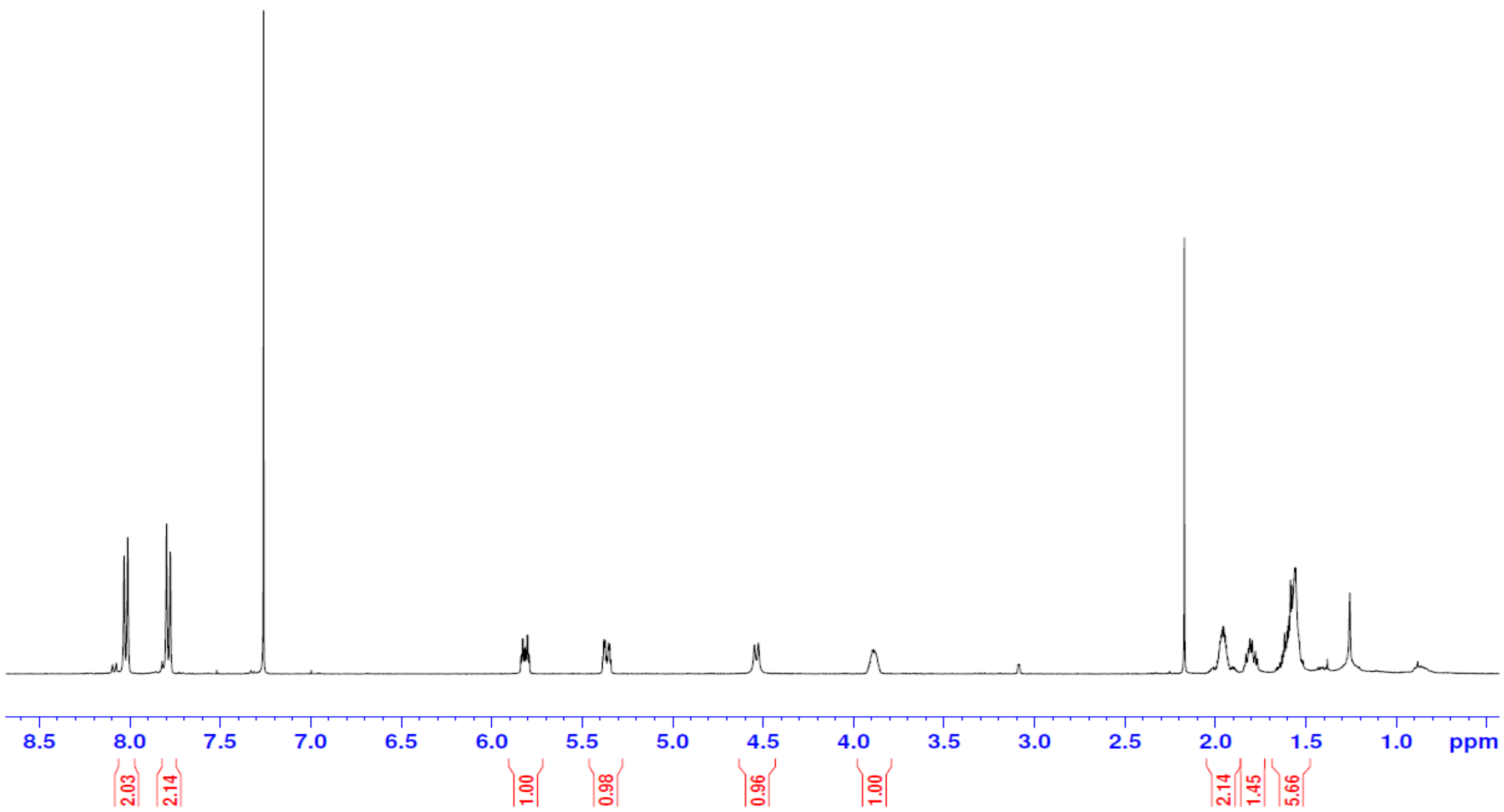

***cis*-9-((4-(Trifluoromethyl)phenyl)sulfonyl)-9-azabicyclo[6.1.0]nonane**

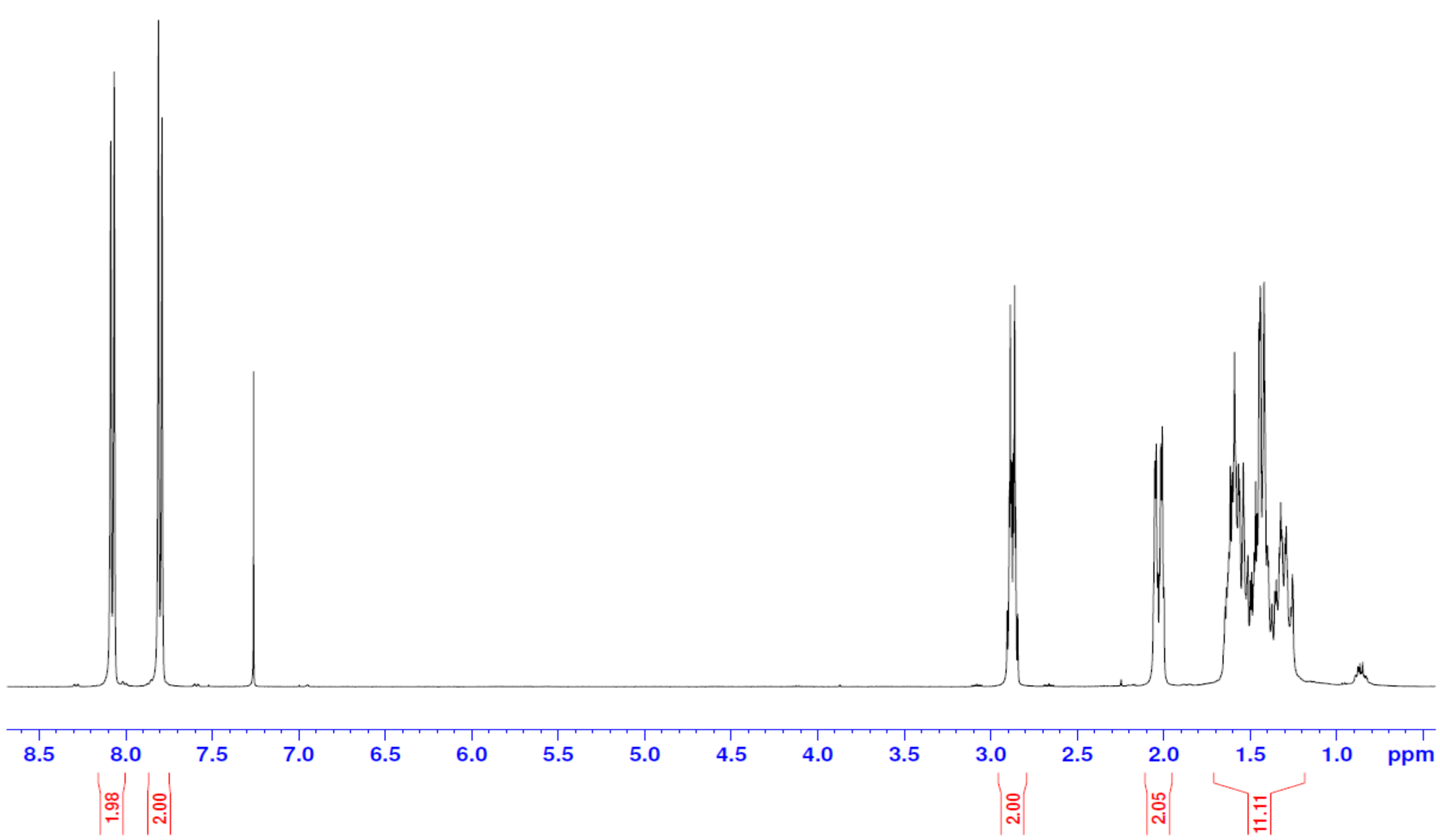

1-((4-(Trifluoromethyl)phenyl)sulfonyl)-1-azaspiro[2.5]octane:

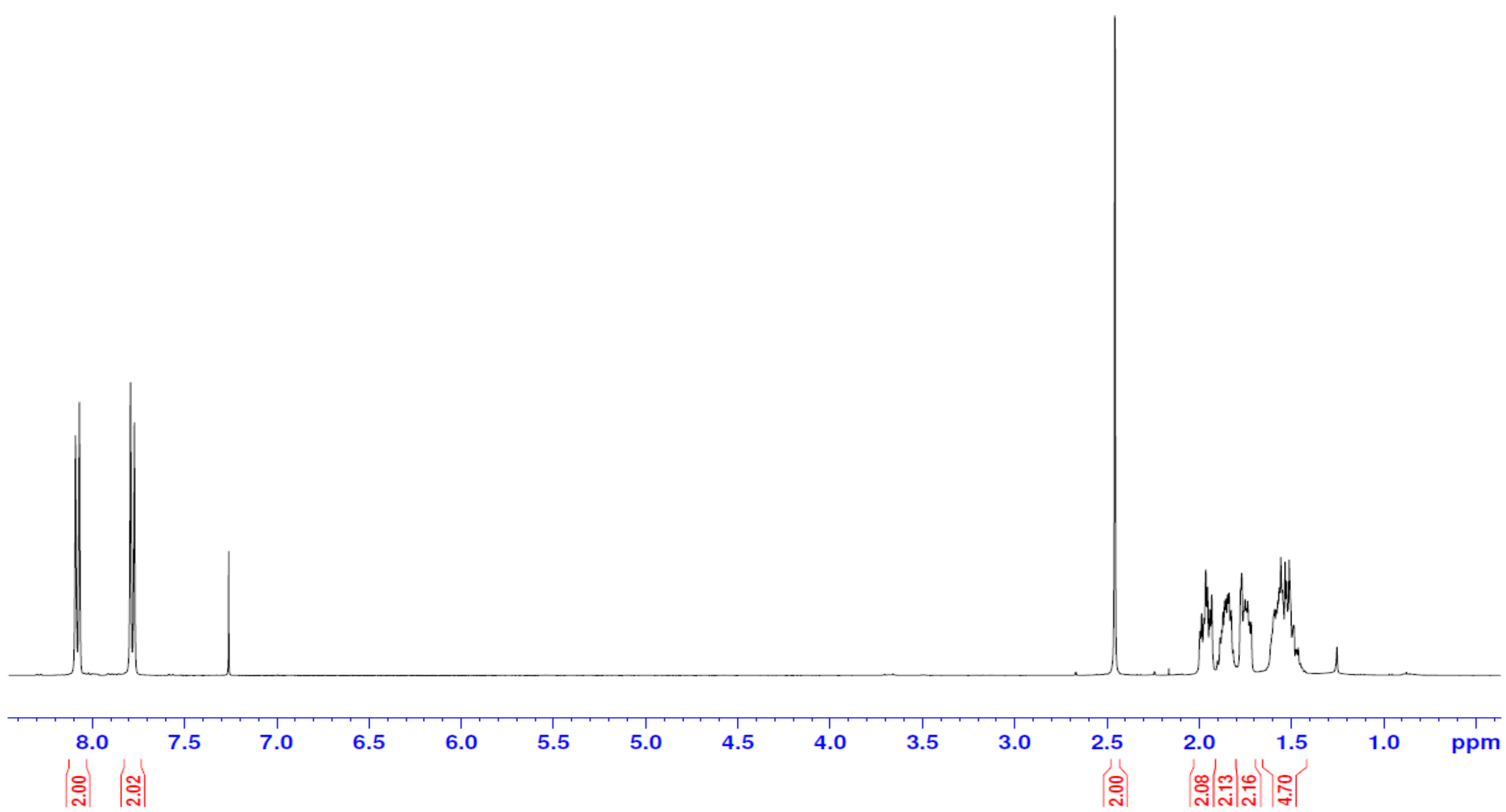

***N*-(Cyclohex-1-en-1-ylmethyl)-4-(trifluoromethyl)benzenesulfonamide:**

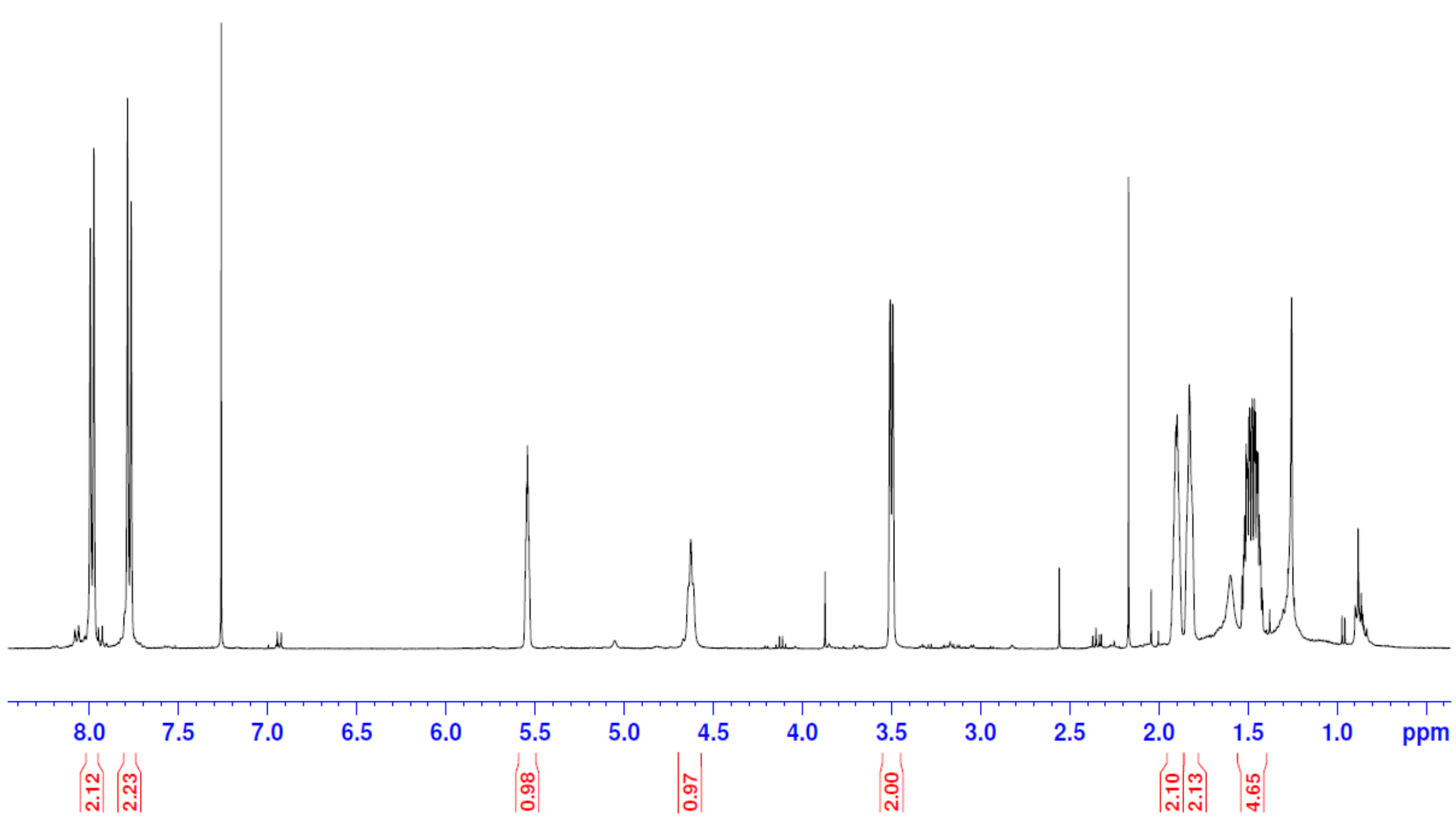

2-Butyl-1-((4-(trifluoromethyl)phenyl)sulfonyl)aziridine:

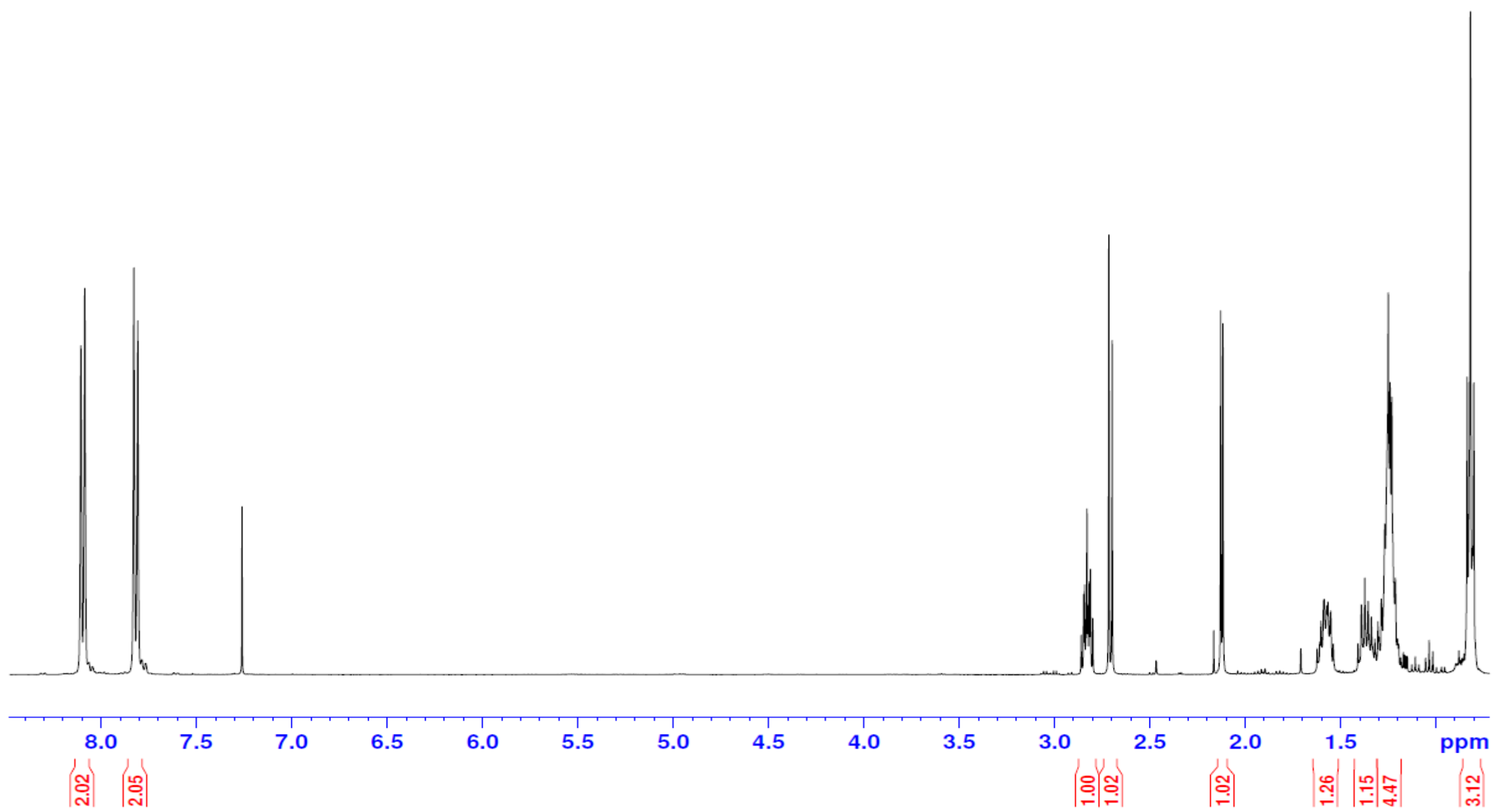

2-Heptyl-1-((4-(trifluoromethyl)phenyl)sulfonyl)aziridine:

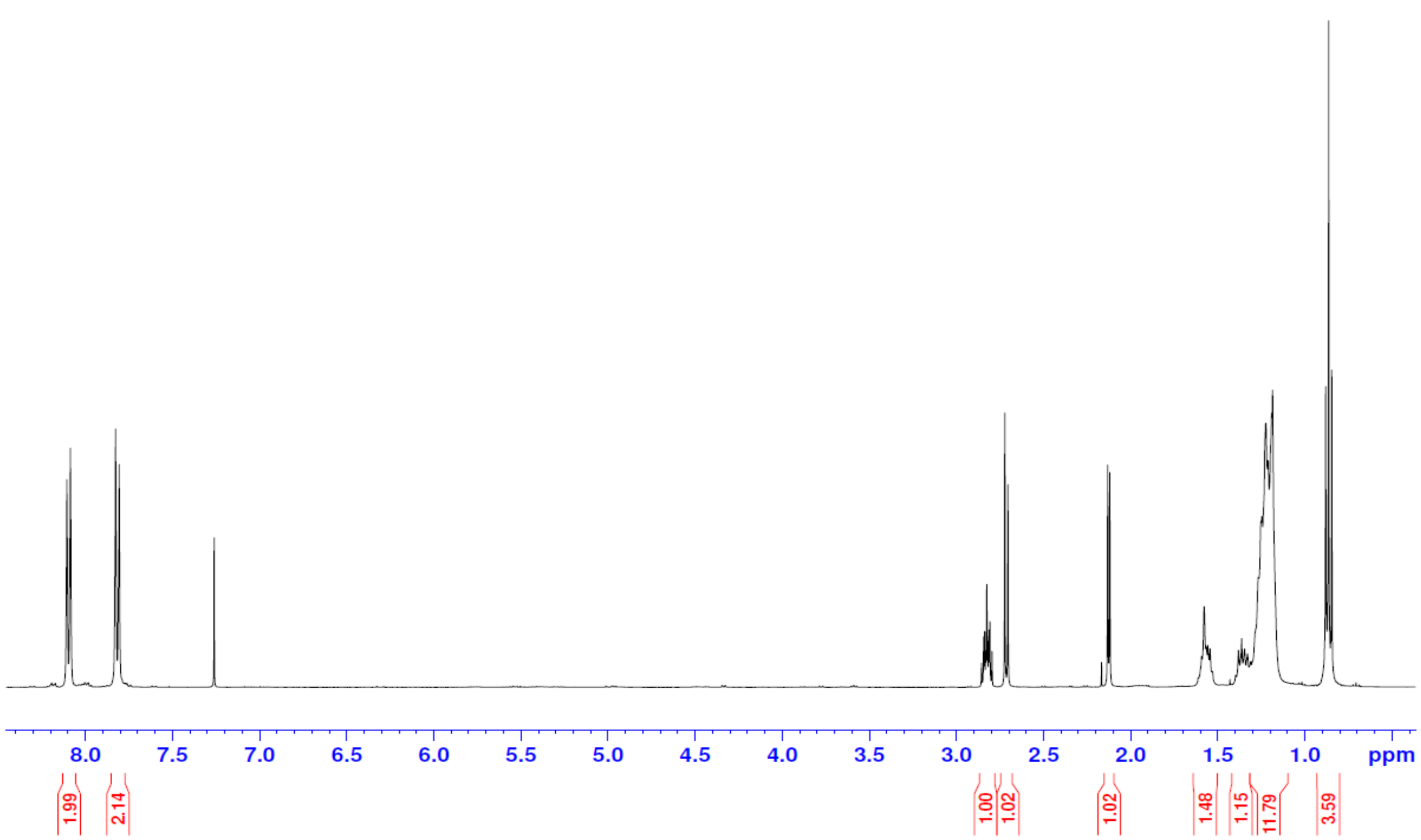

cis-2-Methyl-3-propyl-1-((4-(trifluoromethyl)phenyl)sulfonyl)aziridine:

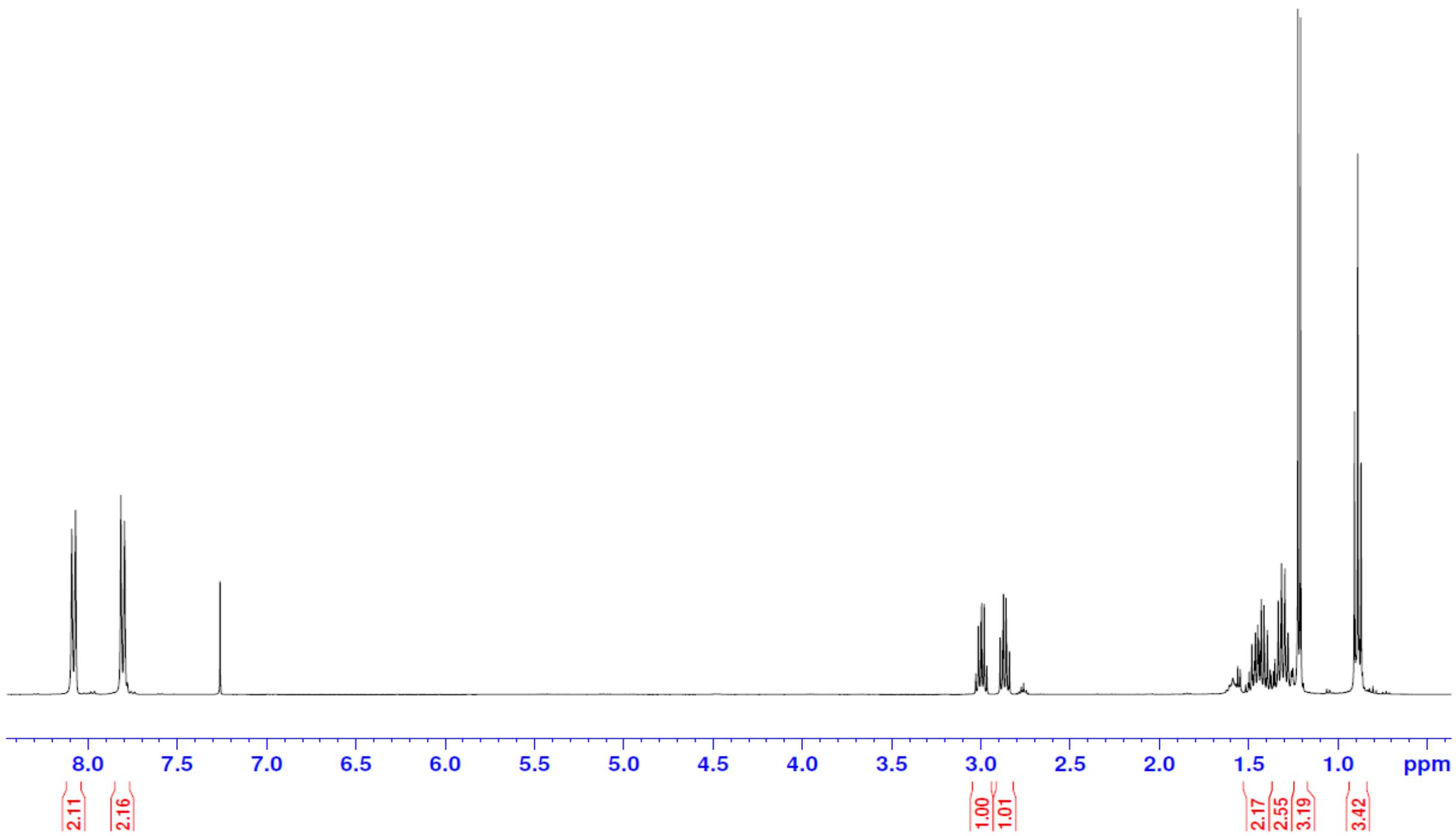

trans-2-Methyl-3-propyl-1-((4-(trifluoromethyl)phenyl)sulfonyl)aziridine:

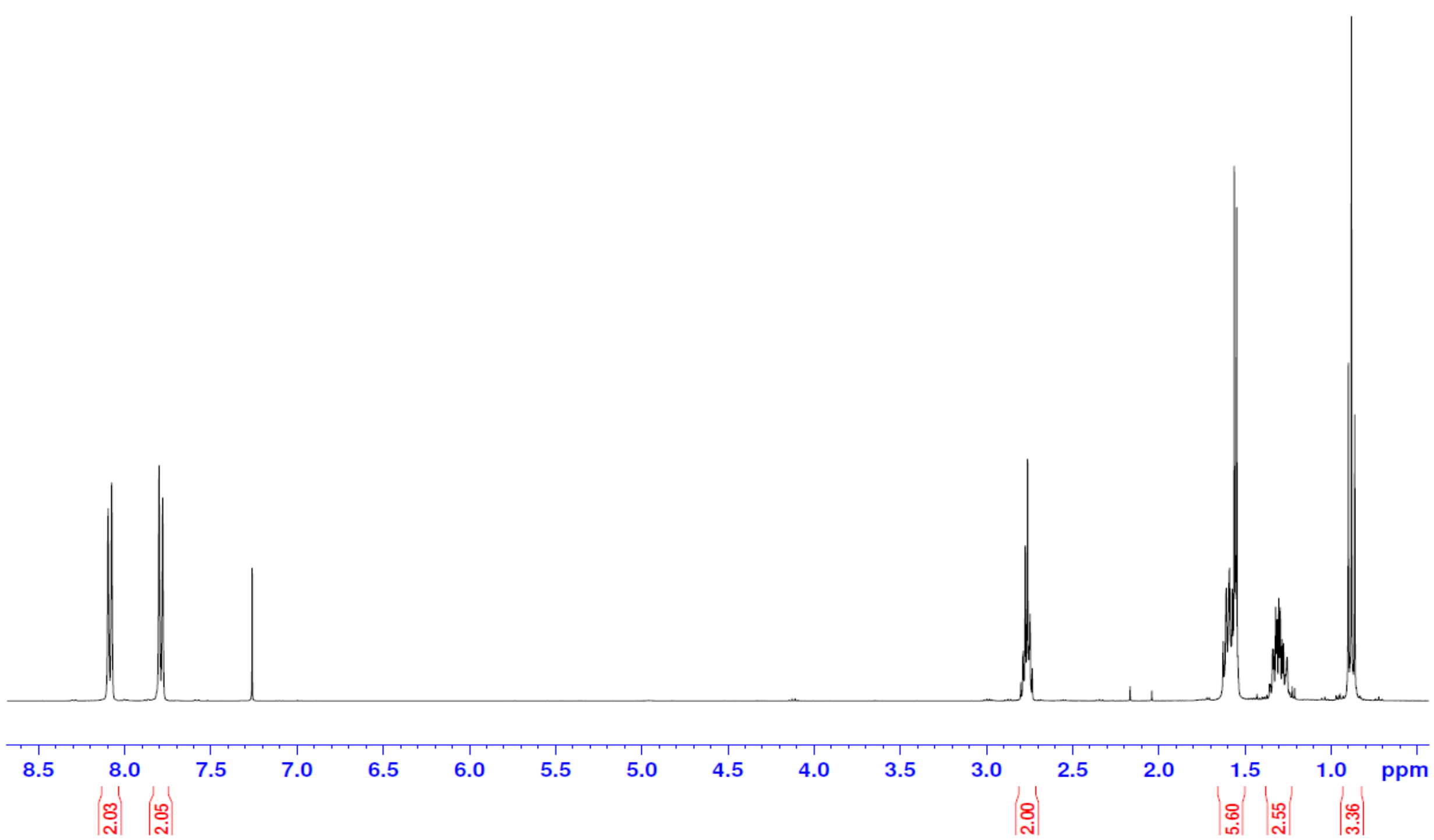

2-Benzyl-1-((4-(trifluoromethyl)phenyl)sulfonyl)aziridine:

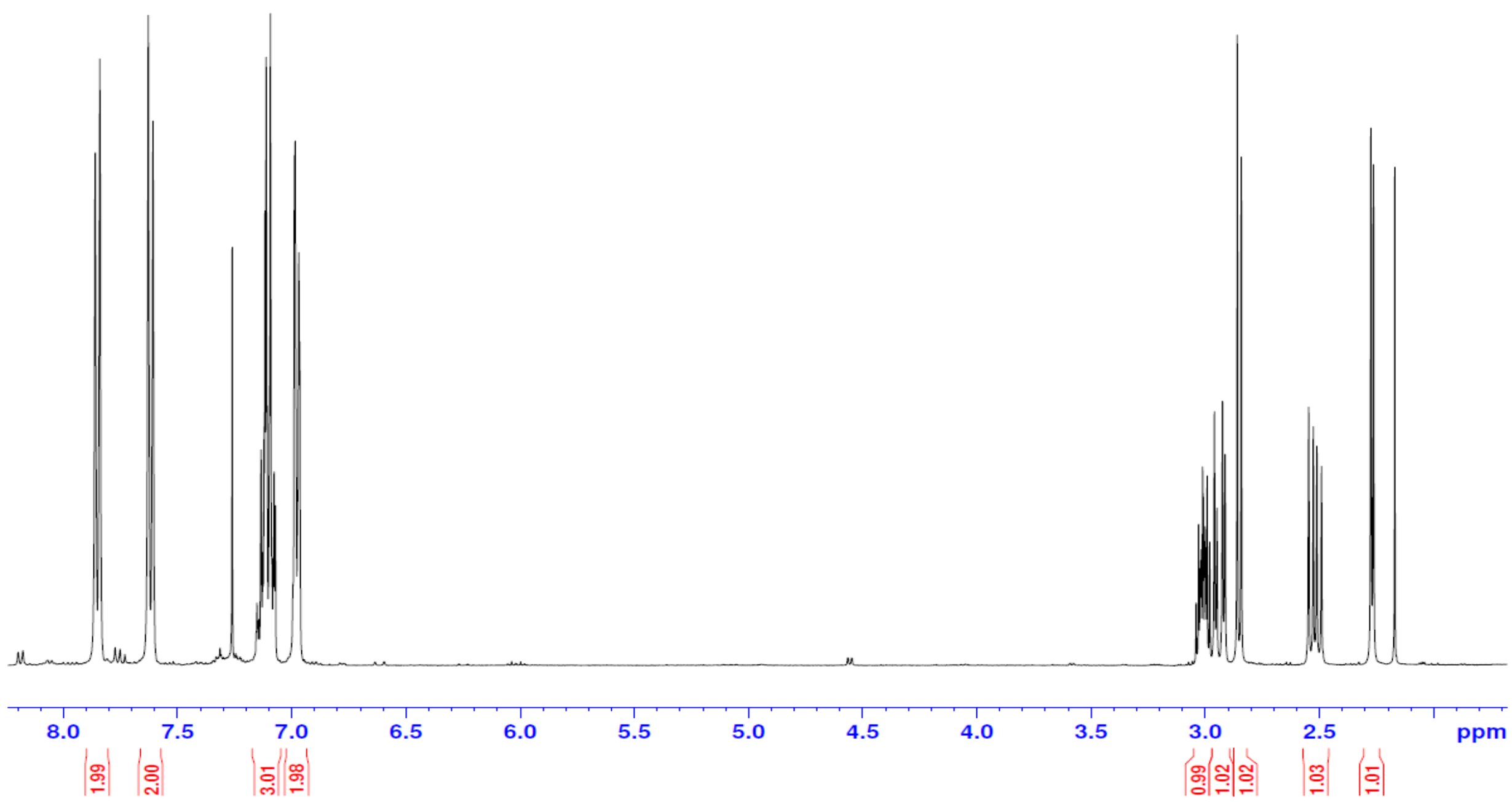

2-Phenethyl-1-((4-(trifluoromethyl)phenyl)sulfonyl)aziridine:

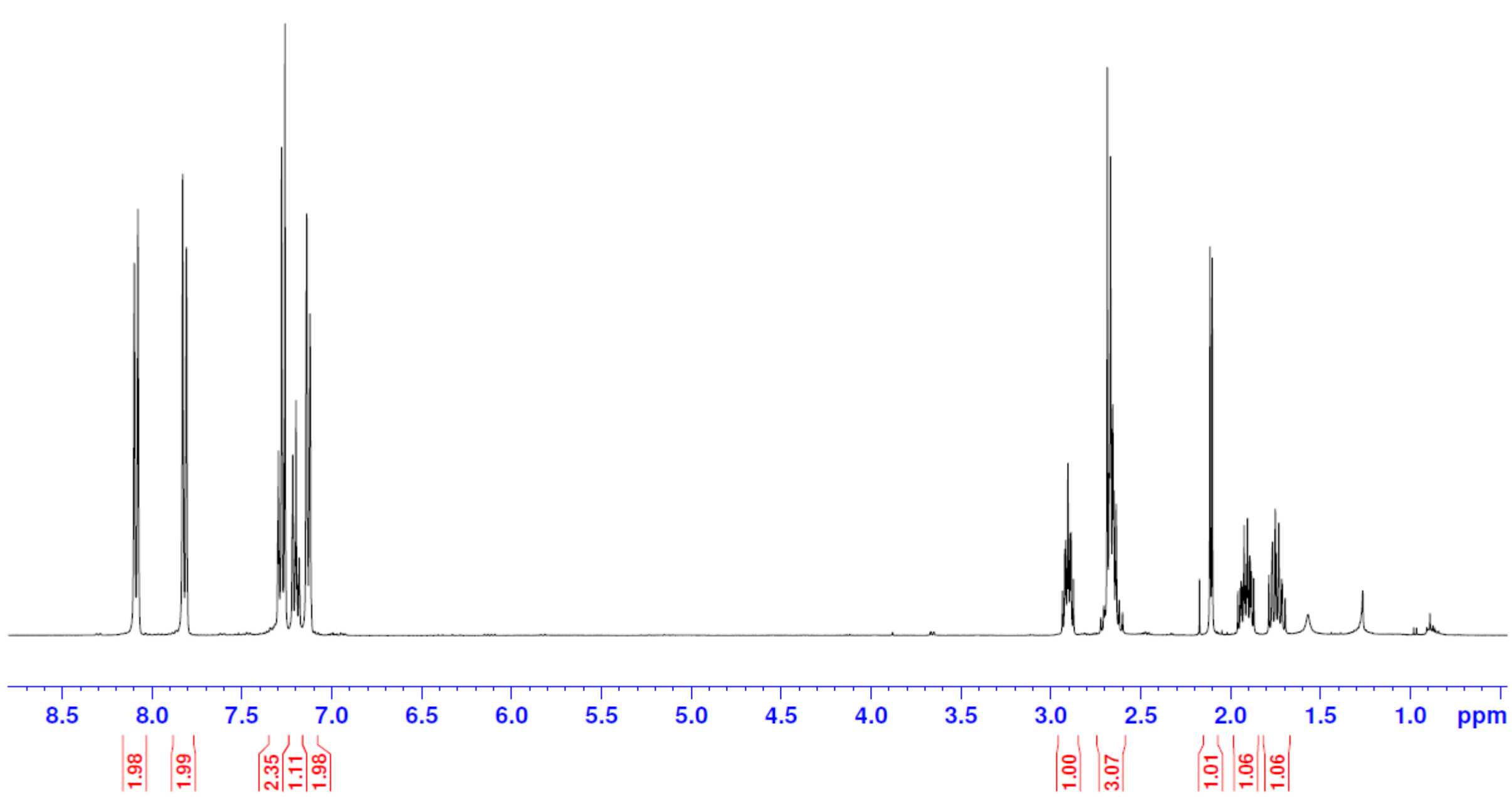

N-(2-hydroxy-2-phenylpropyl)-4-(trifluoromethyl)benzenesulfonamide with contribution from 4-(Trifluoromethyl)benzenesulfonamide

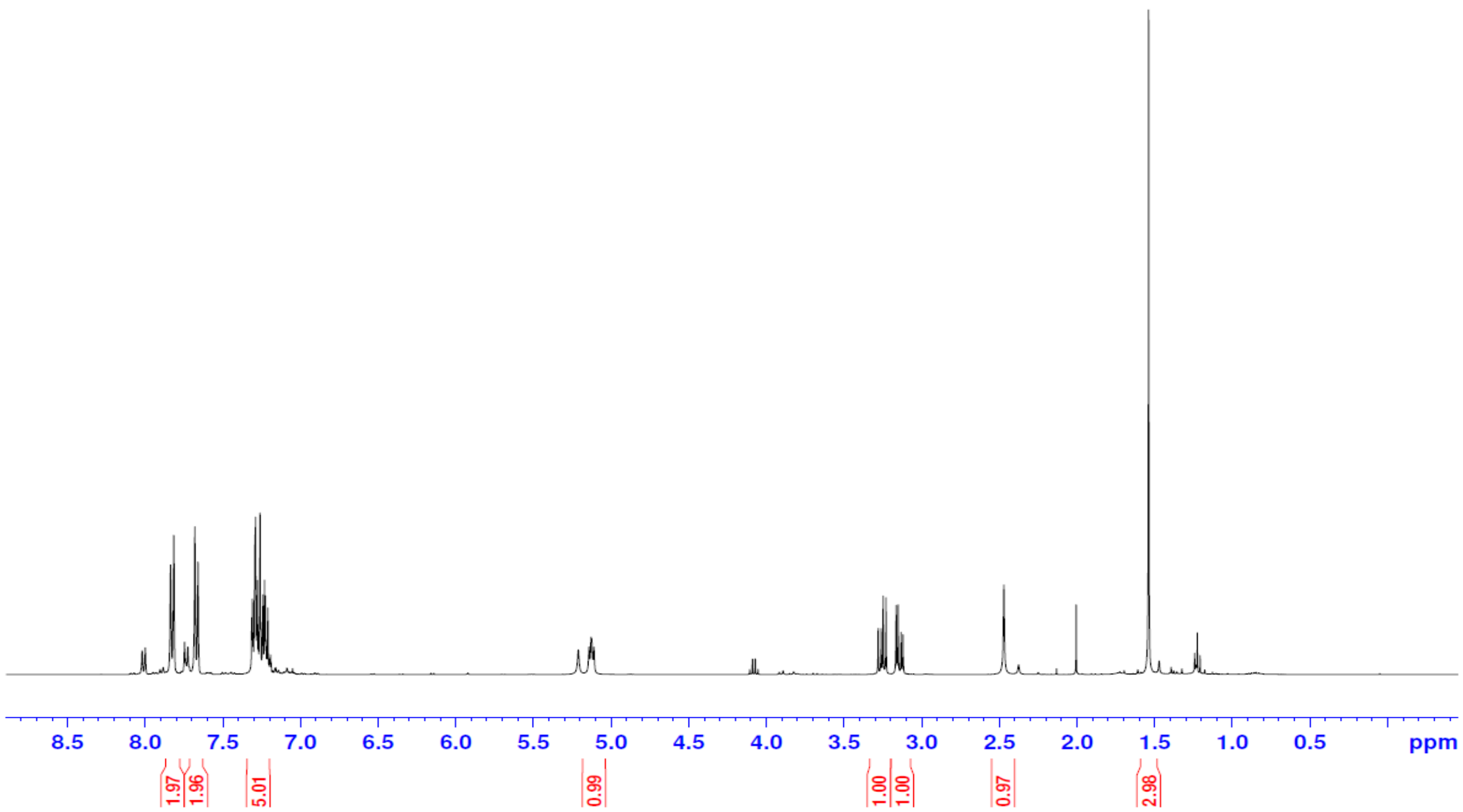

References

(1) Bruker's SAINT, SADABS, SHELXTL-PLUS, **2008**, Bruker AXS Inc., Madison, Wisconsin, USA.

(2) ChrysAlisPro version 1.171.43.144a, 2024, Rigaku Oxford Diffraction, Yarnton, Oxfordshire, England.

(3) Sheldrick, G. M. A short history of SHELX. *Acta Cryst*. **2008**, *A64*, 112.

(4) Sheldrick, G.M.; Hubshle, C. B.; Dittrich, B. Shelxle: A Qt graphical user interface for SHELXL. *J. Appl. Cryst*. **2011**, *44*, 1281–1284.

(5) JoVE Science Education Database. Inorganic Chemistry. The Evans Method. JoVE, Cambridge, MA, (2023).
